# Precise AIE‐Based Ternary Co‐Assembly for Saccharide Recognition and Classification

**DOI:** 10.1002/advs.202405613

**Published:** 2024-08-28

**Authors:** Yongxin Chang, Juan Shao, Xinjia Zhao, Haijuan Qin, Yanqing Du, Junrong Li, Qiongya Li, Wenjing Sun, Guoxiong Wang, Guangyan Qing

**Affiliations:** ^1^ State Key Laboratory of Medical Proteomics National Chromatographic R. & A. Center CAS Key Laboratory of Separation Science for Analytical Chemistry Dalian Institute of Chemical Physics Chinese Academy of Sciences Dalian 116023 P. R. China; ^2^ State Key Laboratory of Catalysis Dalian Institute of Chemical Physics Chinese Academy of Sciences Dalian 116023 P. R. China; ^3^ Research Centre of Modern Analytical Technology Tianjin University of Science and Technology Tianjin 300457 P. R. China; ^4^ Department of Pharmaceutical Sciences Inner Mongolia Medical University Hohhot 010110 P. R. China

**Keywords:** aggregation‐induced emission, array sensor, co‐assembly, saccharide recognition

## Abstract

Saccharides are involved in nearly all life processes. However, due to the complexity and diversity of saccharide structures, their selective recognition is one of the most challenging tasks. Distinct from conventional receptor designs that rely on delicate and complicated molecular structures, here a novel and precise ternary co‐assembled strategy is reported for achieving saccharide recognition, which originates from a halogen ions‐driven aggregation‐induced emission module called *p*‐Toluidine, *N, N*′‐1‐propen‐1‐yl‐3‐ylidene hydrochloride (PN‐Tol). It exhibits ultra‐strong self‐assembly capability and specifically binds to 4‐mercaptophenylboronic acid (MPBA), forming highly ordered co‐assemblies. Subsequent binding of various saccharides results in heterogeneous ternary assembly behaviors, generating cluster‐like, spherical, and rod‐like microstructures with well‐defined crystalline patterns, accompanied by significant enhancement of fluorescence. Owing to the excellent expandability of the PN module, an array sensor is constructed that enables easy classification of diverse saccharides, including epimer and optical isomers. This strategy demonstrates wide applicability and paves a new avenue for saccharide recognition, analysis, and sequencing.

## Graphical abstract

Saccharide recognition is crucial in various biological processes. This study proposes an innovative strategy called ternary co‐assembly, enabling the creation of multifunctional architectures with tailored properties using only basic molecular building blocks, thereby eliminating the need for complex molecular synthesis. Consequently, this approach successfully achieved precise detection and discrimination of various chiral, linkage, and structural isomers of saccharide.

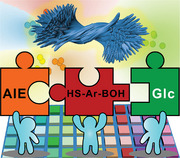

## 1 Introduction

**Figure 1:**
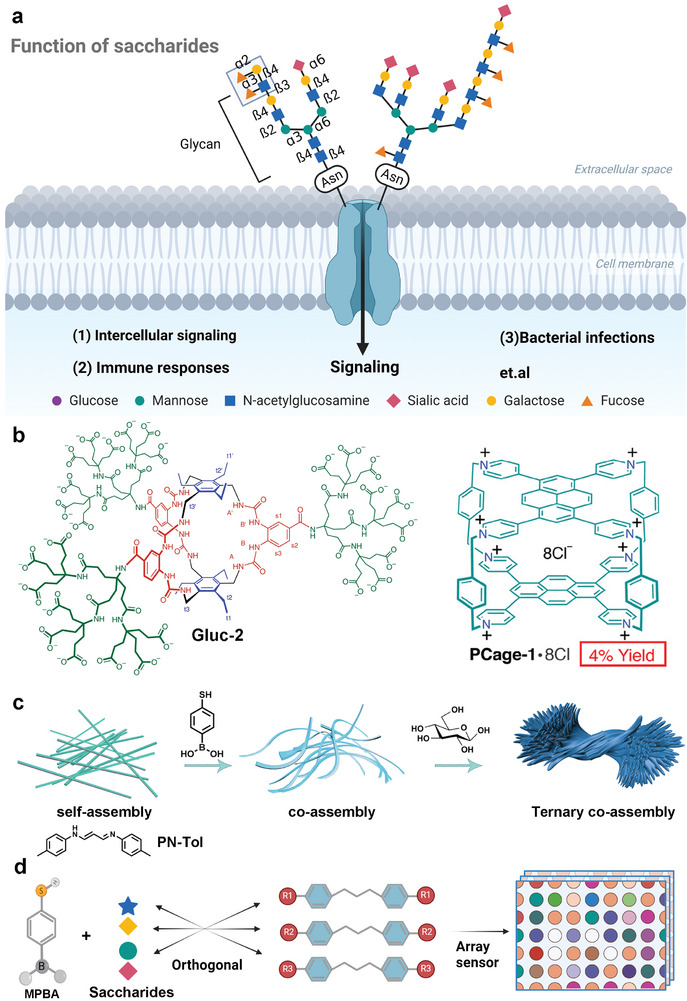
Significance of saccharide recognition and our design idea. a) Graphic illustration of glycan compositions and structures and their significance in biological systems; b) Representative synthetic ligands used for saccharide recognition; c) Illustration of sequential co‐assembly among PN‐Tol, MPBA, and saccharides; d) Construction of assay fluorescent sensors for saccharide differentiation and classification.

Carbohydrates, also known as saccharides, are one of the four major biological substances in nature,^1^ alongside nucleic acids, proteins, and lipids. Saccharides play significant roles in the onset and progression of various human diseases,^[^ ^1^ ^]^ including diabetes, pathogen infections,^[^ ^2^ ^]^ and tumor metastasis.^[^ ^3^ ^]^ Additionally, saccharides are involved in almost all crucial biological processes, such as fertilization,^[^ ^4^ ^]^ intercellular signaling,^[^ ^5^ ^]^ immune responses,^[^ ^6^ ^]^ and viral and bacterial infections.^[^ ^7^ ^]^ The complex structural heterogeneity of saccharides (**Figure** **1**a) allows them to carry a vast amount of information,^[^ ^8^ ^]^ far exceeding the combined amount of information carried by proteins and nucleotides. Consequently, the precise identification of saccharides holds revolutionary significance for the development of carbohydrate‐based drugs and vaccines,^[^ ^9^ ^]^ and it will drive significant innovation and market growth in the industry.^[^ ^10^ ^]^ In recent decades, chemists have devoted substantial efforts to developing synthetic receptors capable of selectively recognizing saccharides or their derivatives.^[^ ^11^ ^]^ However, due to the complexity and diversity of mono‐saccharide and oligo‐saccharide structures, including complex mono‐saccharide compositions, sequences, linkage types, branched structures, and anomeric stereochemistry, achieving selective saccharide recognition using synthetic receptors is highly challenging, particularly when it comes to mono‐saccharide recognition, as their structures only exhibit subtle differences, often attributed to stereochemical variations caused by single hydroxyl groups.^[^ ^12^ ^]^ Despite the existence of some successful examples,^[^ ^13^ ^]^ such as the artificial receptors Gluc‐2 developed by Davis^[^ ^14^ ^]^ (Figure 1b left), which can selectively recognize glucose with an affinity (*K*a) of 18 000 M^−1^, nearly equivalent to natural receptors. Additionally, pyrene‐based, temple‐shaped receptors Pcage‐1·8Cl developed by Stoddart and colleagues can recognize multiple saccharides^[^ ^15^ ^]^ (Figure 1b right). However, the synthesis process of these receptors is generally very complex, and the yields are also extremely low. Consequently, their success usually comes at the cost of structural complexity.

On the other hand, self‐assembly is a fundamental process in life systems, playing a crucial role in the formation and organization of structures,^[^ ^16^ ^]^ such as phospholipid bilayer,^[^ ^17^ ^]^ and DNA duplex,^[^ ^18^ ^]^ at both the molecular and macroscopic levels. Self‐assembly often relies on molecular recognition as a driving force for organizing components into larger structures,^[^ ^19^ ^]^ which inspires scientists to develop novel chemosensors based on self‐assembly.^[^ ^20^ ^]^ The selective interaction between the assembled components and guest molecules, as well as subtle differences among the guests, could be recognized, translated, amplified, and transferred into remarkable variations in the macromolecular,^[^ ^21^ ^]^ supramolecular,^[^ ^22^ ^]^ and even macroscopic properties of the materials.^[^ ^23^ ^]^ This approach substantially improves the sensitivity and selectivity of the sensors. However, in practice, it is challenging to discover a pair of species capable of undergoing such dedicated co‐assembly and to precisely control the self‐assembly process.^[^ ^24^ ^]^ Especially in the case of ternary co‐assembly, it involves multiple interactions among three different components.^[^ ^25^ ^]^ Understanding and balancing these interactions is even more complex. Nevertheless, ternary co‐assembly is ubiquitous (collagen,^[^ ^26^ ^]^ lipoproteins,^[^ ^27^ ^]^ and immunological synapse,^[^ ^28^ ^]^ et al.) and quite enticing, it allows for the creation of complex and multifunctional structures with enhanced performance and accuracy, which cannot be achieved by binary or individual component assemblies. In this respect, saccharide‐selective sensors based on co‐assembly are rarely reported, owing to the inherent complexity of saccharides as well as the difficulties in saccharide recognition.^[^ ^29^ ^]^

To tackle this challenge, we propose a novel design strategy based on ternary co‐assembly, involving the utilization of an aggregation‐induced emission (AIE) molecule (i.e., PN‐Tol), 4‐mercaptophenylboronic acid, and various saccharides. This approach achieves highly efficient saccharide recognition and classification by taking advantage of the remarkable differences in self‐assembled morphology and AIE fluorescent enhancement. First, PN‐Tol was discovered to possess a typical AIE characteristic and an ultra‐strong self‐assembled capacity. Its crystal stacking model revealed that the halogen ion served as the driving force for molecular aggregation. Further research revealed that PN‐Tol could specifically bind and co‐assemble with MPBA, resulting in a 2.9‐fold enhancement in fluorescence. In comparison, PN‐Tol exhibited a negligible response to the other 17 kinds of molecules. Importantly, the PN‐Tol@MPBA co‐assembly further formed ordered ternary assemblies with various mono‐, di‐, and tri‐saccharides (Figure 1c), leading to remarkable fluorescence enhancement. Based on the diverse assembled morphologies, various chiral, linkage, and structural isomers of saccharides could be easily discriminated. Furthermore, we introduced six derivatives of PN to construct an array sensor (Figure 1d), which successfully achieved rapid and accurate differentiation and classification of various saccharides, even in a serum mixture with a total protein content exceeding 130‐fold. This sequential and precise ternary co‐assembly strategy, based on the non‐covalent interactions, demonstrates significant potential in saccharide recognition and discrimination. Besides, we provide a swift, visible, and highly efficient method for screening assembly components and determining optimal module combinations by taking advantage of the obvious AIE effect, which will remarkably expedite the discovery of ternary co‐assembly systems, opening up limitless possibilities for molecular self‐assembly.

## 2 Results and Discussion

### 2.1 Molecular Synthesis

**Figure 2:**
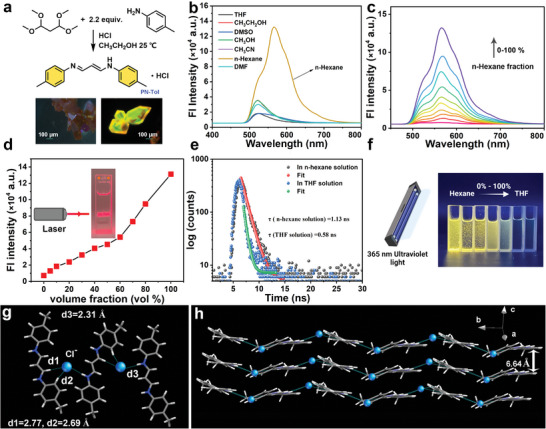
AIE properties of PN‐Tol. a) Synthetic route of PN‐Tol, inset displays fluorescence image of PN‐Tol crystal and crystal morphology; b) Fluorescence emission spectra of PN‐Tol (33 µm) in different solvents at 25 °C, excitation wavelength (Ex): 293 nm; c,d) Fluorescence spectra (c) and changes in fluorescent intensity at 565 nm (d) of PN‐Tol (33 µm) in THF/*n*‐hexane mixed solution with different volume fraction of *n*‐hexane, inset shows the Tyndall effect; e) Fluorescence decay curves and corresponding lifetime values of PN‐Tol (150 µm) in *n*‐hexane and THF at 25 °C, Ex: 293 nm; f) Photograph of PN‐Tol (0.1 mM) in different ratios of THF/*n*‐hexane mixed solutions under 365 nm ultraviolet (UV) light irradiation; g,h) Crystal packing models of PN‐Tol viewed from different angles, where gray, white, and blue colors represent C, H, and N atoms, respectively, and the blue‐green spheres represent Cl ions. The d1, d2, and d3 represent the bond lengths.

PN‐Tol was synthesized through a condensation reaction between trimethoxypropane and *p*‐toluidine in anhydrous ethanol (**Figure** **2**a), yielding a high product yield (≈84%) with no further purification required. The synthesis and characterization details of PN‐Tol can be found in Scheme S2 (Supporting Information). Simultaneously, the PN skeleton demonstrates excellent substrate versatility, allowing for the facile generation of various PN derivatives by changing the substitution groups on the phenyl ring. In this regard, PN‐Boctyl, PN‐TPE, PN‐BP, PN‐BIP, and PN‐BA were synthesized for the construction of the array sensor. The corresponding information related to structural characterization can be found in the Supporting Information.

### 2.2 Research on Photophysical Properties

First, the spectroscopic properties of PN‐Tol were investigated in various solutions. The results revealed that PN‐Tol exhibited weak fluorescence emission in methanol (CH_3_OH), ethanol, dimethyl sulfoxide (DMSO), *N, N*‐dimethylformamide (DMF), acetonitrile (CH_3_CN), and tetrahydrofuran (THF) with an emission peak at 565 nm. Furthermore, the UV‐vis absorption spectra obtained from these solvents indicate that PN‐Tol exhibits solvent‐dependent characteristics. For instance, plotting the stokes shift obtained in CH_3_OH, THF, dichloromethane, and CH_3_CN against the solvent orientation polarizability (Δ*f*) in the Lippert‐Mataga equation^[^ ^30^ ^]^ shows a good linear relationship, with a slope of 5338 cm^−1^ (Figure S1, Supporting Information). However, when PN‐Tol was dispersed in *n*‐hexane, a large amount of solid precipitate was observed in the solution, accompanied by a strong fluorescence emission (Figure 2b). This indicated that PN‐Tol had AIE characteristics. Subsequently, we further evaluated the AIE effect of PN‐Tol by using mixed solvents of *n*‐hexane/THF with different volume fractions as the solvent system. As shown in Figure 2c,d, the fluorescent intensity of the THF solution of PN‐Tol was very weak. However, as the proportion of *n*‐hexane in the mixed solvent increased, the fluorescent intensity gradually enhanced. When the proportion of *n*‐hexane reached 60%, the fluorescent intensity of PN‐Tol experienced a sharp increase, reaching a maximum value ≈16 times higher than that of the pure THF solution. These findings clearly indicated that PN‐Tol possessed a typical AIE feature. At the same time, the luminescent behavior of PN‐Tol in H_2_O was investigated. Since PN‐Tol is a hydrochloride salt with some solubility in H_2_O, its fluorescence intensity in H_2_O is intermediate between that in *n*‐hexane and THF. When increasing the volume fraction of H_2_O in a mixed solution with THF, a fluorescence enhancement is still observed, indicating that PN‐Tol retains its AIE properties in H_2_O (Figure S2, Supporting Information).

Time‐resolved fluorescence (TRF), utilizing up‐conversion technology, further confirmed the spectral difference between the aggregated and solution states of PN‐Tol. As shown in Figure 2e, the fluorescence lifetime (τ) of PN‐Tol in the THF solution is 0.58 ns, whereas the τ value increases to 1.13 ns in the *n*‐hexane solution, with no discernible delayed component, thus indicating that the emission is solely triggered by the rapid decay of the singlet state. In addition, the formation of the aggregates was validated through the Tyndall effect experiment. When a laser beam passes through the PN‐Tol solution, a bright “red light path” can be observed from the direction of perpendicular incident light (inset of Figure 2d). Furthermore, UV irradiation of *n*‐hexane/THF mixed solvents with different *n*‐hexane volume fractions (Figure 2f) showed that the fluorescence gradually weakened as the volume fraction of THF increased. Simultaneously, numerous tiny yellow microparticles could be observed with the naked eye, confirming the AIE nature of PN‐Tol.

The single‐crystal structure of PN‐Tol was successfully obtained due to its high crystallinity. Initially, the spatial arrangement of the crystal was analyzed, revealing that it belonged to a non‐centrosymmetric orthorhombic space group (P2_1_/n, number 14) (Table S1, Supporting Information).^[^ ^31^ ^]^ Surprisingly, adjacent molecules were connected by Cl^−^ ions, forming three groups of intermolecular interactions, including two strong C‐H…Cl bonds with lengths of 2.77 (d1) and 2.69 Å (d2), as well as an N‐H…Cl bond with a distance of 2.31 Å (d3) (Figure 2g). Driven by these halogen ions, two adjacent PN‐Tol molecules were connected, resulting in the formation of dimers. Subsequently, two adjacent dimers were connected side‐by‐side in space along the b‐axis, constructing a 3D spatial structure with a zigzag shape, with an interplanar separation of 6.64 Å approximately (Figure 2h). This indicated that π–π stacking that is favorable to aggregation‐caused quenching could not form efficiently. The highly ordered stacking of the molecules limited their free movement, which in turn hindered non‐radiative transitions.^[^ ^32^ ^]^ This phenomenon is consistent with the restriction of intramolecular motions (RIM) mechanism,^[^ ^33^ ^]^ which contributes significantly to the pronounced AIE effect and a high absolute quantum yield of 35.9%. Notably, unlike traditional RIM, which is typically driven by hydrogen bonding^[^ ^34^ ^]^ or the disorderly stacking of phenyl groups,^[^ ^35^ ^]^ our research revealed that Cl^−^ plays a crucial role in molecular aggregation and the induction of RIM. Besides, The research also found that not only Cl^−^ can induce molecular aggregation, but other ions including *p*‐toluenesulfonate ions, BF_4_^−^, Br^−^, and F^−^ can also cause molecular aggregation leading to intense aggregation‐induced emission (Figure S3, Supporting Information) and blue shift of emission wavelength.

### 2.3 Selective Binding between PN‐Tol and MPBA

**Figure 3:**
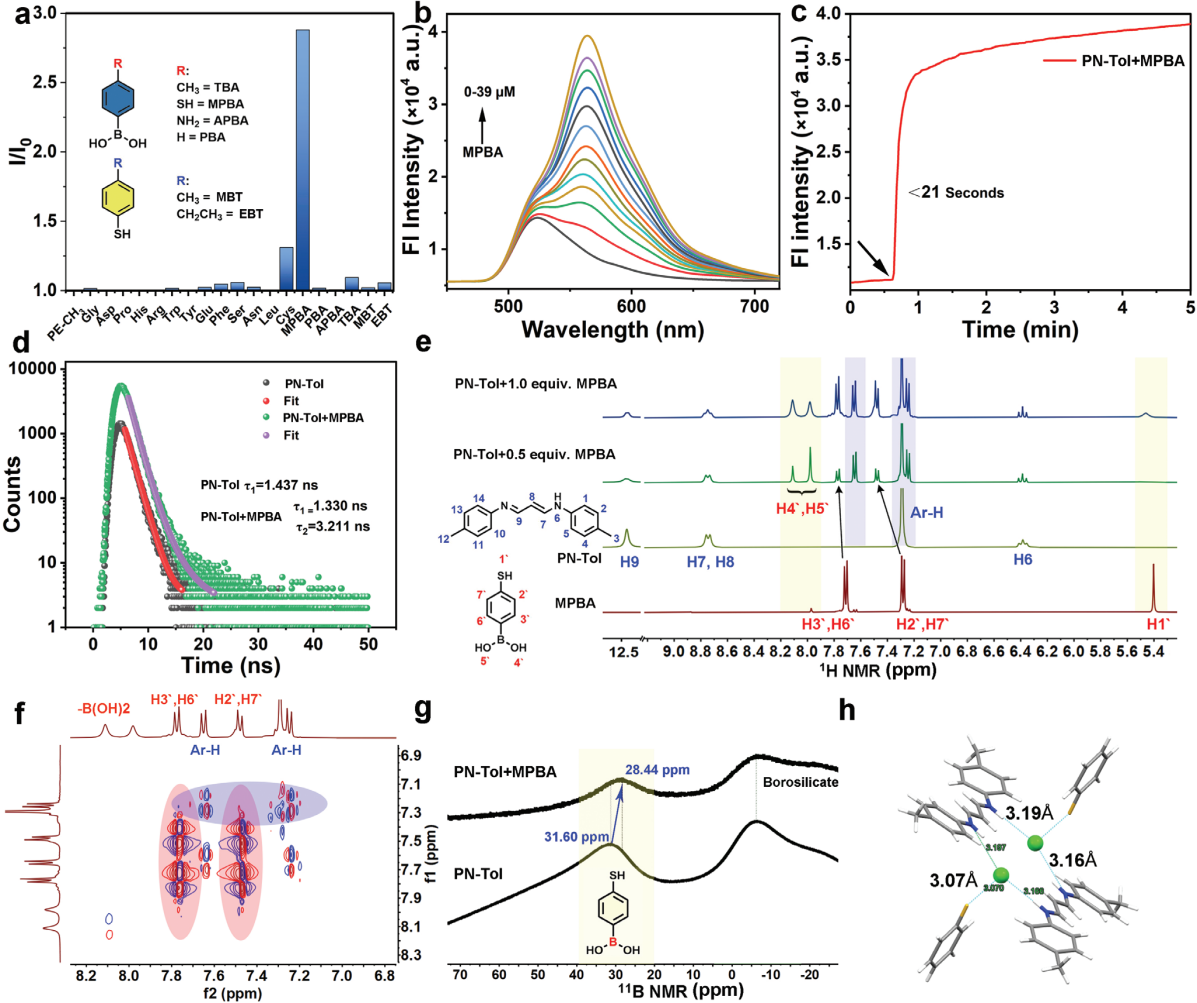
Selective binding of PN‐Tol to MPBA. a) Relative fluorescent intensity (I/I_0_) of PN‐Tol (33 µm) with addition of different amino acids or MPBA analogs (0.3 mm) at 25 °C, Ex: 293 nm; b) Fluorescence spectra after addition of different concentrations of MPBA (0–39 µm); c) Kinetic curve of fluorescence intensity changes (at 565 nm) of PN‐Tol (33 µm) after addition of MPBA (66 µm). To slow down the complexation process, the slowest stirring rate of 30 rounds per minute was set in this test; d) Fluorescence decay curves and corresponding lifetime values of PN‐Tol (0.2 mm) and its mixture with MPBA, detected by time‐resolved spectroscopy. For these fluorescence tests, Tris‐HCl buffers (10 mm, pH 6.8) are used as the solutions and the test temperature is 25 °C. e) Hydrogen nuclear magnetic resonance (^1^H NMR) spectra of PN‐Tol (40 mg·mL^−1^), MPBA (40 mg mL^−1^), and PN‐Tol after adding 0.5 equiv. and 1 equiv. MPBA in *d_6_*‐DSMO at 25 °C, respectively; f) 2D COSY ^1^H–^1^H spectrum of PN‐Tol mixed with equimolar MPBA (0.4 M) in *d_6_*‐DSMO at 25 °C; g)^11^B NMR spectra of PN‐Tol and its mixture with equimolar MPBA (0.6 m) in *d_6_*‐DSMO; h) The crystal structure and binding mode of the complex formed by PN‐Tol and thiophenol, where green represents chlorine atoms and yellow represents sulfur atoms.

Next, we attempted to apply this AIE probe in molecular recognition by investigating a range of amino acids as target analytes. The results revealed that PN‐Tol exhibited negligible fluorescent responses to most amino acids, including glycine (Gly), aspartic acid (Asp), proline (Pro), histidine (His), arginine (Arg), tryptophan (Trp), tyrosine (Try), glutamic acid (Glu), phenylalanine (Phe), serine (Ser), and leucine (Leu), leaving only a weak response observed for Cys (**Figure** **3**a), which was attributed to nucleophilic addition reactions between the ‐SH groups and unsaturated double bonds.^[^ ^36^ ^]^ Surprisingly, the introduction of a boronic acid compound, MPBA, into the PN‐Tol solution caused a significant enhancement (252%) in fluorescence, accompanied by a distinct red shift from 525 to 563 nm. Meanwhile, the fluorescence quantum yield increased from 35.9% to 42.9% (Table S2, Supporting Information). In contrast, the additions of other boronic acid derivatives, such as 4‐aminophenyl boronic acid (APBA), *p*‐tolylboronic acid (TBA), and phenyl boric acid (PBA) did not induce any observable changes in the fluorescence (Figure 3a).

These findings indicated the distinctive attribute of the MPBA structure, which enabled its precise integration into the lattice of PN‐Tol, further restricting the molecular movement and enhancing the AIE effect. In addition, the binding of PN‐Tol to MPBA was concentration‐dependent, as evidenced by the gradual increase in the fluorescent intensity of PN‐Tol with higher concentrations of MPBA (Figure 3b). Time‐dependent changes in fluorescence intensity (at 565 nm) revealed a rapid kinetic process of PN‐Tol in response to MPBA. As shown in Figure 3c, the addition of MPBA leads to a rapid increase in the fluorescent intensity of PN‐Tol. After surpassing 21 s, the growth rate decelerated, eventually reaching equilibrium after ≈4 min. To gain further insights into the changes in the fluorescence mechanism resulting from the binding of PN‐Tol to MPBA, TRF measurements were performed, as shown in Figure 3d. In a Tris‐HCl buffer solution (10 mm), PN‐Tol exhibited a single exponential fluorescence decay with a τ value of 1.43 ns. However, upon binding to MPBA, the fluorescence displayed a double exponential decay with τ values of 1.33 and 3.21 ns. The appearance of a new decay signal with a larger τ value indicated the formation of the PN‐Tol@MPBA complex, which was favorable for AIE emission. Meanwhile, PN‐Tol@MPBA also demonstrated excellent stability. After 731 continuous fluorescence scans over 12 h, the fluorescence intensity values remained near the initial levels. Dynamic light scattering (DLS) tests on PN‐Tol@MPBA standing for 30 and 60 min showed virtually no change in particle size distribution (Figure S4, Supporting Information).

Subsequently, the binding of PN‐Tol to MPBA was confirmed through nuclear magnetic resonance (NMR) titration experiments. Initially, the NMR signals of isolated PN‐Tol and MPBA were studied. As shown in Figure 3e, the proton peaks of PN‐Tol were located at chemical shifts of 12.42, 8.76, 7.29, and 6.38 ppm, corresponding to the hydrogen signals of ‐NH‐, ─CH═CH─, ─Ar─H, and ─CH═N─, respectively. These assignments were verified through 2D NMR (^1^H─^1^H COSY and ^13^C─^1^H HSQC spectra, Figures S5–S7, Supporting Information). After binding MPBA, the new split of signals at 7.66 and 7.25 ppm was observed, and 2D NMR analysis confirmed that these signals belonged to the hydrogen proton in the aromatic region of PN‐Tol (Figure 3f). Meanwhile, the NMR signals of MPBA also experienced significant changes, the proton peak attributed to ─SH (5.40 ppm) disappeared, while the proton peaks at 7.29 and 7.72 ppm underwent a downfield shift (Figure 3e). Moreover, a group of new peaks appeared at 7.98 and 8.11 ppm, and 2D ^1^H–^1^H COSY spectra revealed that they belong to active hydrogen protons of the boronic acid (Figures S8–S10, Supporting Information). This finding also indicated that the hydroxyl groups in boronic acid did not directly participate in the binding between PN‐Tol and MPBA, thereby providing potential binding sites for subsequent saccharide recognition.

With the addition of PN‐Tol, ^11^B NMR of MPBA also exhibits a noticeable high‐field shift from 31.60 to 28.44 ppm (Figure 3g), which could be attributed to the formation of four‐coordinate B‐N coordination bonds.^[^ ^37^ ^]^ The infrared spectrum of the PN‐Tol@MPBA complex shows distinct stretching vibrations at 1036 cm^−1^, attributed to characteristic peaks of the B‐N bond, and a broad peak at 1509 cm^−1^ corresponding to the B‐N‐X bond (where X = halogen) (Figure S11, Supporting Information).^[^ ^38^ ^]^ This further confirms the formation of B‐N coordination bonds. These observations suggest that MPBA functions as a bridge connecting two PN‐Tol molecules. The closer proximity of two molecules enhances shielding effects, leading to the splitting and downfield shift of hydrogen proton signals in the aromatic region. To validate this presumption, ^1^H NMR titration experiments were also conducted employing *p*‐tolylboronic acid (TBA, Figures S12 and S13, Supporting Information) and 4‐methylbenzenethiol (MBT, Figure S14, Supporting Information) as guest molecules. Clear changes in the chemical shift further confirmed the involvement of ─SH and B atoms in the binding between PN‐Tol and MPBA. It is worth noting that the addition of neither TBA nor MBT could cause a significant change in the fluorescence of PN‐Tol, indicating that ─SH and B atoms of MPBA simultaneously participate in the binding with PN‐Tol. The bridging effect of MPBA (Figure 3h) is necessary for the co‐assembly, further highlighting the uniqueness of the MPBA structure. Furthermore, to ascertain the exact binding site of the ‐SH group with PN‐Tol, we performed crystallization studies. Utilizing thiophenol as the core structure for MPBA, the crystal of the complex was successfully obtained. The crystallographic analysis revealed that the ‐SH group binds to MPBA via shared chlorine ions, with an S‐Cl bond length of 3.07 Å. Additionally, the original C─H…Cl hydrogen bonds present in the PN‐Tol dimer were transformed into N‐Cl bonds, with lengths of 3.16 and 3.19 Å. These findings indicate a significant alteration in the spatial structure of PN‐Tol.

### 2.4 Self‐Assembled Morphology Research

**Figure 4:**
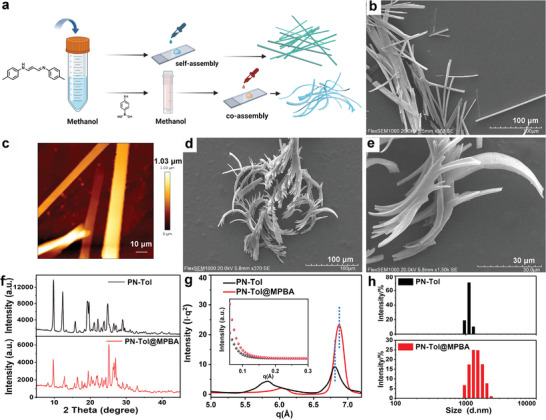
Self‐assembly morphology study. a) Schematic diagram of the self‐assembly process of PN‐Tol and its complex with MPBA; b,d,e) SEM images of the assemblies formed by PN‐Tol (b) or its complex with equimolar MPBA (d, e) on the silicon wafer surface, c) Atomic force microscopy imaging of the assemblies formed by PN‐Tol on mica sheet surface; the concentrations of the PN‐Tol solutions are 2.5 mM in CH_3_OH contain 10% DMSO, test temperature: 25 °C; f) Powder X‐ray diffraction pattern of PN‐Tol (black) and its complex with MPBA (red), scan speed: 5^o^min^−1^; scan range: 2.5–50^o^; g) Structure factors of PN‐Tol (black) and it's complex with MPBA (red) calculated from experimental SAXS data, inset shows the corresponding SAXS scattering curves; h) Particle size distribution of PN‐Tol (upper panel) and it's complex with MPBA (lower panel) in water (1 mm), measured by dynamic light scattering at 25 °C.

During the preparation process, PN‐Tol exhibited a remarkable tendency to crystallize from the solution, forming regular rectangular structures. These crystals displayed strong fluorescence (Figure 2a inset). The absolute fluorescence quantum yield of the crystals was measured at 81.3%, significantly higher than that of the powder and solution forms, indicating that PN‐Tol possesses crystallization‐induced emission enhancement properties (Figure S15, Supporting Information).^[^ ^39^ ^]^ This crystallization phenomenon prompted us to conduct an in‐depth investigation. To begin, one droplet of a CH_3_OH solution of PN‐Tol was deposited onto a silicon wafer and allowed to slowly evaporate at room temperature (**Figure** **4**a). Subsequent scanning electron microscopy (SEM) imaging revealed well‐ordered rod‐shaped assemblies on the wafer surface (Figure 4b). Atomic force microscopy (AFM) imaging provided further details, showing that these assemblies had an average height of ≈1 µm and a width of ≈9 µm (Figure 4c). Notably, regardless of the solvent type or sample concentration, PN‐Tol consistently formed ordered rod‐like structures (Figures S16–S18, Supporting Information). The variation in sample concentration only affected the size of the assemblies, confirming the exceptional self‐assembly capabilities of PN‐Tol. Notably, when Cl^−^ in PN‐Tol was replaced by F^−^, Br^−^, BF_4_^−^, or *p*‐toluenesulfonate ions, the molecules continued to form ordered assemblies. The resulting morphologies differ significantly from PN‐Tol, giving rise to structures that are sheet‐like, block‐like, or even petal‐like in appearance (Figure S19, Supporting Information).

In light of the selective binding ability of PN‐Tol to MPBA, we further investigated the self‐assembly behavior in the presence of equimolar MPBA. The addition of MPBA did not disrupt the self‐assembly of PN‐Tol; instead, it resulted in the formation of co‐assemblies (Figure 4d; Figure S20, Supporting Information), exhibiting a twisted rod‐like structure with an average width of 5.6 µm (Figure 4e; Figure S21, Supporting Information). This indicated that MPBA could influence the self‐assembly process of PN‐Tol. For comparison, the control experiment revealed that individual MPBA molecules did not engage in any assembly behavior (Figure S22, Supporting Information).

X‐ray diffraction (XRD) test further revealed the ordered arrangement of PN‐Tol molecules. The XRD spectrum displays many sharp and intense signal peaks (Figure 4f). Specifically, the characteristic diffraction peaks were located at 2θ values of 9.89°, 12.43°, 19.20°, and 24.89°, which indicated the presence of highly ordered microcrystalline structures. When PN‐Tol was mixed with equimolar MPBA, most of the characteristic diffraction peaks were maintained, differently, the overall signal intensity decreased. Particularly, the diffraction peaks at 9.89°, 12.43°, and 19.20° reduced by 68.7%, 77.9%, and 65.7%, respectively. In contrast, the signal peak at 27.06° increased, and two new peaks emerged at 26.39° and 26.68°. This suggested a significant change in the steric packing mode of PN‐Tol upon binding with MPBA, corroborating the observations from the SEM test.

Small‐angle X‐ray scattering (SAXS) also confirmed the changes in the crystalline structure after co‐assembly. To identify the location of the scattering peak more clearly, we multiplied the scattering intensity (l) with the square of q and plotted it as Iq^2^ versus q.^[^ ^40^ ^]^ As shown in Figure 4g, PN‐Tol exhibits characteristic scattering peaks at 5.83 and 6.81 Å. When combined with MPBA, the intensity of the peak at 5.83 Å weakens and shifts to 6.07 Å, while the peak at 6.81 Å shifts to 6.87 Å, indicating the spatial arrangement of PA‐CH_3_ has changed significantly after the co‐assembly. According to the Bragg expression (L = 2π/q_max_),^[^ ^41^ ^]^ the average distance between adjacent crystal faces of PN‐Tol was calculated to be 0.922 nm, whereas it reduced to 0.914 nm in its co‐assembly with MPBA. This reduction indicated a more compact crystal packing of PN‐Tol after binding with MPBA, providing further insight into the enhanced AIE effect. DLS tests provided additional evidence. When tested in H_2_O, PN‐Tol particles displayed a uniform size of ≈1106 nm (Figure 4h). However, after the addition of MPBA, the average particle size increased to ≈1483 nm, accompanied by a broader size distribution ranging from 955 to 2304 nm.

### 2.5 Selective Response to Saccharides

**Figure 5:**
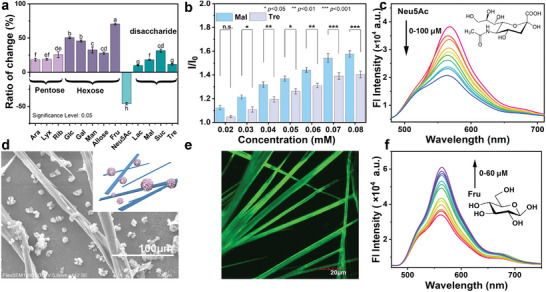
Different fluorescent responses to saccharides. a) The ratio of change in fluorescent intensity (at 565 nm) of PN‐Tol@MPBA (33 µM) after adding various saccharides (66 µm); All values are shown as mean ± SE, and significance analysis was calculated by the origin 2021 software. Lowercase letters indicate the significant statistical differences by one‐way ANOVA with the Tukey test (*P* < 0.05). There is no significant difference when the mark contains the same letter [a, b, c, d, e, f, g, and h (*P* < 0.05)]. b) Fluorescent intensity changes (at 565 nm) of PN‐Tol@MPBA (33 µM) upon addition of different concentrations of Mal (blue) and Tre (purple), (n.s. not significant, ^*^ *P* < 0.05, ^**^ *P* < 0.01, ^***^ *P* < 0.001, versus Mal/Tre group); c,f) Fluorescence emission spectra of PN‐Tol@MPBA (33 µm) after adding different concentrations of Neu5Ac (c) or Fru (f), respectively; d, e) SEM image, schematic diagram (inset), and LCSM image (e) of the co‐assemblies of PN‐Tol@MPBA (5.0 mm in CH_3_OH solution containing 10% DMSO) with equimolar Neu5Ac on silicon wafer surface; For these fluorescent tests, Tris‐HCl buffers (10 mm, pH 6.8) are used as the solutions and the test temperature is 25 °C.

Furthermore, we aimed to utilize the PN‐Tol@MPBA co‐assembly as a probe for saccharide recognition. This thought stems from the remarkable affinity of phenylboronic acid for *cis*‐diol compounds,^[^ ^42^ ^]^ facilitating the discernment of polyhydroxylated compounds through the dynamic formation of B─O bonds.^[^ ^43^ ^]^ When various saccharides were added into the co‐assembly solutions, pronounced changes in fluorescence were detected, however, the ratio of fluorescence change varied among different saccharides (**Figure** **5**a). For example, remarkable fluorescent enhancements were detected for D‐fructose (D‐Fru), glucose (Glc), galactose (Gal), mannose (Man), and allose, with respective percentage changes of 70%, 50%, 46%, 34%, and 28%. This differential fluorescence response has sparked considerable interest, particularly considering the minimal structural differences between these mono‐saccharides. For instance, both Gal and Man are epimers of Glc, sharing identical structural formulas but differing only in the orientation of hydroxyl groups. Furthermore, it is noteworthy that maltose (Mal) and trehalose (Tre), which differ only in the glycosidic linkage (α(1→4) and α(1→1) for Mal and Tre), exhibit distinct fluorescence responses, even at different concentrations (Figure 5b). These results suggested that the co‐assembled probe, unlike conventional synthetic receptors, possesses a remarkably robust ability to recognize various saccharides. The detailed recognition mechanism will be discussed in subsequent investigations.

Moreover, we also observed an intriguing phenomenon related to *N*‐acetylneuraminic acid (Neu5Ac), a typical sialic acid, where the probe exhibited a distinct fluorescence response. With the increase in Neu5Ac concentration, the fluorescent intensity of PN‐Tol@MPBA gradually decreased from 41 004 to 21 878 au (Figure 5c). These data indicated that PN‐Tol could be applied for the recognition of Neu5Ac. It is noteworthy that Neu5Ac is a highly significant nonulosonic acid,^[^ ^44^ ^]^ and its abnormal expression and distribution on the cell surface or in body fluids have been proven to be closely associated with various diseases,^[^ ^45^ ^]^ including cancer, diabetes, cardiovascular, and nervous system diseases. Therefore, the development of a probe capable of selectively detecting Neu5Ac holds substantial application value.

This intriguing phenomenon prompted us to explore its underlying reasons. We speculate that different configurations of *cis*‐1,2‐ or 1,3‐diol exist in saccharide,^[^ ^46^ ^]^ leading to variances in affinity and thereby influencing the AIE behavior of PN‐Tol@MPBA. To investigate this, an SEM imaging analysis was conducted. Initially, we observed the self‐assembly of PN‐Tol in the presence of Neu5Ac. As shown in Figure 5d, numerous rod‐shaped and spherical nanostructures could be observed. The rod‐shaped structures correspond to the assemblies of PN‐Tol, while the spherical nanostructures might have formed due to the complexation of Neu5Ac and MPBA. This assumption was confirmed by laser confocal scanning microscope (LCSM) imaging, where only rod‐shaped assemblies were observed and emitted bright green fluorescence (Figure 5e). The aforementioned spherical nanostructures did not emit fluorescence, indicating the absence of PN‐Tol. Furthermore, mass spectrometry results revealed the formation of complexes between Neu5Ac and MPBA, confirming our speculation (Figure S23, Supporting Information).

Furthermore, taking Fru as an example, we conducted an in‐depth study on the enhanced fluorescence response of PN‐Tol@MPBA to other saccharides. As shown in Figure 5f, the fluorescence of the complex gradually increased with the concentration of Fru and reached equilibrium when 1.8 equivalents of D‐Fru were added. And, the SAXS results reveal that the interlayer spacing of the assembly formed by PN‐Tol@MPBA and Fru is 0.90 nm, a reduction of 0.14 nm compared to PN‐Tol@MPBA alone (Figure S24, Supporting Information). This indicates the formation of a ternary co‐assembly, resulting in tighter molecular packing and further constraining the internal motion of PN‐Tol molecules, thereby enhancing the AIE effect. Based on these findings, we presumed that a competitive replacement reaction occurred in the solution. Neu5Ac captured the MPBA in the PN‐Tol@MPBA co‐assemblies, resulting in the release of PN‐Tol, which diminished the fluorescence. This competitive replacement mechanism has also been mentioned in previous reports.^[^ ^47^ ^]^ On the contrary, when other saccharides bound to PN‐Tol@MPBA, ternary co‐assemblies were generated, resulting in enhanced AIE.

### 2.6 Diverse Ternary Assembled Behaviors with Various Saccharides

**Figure 6:**
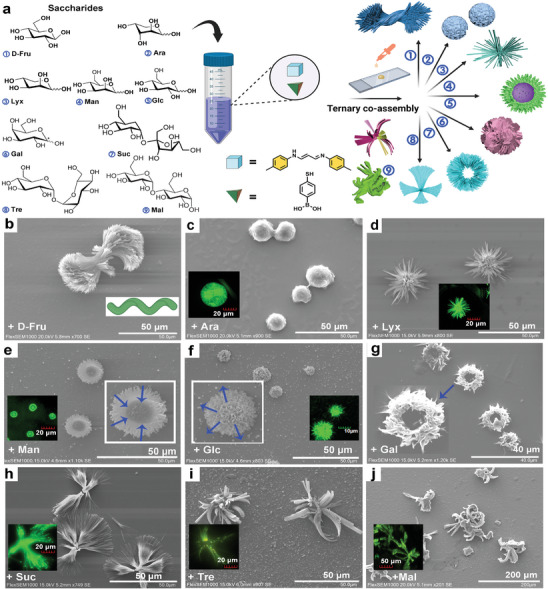
Self‐assembly morphology of PN‐Tol@MPBA with various saccharides. a) Schematic diagram of the ternary co‐assembly process and the typical characteristic of each assembly. b–j) SEM images of ordered assemblies on silicon surface formed by PN‐Tol@MPBA with D‐Fru (b), Ara (c), Lyx (d), Man (e), Glc (f), Gal (g), Suc (h), Tre (i) and Mal (i), respectively. The inset of b represents the twisting direction of the spiral. The concentrations of these saccharides are 2.5 mm, HPLC grade CH_3_OH was used as the solvent contains 10% DMSO, and the temperature is 25 °C. Here Tris‐HCl buffer was not used because salts could crystallize on the surface, which strongly impacts on observation of the co‐assemblies. The inset of (c–f,h–j) represents the LCSM image of the corresponding assembly in quartz plate surface (2.5 mm in CH_3_OH contains 10% DMSO), Ex: 405 nm.

Subsequently, SEM imaging was employed to confirm the ternary assembly behaviors of PN‐Tol@MPBA in the presence of various saccharides (**Figure** **6**a). Interestingly, a diverse range of ordered assembly structures was observed, indicating the successful formation of ternary co‐assemblies between PN‐Tol@MPBA and different saccharides. This phenomenon was further confirmed by XRD tests (Figure S25, Supporting Information). The XRD scans of the ternary co‐assemblies formed with different saccharides exhibited distinct characteristic peaks, indicating the highly ordered nature of these co‐assemblies.

For instance, PN‐Tol@MPBA and D‐Fru formed twisted microclusters (Figure 6b), consisting of multiple micro‐rods with lengths ranging from 56.1 to 94.6 µm and a maximum width of 46.8 µm. Particularly, a left‐handed helical structure was formed in the center of the micro rods, displaying a typical chiral assembly with a centrally symmetric stacking pattern. This intriguing phenomenon suggests that PN‐Tol@MPBA and D‐Fru can form helical assemblies. These helical structures can form extensively and maintain excellent uniformity over large areas (Figure S26, Supporting Information).

Furthermore, when we studied the assembled morphologies formed by PN‐Tol@MPBA and pentoses (e.g., Arabinose (Ara) and Lyxose (Lyx)), they exhibited striking differences. For instance, PN‐Tol@MPBA in the presence of Ara, forms spherical microstructures with an approximate diameter of 13.8 µm (Figure 6c; Figure S27, Supporting Information). On the other hand, in the case of Lyx, PN‐Tol@MPBA formed uniform clusters, characterized by the stacking of hundreds of nanosheets, AFM imaging reveals a cluster size of ≈17.1 µm (Figure 6d; Figures S28 and S29, Supporting Information). This finding is remarkable because Ara and Lxy have highly similar structures, being stereoisomers with the only difference lying in the orientation of their hydroxyl group. Similarly, when hexoses (e.g., Man, Glc and Gal) were involved, PN‐Tol@MPBA formed circular microstructures with diameters of 16.5 and 9.1 µm, respectively. Differently, the assemblies formed with Man exhibit a central depression (Figure 6e; Figure S30, Supporting Information), while those formed with Glc display a central protrusion and a height of 539 nm (Figure 6f; Figure S31, Supporting Information). At the same time, LCSM imaging indicates that these assemblies can also emit bright fluorescence under excitation light (ex: 405 nm), further demonstrating the uniqueness of this ternary assembly (Figure 6c–f,h–j inset). For Gal, the assemblies formed ring‐like structures, as evidenced by the magnified images showing each ring composed of a series of intertwined nanobelts. Energy dispersive spectrometer analysis shows that this microring contains abundant elements C, O, and S (Figure 6h; Figure S32, Supporting Information).

The diverse morphologies of these ternary assemblies may result from various binding modes of PN‐Tol@MPBA with different saccharides.^[^ ^48^ ^]^ PN‐Tol@MPBA forms cyclic five‐ or six‐membered boronate esters with the *cis*‐1,2 or *cis*‐1,3 diols of saccharides.^[^ ^49^ ^]^ These boronate esters are stabilized by hydrogen bonds between the hydroxyl groups of PN‐Tol@MPBA and saccharide molecules. The stereo configuration of saccharides leads to diverse spatial orientations of *cis*‐diols, resulting in the formation of different aggregates. Interactions at the substrate interface during solvent evaporation contribute to the formation of ordered assemblies with specific morphologies. For instance, PN‐Tol@MPBA aggregates with Gal through hydrogen bonding and boron‐oxygen bond interactions in solution. Upon deposition onto a substrate, solvent evaporation promotes the fusion of these aggregates into nanoscale rod‐like structures. Gradually, controlled interfacial tension then induces the curling of these nanorods, eventually forming microring.^[^ ^50^ ^]^

In response to disaccharides, PN‐Tol@MPBA can form larger microstructures. For instance, when interacting with Suc, PN‐Tol@MPBA forms fan‐shaped structures (Figure 6h; Figure S33, Supporting Information). In contrast, with the disaccharide Tre, formed by two glucose molecules, PN‐Tol@MPBA forms flower‐like structures (Figure 6i; Figure S34, Supporting Information). Meanwhile, its constitutional isomer, Mal, gives rise to curved block‐like structures (Figure 6j; Figure S35, Supporting Information). On the one hand, these results indicated that the enantiomers and constitutional isomers of saccharides, which are difficult to differentiate using conventional methods, could be precisely distinguished through the self‐assembled morphologies at the micrometer scale. On the other hand, PN‐Tol, as an ideal self‐assembling module, demonstrates its excellent ability to generate various 3D nano‐ and micro‐structures with the participation of various saccharides, which endows diversity and controllability to molecular self‐assembly. This clever molecular building block method has the potential to become a powerful tool for constructing various complex structures in the future. Consequently, it is anticipated that PN‐Tol will gain widespread recognition, and its value in self‐assembly endeavors will continue to be explored and expanded.

### 2.7 Discriminating Saccharides with Array Sensor

Having established the capability of PN‐Tol@MPBA to exhibit differential fluorescence responses toward various saccharides, our next objective was to develop an array sensor capable of simultaneously differentiating and classifying these saccharides. The concept of an array sensor, initially proposed by Professor Anslyn and colleagues,^[^ ^51^ ^]^ has proven highly effective in sensing differences in various biologically significant species.^[^ ^52^ ^]^ This method involves using a set of receptors with appropriate selectivity to respond to a variety of analytes. The obtained signals are then processed through multivariate statistical analysis, such as Principal Component Analysis (PCA), to provide a unique “fingerprint” for each analyte.^[^ ^53^ ^]^

**Figure 7:**
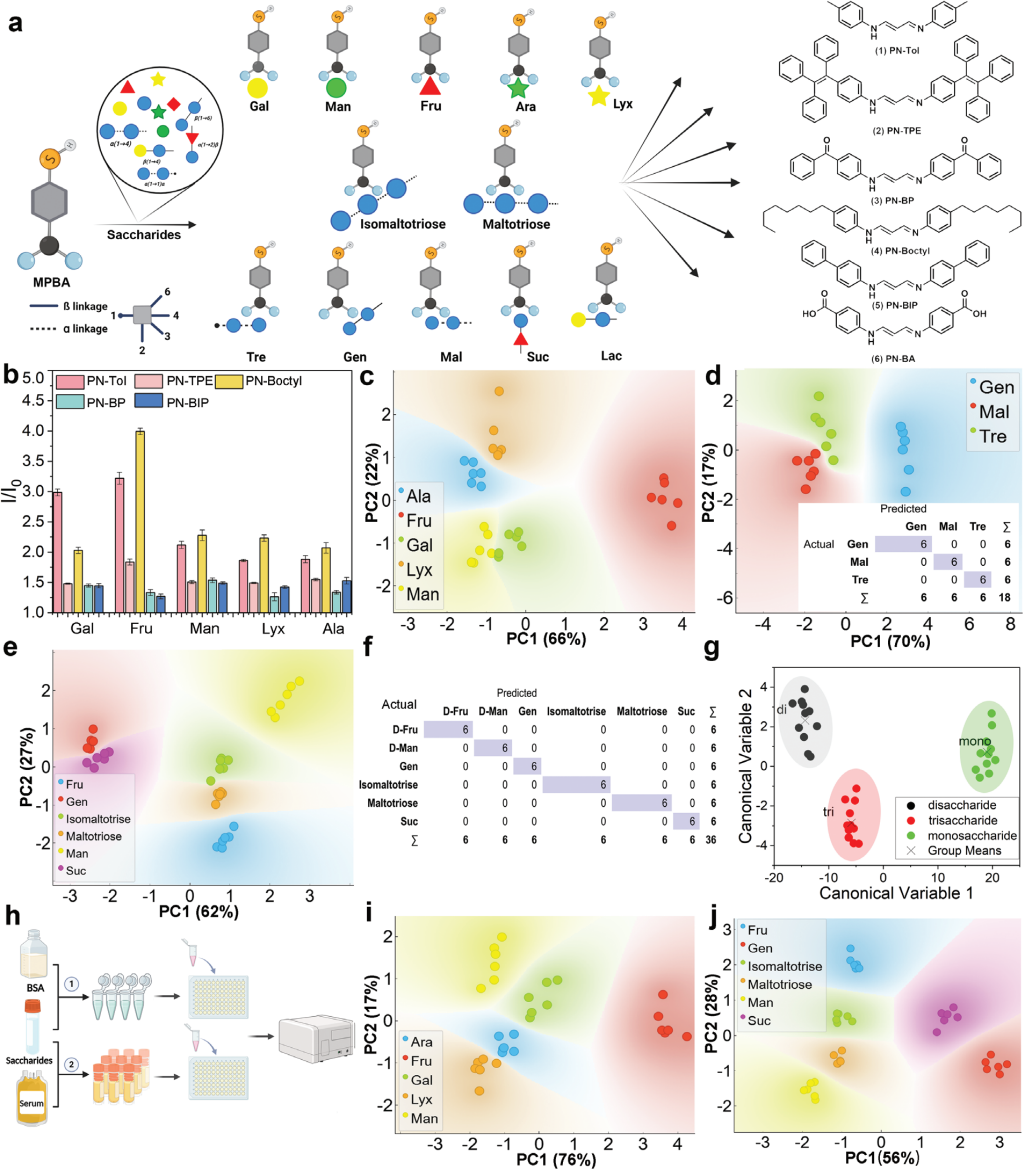
Fluorescent array sensors and data analysis. a) Schematic diagram of the array sensor; b) Fluorescence responses (*I*) upon addition of Ala, Lyx, Fru, Gal, and Man to the array sensor, *I_0_* represents the initial fluorescent intensity. Each component in the array displays different fluorescence responses, enabling multivariate analysis for distinguishing the saccharide categories. c) PCA scores plot generated from the data in (b). d) PCA scores plot of Gen, Mal, Tre, using PN‐Tol, PN‐TPE, PN‐Boctyl, PN‐BP, and PN‐BIP as the optimal array combination. The inset shows the confusion matrix of the PCA analysis. Correct predictions are represented by on‐diagonal blue squares, while incorrect predictions are represented by off‐diagonal white squares. e) PCA scores plot of mono‐, di‐, and tri‐saccharide, using PN‐Tol, PN‐TPE, PN‐Boctyl, PN‐BP, and PN‐BA as the optimal array combination. Error bars in (b) indicate the standard deviation of six repeated measurements. f) Confusion matrix of the PCA analysis of mono‐, di‐, and tri‐saccharides. g) Canonical scores plot resulting from CDA of the individual responses from the five dyes to the 36 samples grouped by category. The test solution was Tris‐HCl buffer (10 mm, pH 6.8), with a volume of 200 µL containing PN‐Tol (2.5 µm), MPBA, and saccharides (5.0 µm). The testing temperature was 27 °C. Ex: 385 nm, Em: 565 nm. h) Schematic diagram of the array sensor, executed in a mixed sample; i) PCA scores plot of Ala, Lyx, Fru, Gal, and Man (5.0 µm) in BSA mixture (0.31 mg mL^−1^ in Tris‐HCl buffer (10 mm, pH 6.8)); j) PCA scores plot of Fru, Gen, Maltotriose, Isomaltotriose, Man, Suc (5.0 µm) in serum mixture with a total protein content of 1.53 mg mL^−1^.

In this study, we introduced six different PN‐based dyes (i.e., PN‐Tol, PN‐Boctyl, PN‐TPE, PN‐BP, PN‐BIP, and PN‐BA) to build the array for the differentiation of saccharides (**Figure** **7**a) and fluorescence titration experiments revealed that all six compounds can form assemblies with MPBA (Figures S36 and S37, Supporting Information). Initially, the saccharides were mixed with equimolar MPBA, and then added to each dye solution (2.5 µm in Tris‐HCl buffer (10 mm, pH 6.8)) in a 96‐well plate, maintaining a fixed concentration of 5.0 µm. The fluorescent intensity (at 565 nm) was then measured for each well by a microplate reader. Figure 7b displays the response plot obtained when the array was exposed to five different mono‐saccharide targets (i.e., Gal, Fru, Man, Lyx, and Ala), illustrating highly variable responses. Subsequently, we performed PCA analysis on the array readouts (across 6 separate runs), generating a scores plot as shown in Figure 7c. The results demonstrated that the array could effectively discriminate all five mono‐saccharides. Notably, in addition to distinguishing between pentose (Ala, Lyx) and hexose (Fru, Gal, Man), the array could discriminate saccharide isomers. This indicated that the array was highly sensitive to subtle changes in the orientation of the hydroxyl.

Furthermore, we successfully utilized the array to distinguish di‐saccharides, such as Lac, Suc, and Mal (Figures S38 and S39, Supporting Information). However, a more challenging task was distinguishing di‐saccharides with the same composition but different glycosidic linkages, such as Mal, Tre, and Gentiobiose (Gen). Although all three consist of two Glc monomers, their glycosidic linkage are α(1→4), α(1→1)α, and β(1→4), respectively, representing one of the most challenging targets in saccharide recognition. Nevertheless, the PCA results showed that the array could clearly discriminate these three di‐saccharides with different linkage types (Figure 7d; Figure S40, Supporting Information), illustrating the powerful advantage of the array sensor in saccharide recognition. In addition, the validation accuracy of the tests was determined by employing a linear discriminant analysis (LDA) model to measure the correct and incorrect classifications of samples. We visually represented the classifications in a confusion matrix (Figure 7d inset), where the rows represent the true classes and the columns represent the predicted classes. The PCA achieved 100% validation accuracy, as determined by comparing correctly classified samples (diagonals, blue) versus misclassified samples (off diagonals, white).

A more stringent test was conducted to assess whether the array could simultaneously distinguish structurally similar saccharides and provide accurate classification. For this purpose, we selected three classes consisting of six kinds of saccharides, including two mono‐saccharides (Fru and Man), two di‐saccharides (Gen and Suc), and two tri‐saccharides (Maltotriose and Isomaltotriose), as the target analytes. The explicit result from the PCA plot indicated that the array could easily distinguish these six saccharides, with significant differentiation observed within the different classes. Each class was positioned at three distinct locations on the plot (Figure 7e; Figure S41, Supporting Information). Furthermore, structurally similar saccharides appeared in adjacent positions, indicating that the array could not only distinguish these saccharides but also classify them based on subtle structural differences. Additionally, the PCA analysis using a confusion matrix confirmed the 100% accuracy of saccharide classification based on their compositions (Figure 7f).

To further validate our findings, the responses were also subjected to canonical discriminant analysis (CDA). Unlike PCA, which is an unsupervised classification tool that identifies the greatest variance between samples and clusters the samples with a smaller variance, CDA is a supervised classification method that incorporates class information to maximize discrimination among class ^47^. Thus, the unsupervised PCA confirms the differentiation of saccharide structures, and the supervised CDA determines whether the array can classify the saccharides based on their composition. In this case, the raw data obtained from the five‐dyes array, which included all six repeats for each of the six saccharides (a total of 180 samples), were used as the input, together with their classification information. The results of the CDA analysis are highly impressive: the canonical scores plot (Figure 7g) clearly shows the robust classification of the targets into the three expected groups, mono, di, and tri (shown in green, black, and red, respectively, in Figure 7g). This analysis effectively corroborated the PCA results and validated the array's ability to classify highly similar saccharides based on their composition.

Finally, to assess the efficacy of the array in saccharide recognition within complex samples, we conducted tests in a bovine serum albumin (BSA) mixture (with a concentration of 0.31 mg mL^−1^) (Figure 7h). The PCA plot demonstrates that the array can still effectively discriminate various saccharides and their isomers (Figure 7i). As a further investigation, various saccharides were introduced into serum, a complex bio‐sample with a high total protein content of 1.53 mg mL^−1^, which was ≈130‐fold higher than the tested saccharides, and subjected to batch saccharide analysis using the array sensor. The results demonstrate that under these conditions, the array maintains robust reproducibility of sensing (Figure S42, Supporting Information), achieving 100% identification accuracy for 6 saccharides with no overlap observed in the PCA plot (Figures S43 and S44, Supporting Information). Additionally, the capability of the array to distinguish mono‐saccharides (Fru and Man), di‐saccharides, (Gen and Suc), and tri‐saccharides (Maltotriose and Isomaltotriose) in serum mixture was validated, with PCA results indicating successful differentiation among these saccharides (Figure 7j). These results unequivocally demonstrate that the array's sensing and differentiation capabilities for saccharides remain unimpaired even in complex samples. Moreover, it sheds additional light on the unparalleled superiority and potent efficacy of this ternary co‐assembly strategy in saccharide recognition.

## 3 Conclusion

In summary, saccharide recognition plays a crucial role in biological processes, influencing various diseases like diabetes, viral infections, immune responses, autoimmune disorders, and certain cancers. Our study introduces a novel approach focusing on precise saccharide recognition. Leveraging PN‐Tol's inherent ability to fluoresce intensely upon aggregation and its co‐assembly capability with MPBA and saccharides, we've developed a highly efficient ternary co‐assembly system. This innovation streamlines complex molecular synthesis and extraction processes while enhancing specificity and sensitivity, opening new avenues for saccharide receptor design. Meanwhile, ternary co‐assembly offers opportunities for creating intricate and precise architectures that integrate multiple functionalities within a single assembly. However, achieving successful ternary co‐assembly involves balancing multiple interactions, demanding precise control and advanced techniques. This work exemplifies successful ternary co‐assembly, providing a swift method for screening assembly components and optimizing module combinations using the potent AIE effect. This approach accelerates the discovery of ternary co‐assembly systems, advancing molecular assembly from necessity to freedom and inspiring diverse applications with limitless potential.

## Conflict of Interest

The authors declare no conflict of interest.

## Author Contributions

Y.C. and G.Q. conceived the study, designed the experiments, and wrote the manuscripts. Y.C. performed most of the experiments with the help of J.S. and J.L., and X. Z. performed the PCA analysis. Y.D. and W.S. performed spectrum experiments. H.Q. performed the NMR experiment. Q.L. participated in the discussion of the experiment results. G.Q. analyzed all the results with the help of G.W.

## Supporting information

Supporting Information

## Data Availability

The data that support the findings of this study are available from the corresponding author upon reasonable request.
