# Supplementary material for: Precise AIE‐Based Ternary Co‐Assembly for Saccharide Recognition and Classification
Source: Adv Sci (Weinh). 2024 Aug 28;11(40):2405613. doi: 10.1002/advs.202405613 (PMC11633354; doi:10.1002/advs.202405613)
Supplement: Supplementary file 1 — Supporting Information [file ADVS-11-2405613-s001.pdf]

ADVANCED SCIENCE
Open Access

Supporting Information

for *Adv. Sci.*, DOI 10.1002/advs.202405613

Precise AIE-Based Ternary Co-Assembly for Saccharide Recognition and Classification

*Yongxin Chang, Juan Shao, Xinjia Zhao, Haijuan Qin, Yanqing Du, Junrong Li, Qiongya Li, Wenjing Sun, Guoxiong Wang and Guangyan Qing**

Supporting Information

Precise AIE-based Ternary Co-assembly for Saccharide Recognition and Classification

Yongxin Chang[1,2], Juan Shao[1], Xinjia Zhao[1], Haijuan Qin[3], Yanqing Du[4], Junrong Li[1], Qiongya Li[1], Wenjing Sun[1], Guoxiong Wang[2] and Guangyan Qing[1]*

[1] State Key Laboratory of Medical Proteomics, National Chromatographic R. & A. Center, CAS Key Laboratory of Separation Science for Analytical Chemistry, Dalian Institute of Chemical Physics, Chinese Academy of Sciences, Dalian 116023, P. R. China

[2] State Key Laboratory of Catalysis, Dalian Institute of Chemical Physics, Chinese Academy of Sciences, Dalian 116023, P. R. China

[3] Research Centre of Modern Analytical Technology, Tianjin University of Science and Technology, Tianjin, 300457, P. R. China

[4] Department of Pharmaceutical Sciences, Inner Mongolia Medical University, Hohhot,010110, P. R. China

*Corresponding author: Email: qinggy@dicp.ac.cn (G.Q.)

Table of contents

1. Supporting Methods 3

1.1 Materials and Instruments 3

1.2 UV–vis and fluorescence spectrum experiments 4

1.2.1 Spectral performance testing of PN-Tol 4

1.2.2 Response to different analyte tests 5

1.3 SEM sample preparation 5

1.4 XRD measurements: 6

1.5 X-ray Crystallography 6

1.6 NMR Test 7

1.7 Particle size measurements 8

1.8 Fluorescence lifetime measurements 8

1.9 Array sensing testing 9

2. Supporting Figures 11

3. Supporting Tables 35

3.1 Table 1. Crystal data and structure refinements for PN-Tol and PN-Tol@thiophenol 35

3.2 Table 2. Spectral parameters of PN-Tol and PN-Tol@MPBA 36

4. Synthetic procedures and characterization data 37

4.1 Synthesis of PN-Tol: 37

4.2 Synthesis of PN-BA: 39

4.3 Synthesis of PN-Boctyl: 41

4.4 Synthesis of PN-BIP: 43

4.5 Synthesis of PN-BP: 45

4.6 Synthesis of PN-TPE: 47

5. References 49

1. Supporting Methods

1.1 Materials and Instruments

Materials. p-Toluidine (99%) was purchased from Shanghai Bide Pharmaceutical Technology Co., Ltd. (P. R. China). 4-Mercaptophenylboronic acid (99%) was purchased from Sigma-Aldrich Co., Ltd. (St. Louis, MO, USA). Tetramethoxypropane (Cas No. 102-52-3, 98%) was purchased from Shanghai Adamas Reagent, Ltd. (P. R. China). d_6-dimethyl sulfoxide (d_6-DMSO, 99.8 atom % D, with 0.03% (v/v) TMS), 4-n-Octylaniline (97%), 4-aminobenzoic acid (98%), 1-(4-aminophenyl)-1,2,2-triphenylethene (Cas No. 919789-80-3, 97%), 4-amino benzophenone (98%), n-Hexane (anhydrous, 99%, water≤50 ppm), 4-aminobiphenyl (98%), Lactose (Lac, 98%), Maltose (Mal, 95%) were purchased from Beijing InnoChem Co., Ltd. (P. R. China). Methanol (CH_3OH), ethanol (CH_3CH_2OH), tetrahydrofuran (THF), and acetonitrile (CH_3CN) were chromatographic pure. Other solvents and reaction reagents include hydrochloric acid (HCl, 37 wt.% solutions in water), ethyl acetate (EtOAc), Dimethyl sulfoxide (≥99.9%, for HS-GC, DMSO), *N*, *N*'-dimethylformamide (DMF) were purchased from Sinopharm Chemical Reagent Co., Ltd. (P. R. China). Tris(hydroxymethyl)methyl aminomethane (Tris, 99%) was purchased from Alfa Aesar Corp. (Tianjin, P. R. China). p-Tolylboronic acid (99%, Contains varying amounts of anhydride, CAS: 5720-05-8), p-Thiocresol (98%) were purchased from Aladdin (Shanghai Aladdin Bio-Chem Technology, P. R. China). Leucine (Leu, 98%), Proline (Pro, 98%), Tryptophan (Trp, 99%), Glycine (Gly, 99%), Serine (Ser, 99%), Threonine (Thr, 99%), Cysteine (Cys, 98%), Tyrosine (Tyr, 99%), Histidine (His, 98%), Glutamic acid (Glu, 97%) were purchased from J&K Scientific, Ltd. (P. R. China). D-Arabinose (D-Ara, 99%), D-Lyxose (D-Lyx, 98%), D-Glucose (Glc, 98%), D-Mannose (D-Man, 98%), L-Fructose (L-Fru, 95%), D-Fructose (D-Fru, 99%), Gentiobiose (96%) were purchased from Tokyo Chemical Industry (TCI) Shanghai Development Co., Ltd. (P. R. China). Isomaltotriose (Cas No. 3371-50-4, 99% for HPLC) and Maltotriose (Cas No. 1109-28-0, 99% for HPLC) were purchased from Shanghai ZZBIO. Co., Ltd (P. R. China). Pure water used in all experiments was purified with a Milli-Q system (Millipore, 82 Milford, MA). All reagents and starting materials were purchased commercially and used

without further purification unless otherwise stated.

Instruments. Hydrogen, carbon, and boron nuclear magnetic resonance (^{1}H, ^{13}C,^{11}B NMR) spectra were obtained using a Bruker AVANCE III 400–MHz or AVANCE III HD 700–MHz spectrometer (Bruker Corp., Germany). Mass spectra (MS) were recorded on an Agilent 6540 Quadrupole Time-of-Flight (Q-TOF) mass spectrometer (Agilent, USA). Absorption spectra were measured on a Perkin–Elmer Lamda-365 UV–vis spectrophotometer (Perkin Elmer, USA). Fluorescence spectra were measured by using a Perkin–Elmer FL–6500 fluorescence spectrometer (PerkinElmer, USA). Absolute quantum yield (QY) was obtained on an Edinburgh Instrument FLS980 Integrating sphere (Edinburgh Instruments, UK). Fluorescence lifetime was recorded using a time-correlated single-photon with an Edinburgh EPL–365 picosecond pulsed diode laser as the excitation source counting (TCSPC) method and collected on an Edinburgh FLS980 (Edinburgh, UK). Particle size analyses were implemented using a Zetasizer Nano ZS90 instrument (Malvern NanoZSP+MPT-2, Malvern, UK). Scanning electron microscopy (SEM) images were carried out on a JEOL JSM−7800 F instrument and the elemental compositions were analyzed by an X−ray energy dispersive spectroscopy (EDS) (X−Max50, Oxford), which is connected with the JSM−7800 F instrument. Powder XRD patterns were collected by a Shimadzu XRD−7000S Powder X−ray diffractometer with 1.5418 Å Cu Kα radiation (scan speed: 5°/min; scan range: 2.5–40°, Shimadzu, China). The small-angle X-ray scattering (SAXS) measurements were carried out at Xeuss 2.0SAXS/WAXS System (Xenocs, France) with an X-ray of λ = 0.154 nm. The sample-to-detector distance was 2494.86 mm, covering a momentum transfer (q) range of 0.003-0.185A! and the exposure time was set as 300 s.

1.2 UV–vis and fluorescence spectrum experiments

1.2.1 Spectral performance testing of PN-Tol

The stock solution of PN-Tol was prepared in THF (2.5 mg·mL^{-1}) and stored at room temperature, UV-vis and fluorescence spectra tests were performed in different solvents. In short, the stock

solution of PN-Tol (10 μL) was added to the 3 mL methanol, ethanol, dimethyl sulfoxide, *N,N*-Dimethylformamide, acetonitrile, and tetrahydrofuran, respectively, then spectral data were recorded after 1 min. The fluorescence experiments were carried out in a PerkinElmer FL-6500 fluorescence spectrometer with a 1 cm path-length quartz cuvette. This study selected THF as a good solvent, whereas hexane was used as a poor solvent. The excitation wavelength was set to 293 nm, and the slit width is (10, 10) nm.

1.2.2 Response to different analyte tests

PN-Tol was dissolved in DMSO to prepare a 10 mmol·L^{-1} stock solution, which was diluted to the desired concentration with a Tris-HCl buffer (0.01 M, pH=6.8) before testing. A series of amino acids and saccharides include: Leucine (Leu), Proline (Pro), Tryptophan (Trp), Glycine (Gly), Serine (Ser), Threonine (Thr), Cysteine (Cys), Tyrosine (Tyr), Histidine (His), Glutamic acid (Glu), D-Arabinose (D-Ara), D-Lyxose (D-Lyx), Glucose (Glc), D-Mannose (D-Man), L-Fructose (L-Fru), D-Fructose (D-Fru), Lactose and Maltose were prepared in ultrapure water (0.1 mol·L^{-1}), respectively. UV-vis and fluorescence experiments were conducted in a Tris-HCl buffer solution (0.01 M, pH=6.8) at 25°C. A stock solution of PN-Tol (10 μL) was added to the buffer solution mentioned above, and spectral data were recorded after 1 minute. The peak intensity at 565 nm was recorded as the initial fluorescence value (I_0). Subsequently, the stock solutions of various amino acids and saccharides (10 μL) were added to the PN-Tol solution, respectively, and the emission spectra and fluorescence intensity (I) at 565 nm were recorded. Fluorescence spectra were recorded from 450 to 800 nm with an excitation wavelength of 293 nm. All parameters remained constant during the data collection for different analytes.

1.3 SEM sample preparation

Firstly, prepare stock solutions of PN-Tol in DMSO at a concentration of 0.025 M, MPBA in DMSO at a concentration of 0.1 M, and various saccharides in CH_3OH at a concentration of 0.1 M. Next, combine 10 μL of the PN-Tol stock solution with 90 μL of methanol, ensuring thorough

mixing. Using a pipette, dispense 2.5 μL of this mixture onto a 1.5 x 1.5 cm silicon wafer. Allow the solvent to evaporate in a constant temperature drying oven set at 25°C. For the co-assembly samples, evaporate a mixed solution of PN-Tol and MPBA (2.5 mM in CH_3OH containing 10% DMSO, with a molar ratio of 1:1) onto the silicon wafer surface at 25°C. To prepare ternary co-assemblies, add 2.5 μL of CH_3OH solutions (0.1 M) of saccharides to the prepared PN-Tol and MPBA mixed solution (2.5 mM in CH_3OH containing 10% DMSO, molar ratio 1:1, total volume 100 μL). Apply one drop of the resulting solution evenly onto a clean silicon wafer surface using a dropper, followed by slow evaporation at 25°C in drying ovens. Specific concentrations of the various ternary co-assemblies are specified in the figure legend. Ensure methanol is HPLC grade to prevent interference from solvent impurities.

1.4 XRD measurements:

Powder XRD patterns were collected by a Shimadzu XRD−7000S Powder X−ray diffractometer with 1.5418 Å Cu Kα radiation (scan speed:5°/min; scan range: 2.5–50°). The preparation of the PN-Tol sample involves dissolving 500 mg of the product in 15 mL of methanol. The solution is left at room temperature for over 64 hours, resulting in the formation of needle-like crystals. The crystallized product is then filtered and evaporated to dryness at room temperature. Similar methods are employed for the preparation of other samples, where solutions of equimolar mixtures of PN-Tol with MPBA and PN-Tol with MPBA, along with various saccharides, are allowed to stand at room temperature for 3-7 days, yielding solid crystals. The collected crystals are subsequently used for XRD testing.

1.5 X-ray Crystallography

One hundred milligrams of PN-Tol were dissolved in 8 mL of ethanol, filtered, and the filtrate was transferred to a 10 mL vial. Crystals were obtained from the evaporation of solvent from ethanol of PN-Tol stored at 25 °C for 2–4 weeks. Suitable single crystals then were selected from the mother liquor and covered with paraffin liquid. Single-crystal X-ray diffraction experiments were

carried out on a Bruker D8 VENTURE diffractometer equipped with a CMOS detector using mirror-monochromated Mo Kα radiation (λ = 0.71073 Å). The APEX-III program was used to determine the unit-cell parameters. The data were integrated with the SAINT program and were corrected for the Lorentz factor and polarization effects. Multiscan absorption corrections were applied using SADABS. The molecular structures were solved by direct methods and refined by the full-matrix least-squares on F2 using the SHELXTL program (version 2014/7). All non-hydrogen atoms were refined anisotropically. Crystal data and the final refinement parameters of the studied molecules are shown in Table S2. The crystallographic data for this Article are available, which can be obtained free of charge from the Cambridge Crystallographic Data Centre via http://www.ccdc.cam.ac.uk.

1.6 NMR Test

^{1}H, ^{13}C nuclear magnetic resonance (NMR) spectra were recorded at 298 K using a Bruker AVANCE III 400 MHz or AVANCE III HD 700 MHz spectrometer (Bruker, Germany), automatically tuned and matched to the correct operating frequencies, the sample concentration is 40 and 60 mg/mL in d_6-DMSO, respectively. TopSpin 3.5 and Mestrenova 14.0.0 were used to apply phase and baseline corrections. Chemical shift values for ^{1}H and ^{13}C NMR are reported in ppm with the solvent resonance as the internal standard (DMSO-d_6, 2.50 ppm for ^{1}H, 39.6 ppm for ^{13}C), and coupling constants are reported in Hz. Abbreviations for multiplicity are as follows: *s*, singlet; *d*, doublet; *t*, triplet; *m*, multiplet; *br*, broad. ^{1}H–^{1}H COSY, ^{1}H–^{13}C HSQC NMR experiments were performed on a Bruker AVANCE III 400 spectrometer, the sample concentration is 40 and 60 mg/mL, respectively. ^{11}B NMR spectra were recorded on a Bruker AVANCE Ⅲ 400 MHz spectrometer, using a 4-mm double resonance HX NMR probe with a spinning rate of 10 kHz and a small flip angle of π/12 pulse, the concentration is 6 M and d_6-DMSO was used as a solvent for dissolving samples. For NMR titration experiments, prepare mixed solutions of PN-Tol and MPBA with different molar ratios (1:0.5 and 1:1 for PN-Tol:MPBA) in d_6-DMSO. Allow complete dissolution before transferring them into the nuclear

magnetic tube.

1.7 Particle size measurements

Dissolve a stock solution (0.01 M in DMSO) of PN-Tol and MPBA in H_2O at a molar ratio of 1:1 to prepare DLS samples, achieving a final concentration of 100 μM. The testing temperature was maintained at 25 °C, and the samples underwent sonication for 1 min prior to measurements. The DLS spectra were recorded on a Zetasizer Nano ZS90 instrument at 25 ˚C with a 488 nm laser. A typical DLS experiment was comprised of at least 3 runs. At least 25 scans were taken in each run, each elapsing for 10 seconds. The velocity of particle movement was used to calculate particle sizes (d_h, hydrodynamic diameter) by applying the two–dimensional Stokes–Einstein equation[1].

$$< x,y >^2 = \frac{K_B T t_s}{3\pi \eta d_h}$$

Where $<x, y>^2$ is the mean squared displacement, K_B is Boltzmann's constant, T is the temperature in Kelvin, t_s is the sampling time, η is the viscosity, and d_h is the hydrodynamic diameter. The data were analyzed by NTA 3.3 software (NanoSight). Each run was subsequently averaged in Origin 2021.

1.8 Fluorescence lifetime measurements

Time-resolved fluorescence decays of PN-Tol (1.5×10^{-4} M) in THF and hexane were conducted on an Edinburgh FLS980 spectrometer with a 1 cm path–length quartz cell. The fluorescence decays were recorded using time-correlated single-photon with the Edinburgh EPLED-365 and EPLED-405 picosecond pulsed diode laser as the excitation sources counting (TCSPC) method and collected on an Edinburgh FLS980, the pulse widths of the excitation sources were 868.9 ps and 56.9 ps. The data analysis used a single–exponential (Eq. 1) or double–exponential function (Eq. 2), performed with a software package provided by the Edinburgh instrument[2].

$$y = \alpha e^{-t/\tau} \qquad (1)$$

$$y = \alpha_1 e^{-t/\tau_1} + \alpha_2 e^{-t/\tau_2} \qquad (2)$$

Where τ is a fluorescence lifetime, α_1 and α_2 represent the fractional amounts of molecules with τ_1

and τ_2, respectively.

Absolute φF and τ can be expressed as follows:

$$\varphi_F = \frac{k_F}{k_F + k_{nr}} = \frac{k_F/k_{nr}}{k_F/k_{nr} + 1} \quad (3)$$

$$\tau = \frac{1}{k_F + k_{nr}} \quad (4)$$

Where k_F is the radiative decay rate constant. k_{nr} is the total nonradiative decay rate constant. When there are two τ values, Eq. 4 turns into the following expression.

$$[\tau] = \frac{1}{k_F + k_{nr}} \quad (5)$$

$$[\tau] = \frac{\alpha_1 \tau_1^2 + \alpha_2 \tau_2^2}{\alpha_1 \tau_1 + \alpha_2 \tau_2} \quad (6)$$

Where $[\tau]$ refers to the average fluorescence lifetime. Based on Eq. 3-6, values of k_F and k_{nr} can be obtained.

1.9 Array sensing testing

Fluorescence measurements of the array were conducted by mixing 4 μL of a mixture of saccharide and MPBA solution (0.01 M, dissolved in a mixed solution of DMSO and ultrapure water, v:v =1:1) with 2 μL of dye (0.01 M, dissolved in DMSO) in a black 96-well plate. The total volume of the solution was 200 μL, including 194 μL of Tris-HCl buffer (10 mM, pH=6.8). The mixed solution was linearly oscillated at 25 ℃ for 10 minutes, and fluorescence signals were recorded on a microplate reader (Biotek, US). Subsequently, the same method was used to record fluorescence data of other dyes, with all parameters remaining constant during the data collection. The specific concentrations of sensor arrays were indicated in the figure legend. The excitation wavelengths for PN-Tol, PN-TPE, PN-Boctyl, PN-BP, PN-BIP, and PN-BA were set to 293 nm, 422 nm, 362 nm, 337 nm, 283 nm, and 249 nm, respectively. The initial fluorescence intensities at the maximum emission wavelengths of the dyes were recorded as I_0. After the addition of MPBA and various saccharides, the fluorescence intensities of dyes at the maximum emission wavelengths were recorded as I. The obtained relative fluorescence intensities (I/I_0) were used as the response signals for array sensing analysis. Each experiment was repeated six times. Finally,

the raw data matrix was processed using PCA and confusion matrix analysis in the orange program or using CDA analysis in Origin 2021 software (Scheme 1).

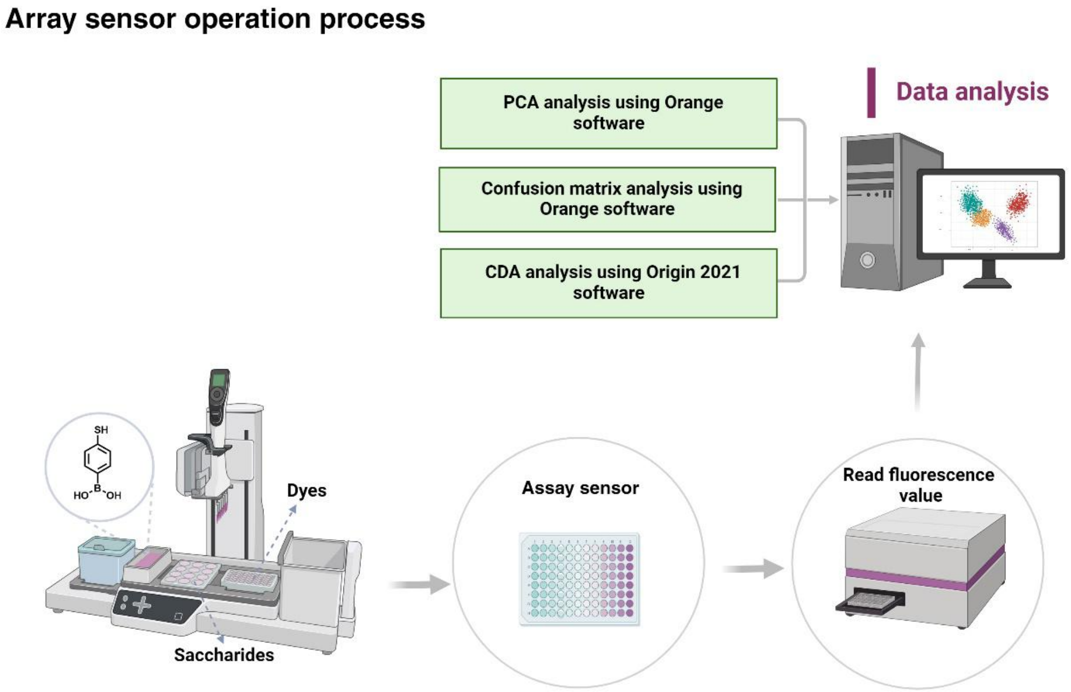

Supplementary Scheme 1. The schematic diagram of the operation process of the array sensor.

2. Supporting Figures

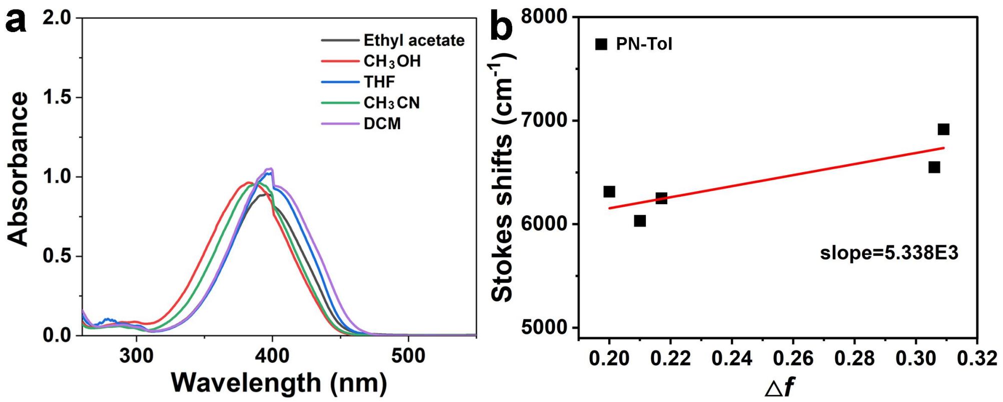

Figure S1. a-b) UV-vis absorption spectrum of PN-Tol (33 μM) in different solvents at 25 °C and Lippert–Mataga plot for PN-Tol (b).

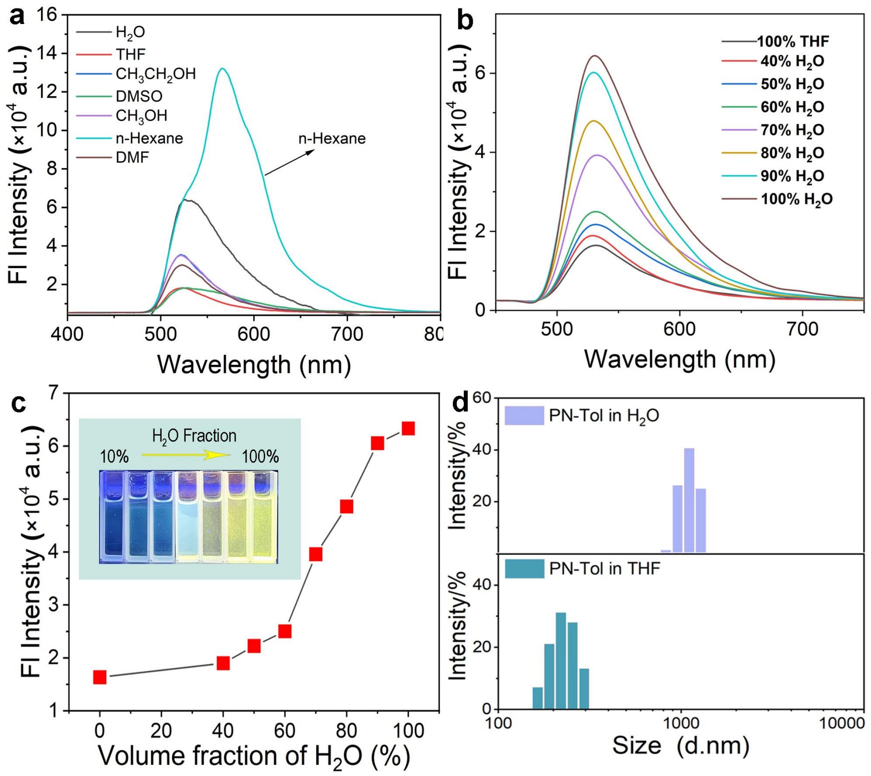

Figure S2. a) Fluorescence emission spectra of PN-Tol (33 μM) in different solvents at 25 °C, excitation wavelength (Ex): 293 nm; b, c) Fluorescence spectra (b) and changes in fluorescent intensity at 565 nm (c) of PN-Tol (33 μM) in THF/H_2O mixed solution with different volume fraction of H_2O. d)Particle size distribution of PN-Tol in H_2O and THF (1 mM), measured by dynamic light scattering at 25 °C.

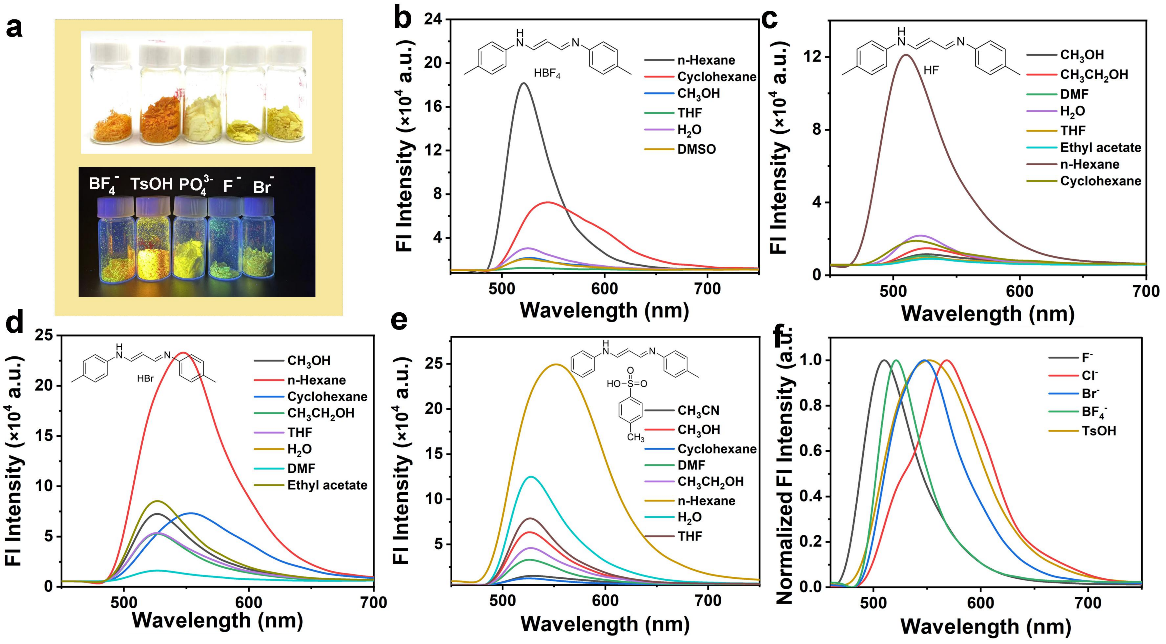

Figure S3. a) Photos of PN-Tol@Cl^-, PN-Tol@TsOH, PN-Tol@BF_4^-, PN-Tol@F^-, PN-Tol@Br^- under sunlight and ultraviolet light irradiation; b-e) Fluorescence emission spectra of PN-Tol@Cl^-, PN-Tol@TsOH, PN-Tol@BF_4^-, PN-Tol@F^-, PN-Tol@Br^- (33 μM) in different solvents at 25 °C, excitation wavelength (Ex): 293 nm; f) Normalized fluorescence emission spectra of PN-Tol@Cl^-, PN-Tol@Br^-, PN-Tol@F^-, PN-Tol@BF_4^-, PN-Tol@TsOH (33 μM) in *n*-hexane at 25 °C.

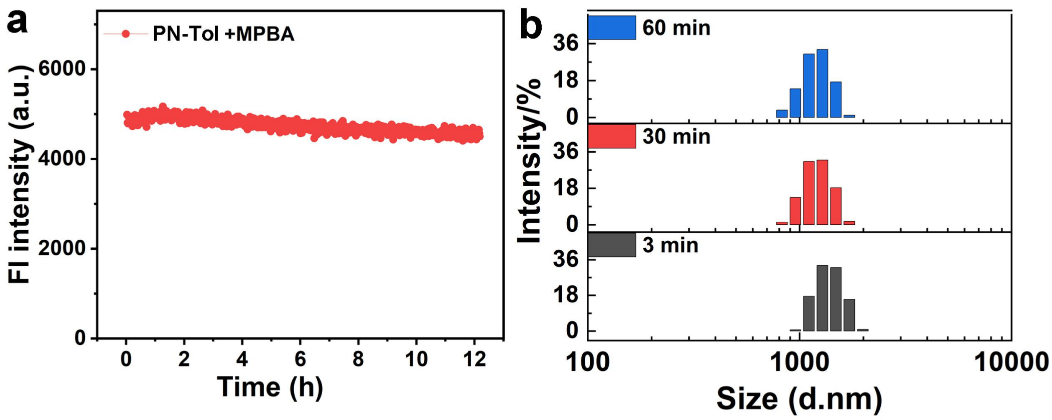

Figure S4. a) The fluorescence intensity value of PN-Tol@MPBA in Tris-HCl buffer (10 mM, pH=6.8) was tested continuously for 12 hours, with a total of 731 tests conducted, intervals of 1 minute. Excitation wavelength 385 nm, emission 565 nm. b) Particle size distribution of PN-Tol@MPBA in water (1 mM), measured by dynamic light scattering at 25 °C, the initial test needs to be stable for 3 minutes, and then data is recorded every half hour.

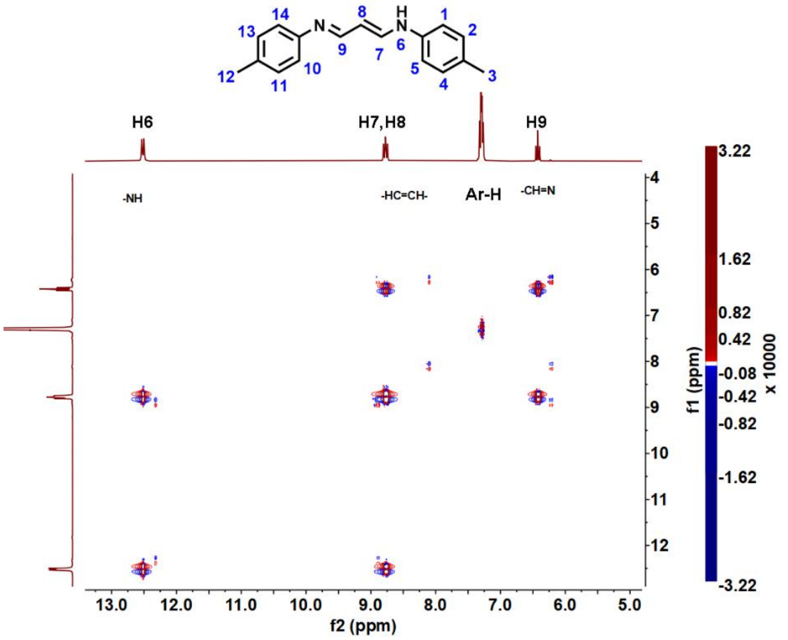

Figure S5. The 2D ^{1}H–^{1}H COSY NMR spectra of PN-Tol in d_6-DMSO at 25 ℃. The concentration is 40 mg·mL^{-1}.

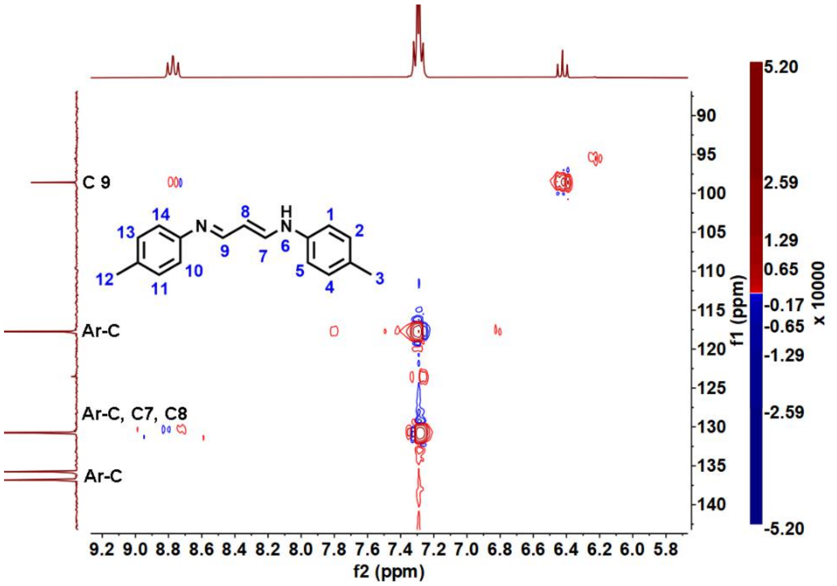

Figure S6. The 2D ^{13}C–^{1}H HSQC NMR spectra of PN-Tol in d_6-DMSO at 25 ℃. The concentration is 60 mg·mL^{-1}.

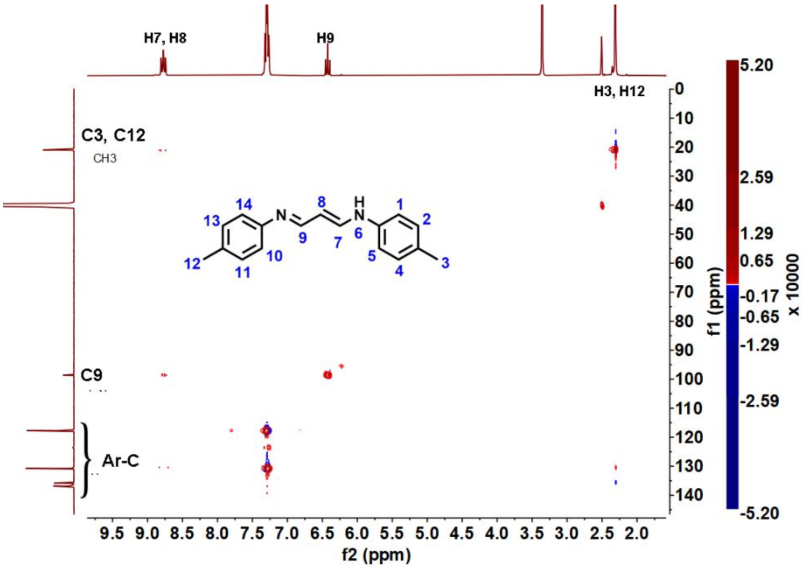

Figure S7. The 2D ^{13}C–^{1}H HSQC NMR spectra of PN-Tol in d_6-DMSO at 25 ℃. The concentration is 60 mg·mL^{-1}.

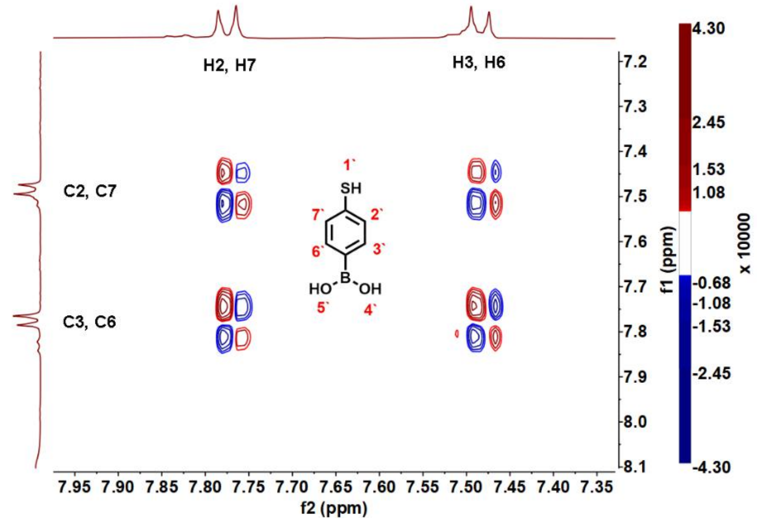

Figure S8. The 2D ^{1}H–^{1}H COSY NMR spectra of MPBA in d_6-DMSO at 25 ℃. The concentration is 40 mg·mL^{-1}.

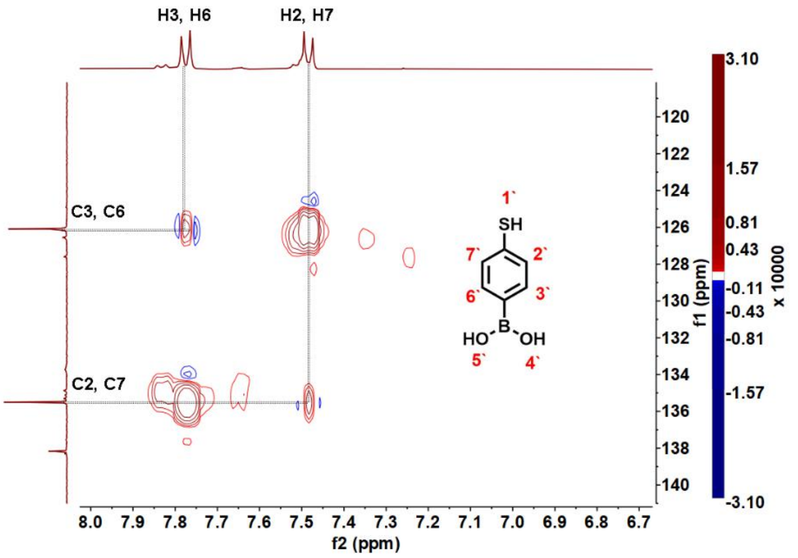

Figure S9. The 2D ^{13}C–^{1}H HSQC NMR spectra of MPBA in d_6-DMSO at 25 ℃. The concentration is 60 mg·mL^{-1}.

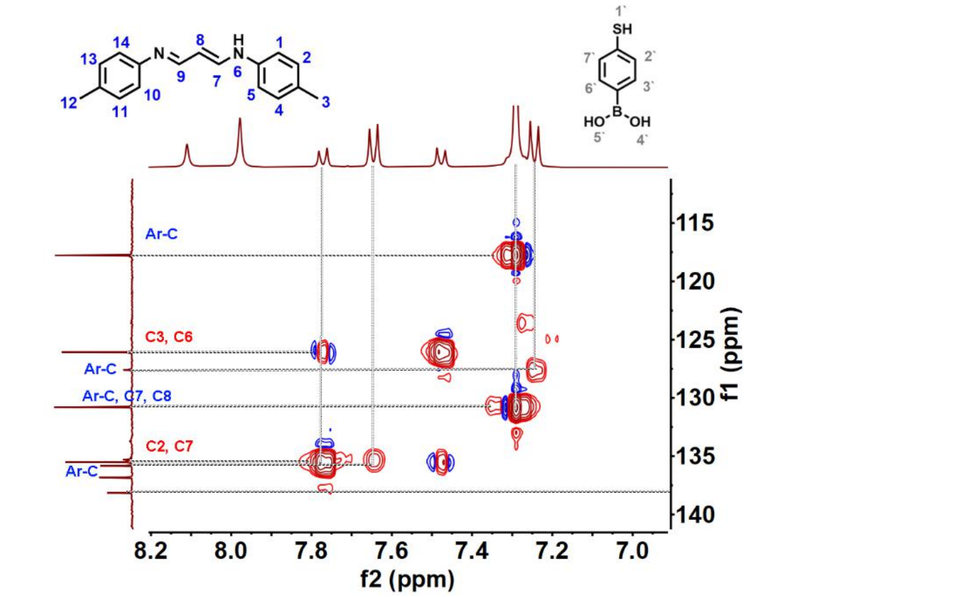

Figure S10. 2D HSQC ^{13}C-^{1}H NMR spectra of PN-Tol mixed with MPBA in DMSO-d_6 at 25 ℃; The concentration is 60 mg·mL^{-1}. The blue font represents compound PN-Tol, and the red font represents MPBA.

The combined 2D HSQC ^{13}C-^{1}H and 2D ^{1}H–^{1}H COSY NMR spectrum of PN-Tol and MPBA allows for the definitive assignment of the ^{1}H NMR spectrum of PN-Tol mixed MPBA. The proton peak at 7.48 ppm and 7.76 ppm corresponded to hydrogen on the benzene ring (Ar-H) of MPBA, and the proton peak at 7.97 ppm and 8.11 ppm corresponding to boron hydroxyl group (B-

OH). The hydrogen proton at 7.23, 7.28 ppm, and 7.63 ppm can be attributed to the hydrogen on the benzene ring (Ar-H) of PN-Tol.

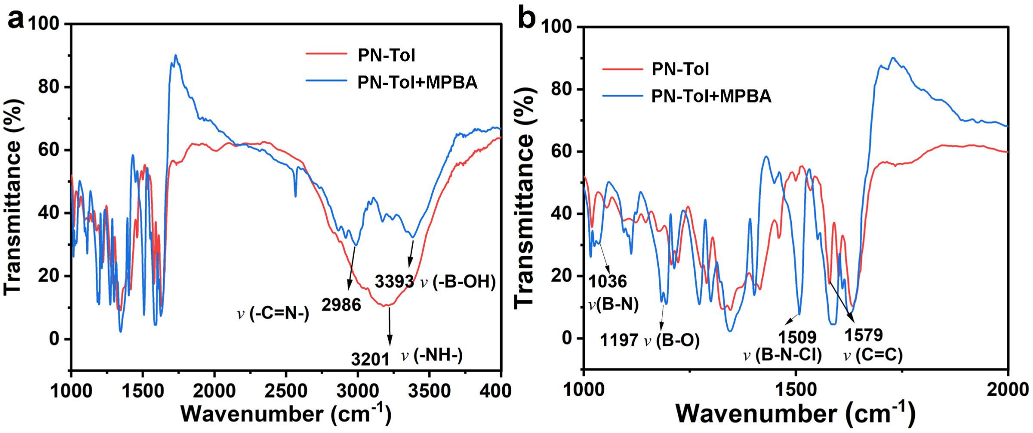

Figure S11. a. b) Infrared spectra in the 1000–4000 (a) and 1000–2000 cm^{-1} (b); Regions of PN-Tol and PN-Tol@MPBA measured by the KBr pellet pressed, KBr pellet pressed drying method.

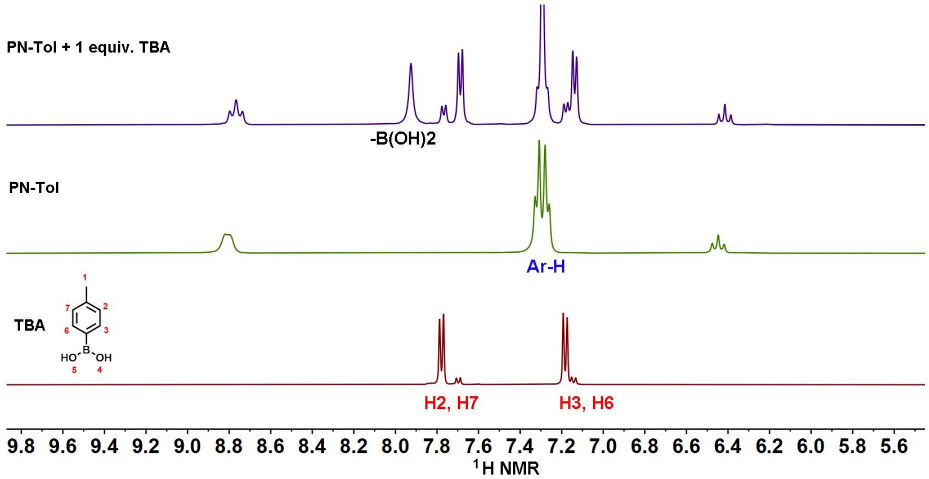

Figure S12. ^{1}H NMR spectra of PN-Tol (40 mg/mL), TBA (40 mg/mL), and PN-Tol after adding 1 equiv. TBA in d_6-DSMO at 25 ℃, respectively; The blue typeface represents the H attribution of PN-Tol, and the red typeface represents the H attribution of TBA.

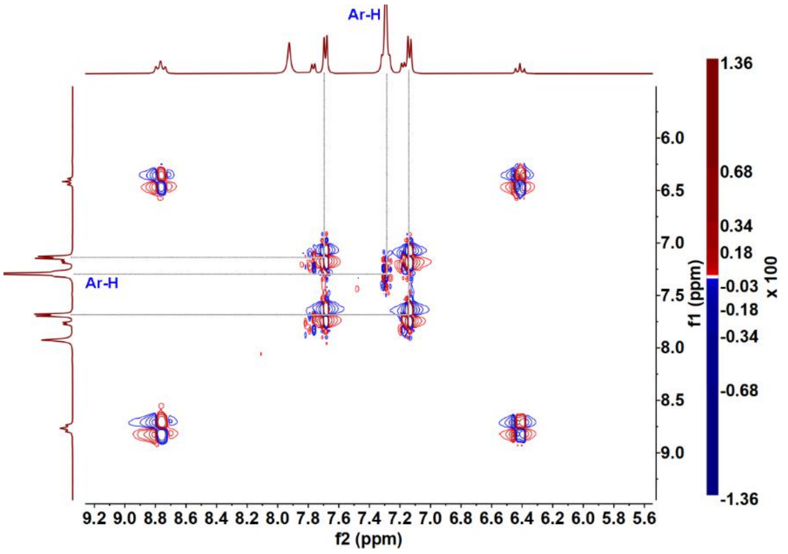

Figure S13. 2D COSY ^{1}H-^{1}H spectra of PN-Tol mixed with equimolar TBA (0.4 M) in d_6-DSMO at 25 ℃.

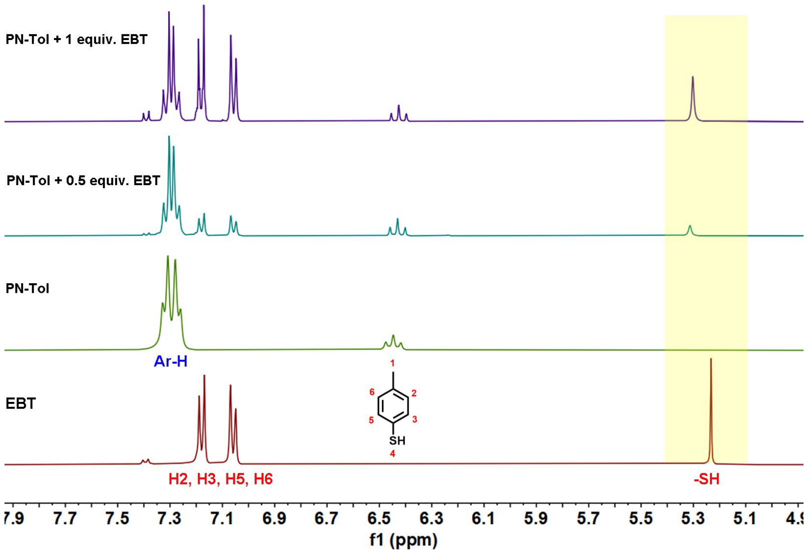

Figure S14. ^{1}H NMR spectra of PN-Tol (40 mg/mL), EBT (40 mg/mL), and PN-Tol after adding 0.5 equiv. and 1 equiv. EBT in d_6-DSMO at 25 ℃, respectively; The blue typeface represents the H attribution of PN-Tol, and the red typeface represents the H attribution of EBT.

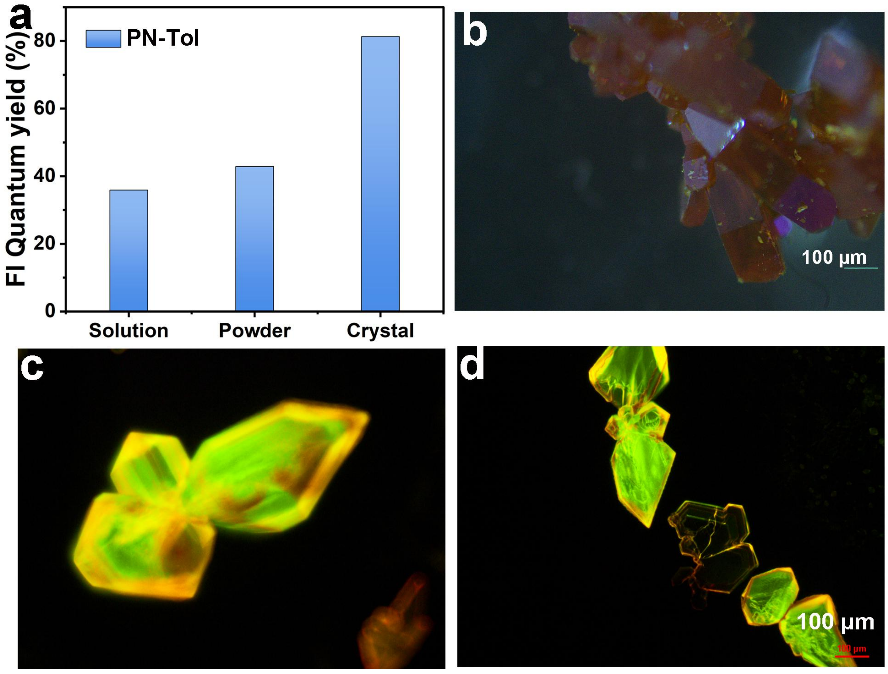

Figure S15. a) Fluorescence quantum yields of PN-Tol (1.2 mM) in liquid, powder and crystalline states; b) Microscopic imaging of PN-Tol crystals; c, d) Fluorescence microscopy imaging of PN-Tol crystals, using green channels as excitation light.

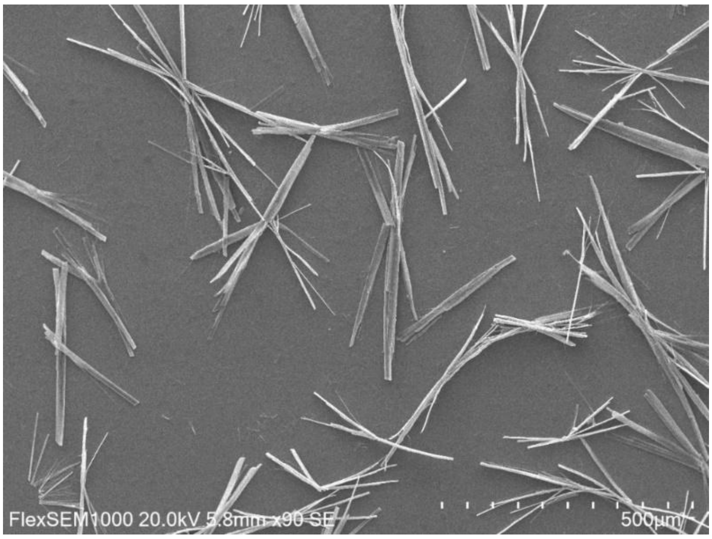

Figure S16. Self-assembly morphology of PN-Tol on a silica wafer surface, formed in CH_3OH solution (5 mM) contains 10% DMSO.

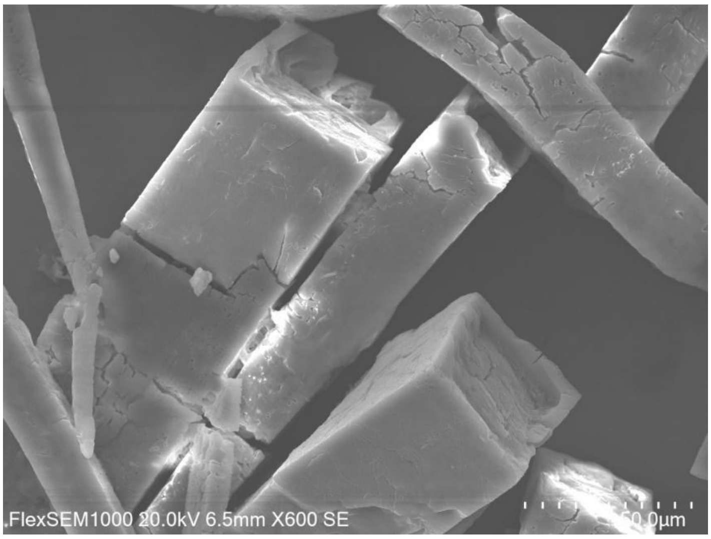

Figure S17. Self-assembly morphology of PN-Tol on a silica wafer surface, formed in CH_3OH solution (10 mM) containing 10% DMSO.

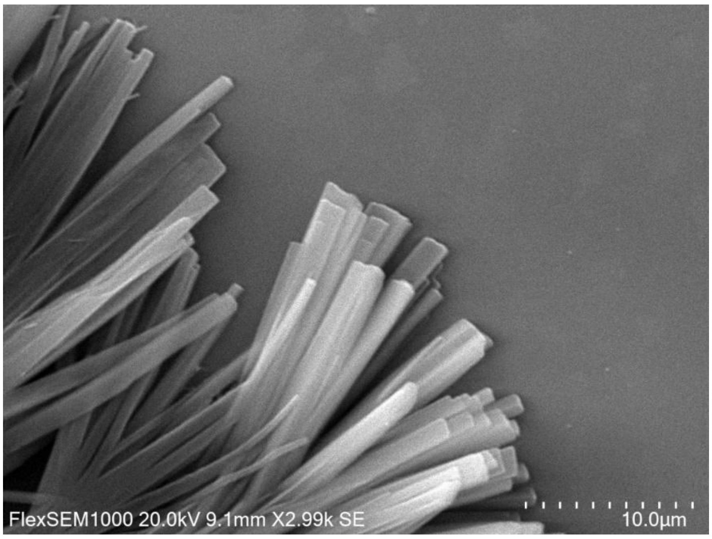

Figure S18. Self-assembly morphology of PN-Tol on a silica wafer surface, formed in THF solution (5 mM) contains 10% DMSO.

Figure S16-18 illustrates that PN-Tol can form rectangular layered assemblies even at different concentrations and in different solvents. Furthermore, with an increase in concentration (from 2.5 mM to 10 mM), the rectangular assemblies further enlarge, transitioning from a layered structure to a three-dimensional form, resembling a cuboid structure. This underscores the potent self-assembly capability and controllable self-assembly process of PN-Tol. The quest for suitable self-

assembling modules has been a central issue in modern nanotechnology[3]. Once in a solution, the appropriate building blocks assemble in a highly precise manner to generate larger supramolecular entities tailored for specific functionalities.

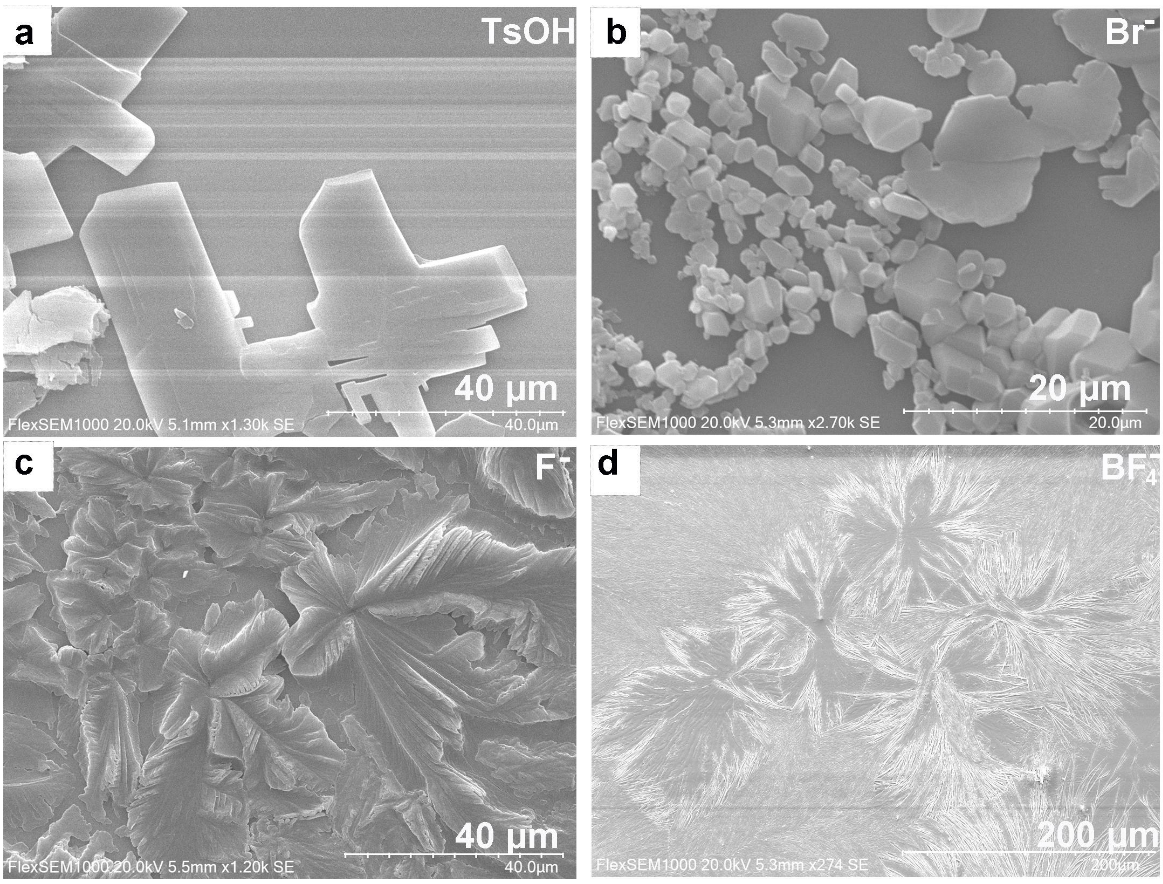

Figure S19. a-d) Self-assembly morphology of PN-Tol@TsOH (a), PN-Tol@Br^- (b), PN-Tol@F^- (c), PN^-Tol@BF_4^- (d) on a silica wafer surface, formed in CH_3OH solution (10 mM) containing 10% DMSO.

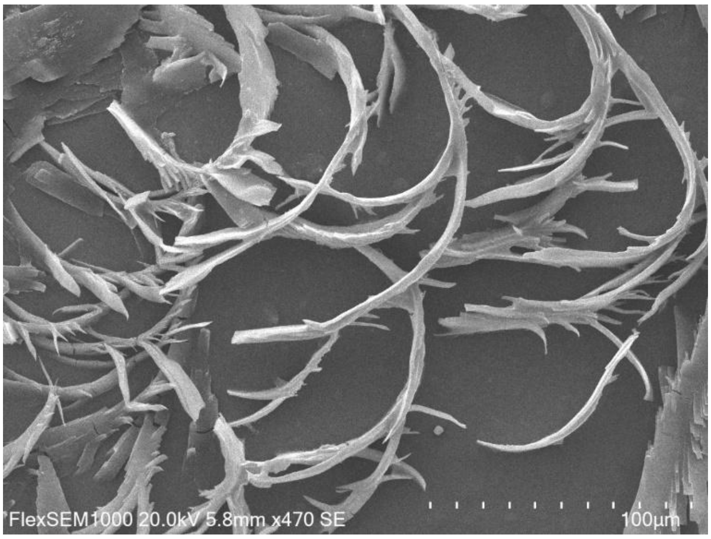

Figure S20. SEM images of PN-Tol mixed with MPBA interfacial assemblies on a silica wafer surface, formed in CH_3OH solution (5 mM) containing 10% DMSO.

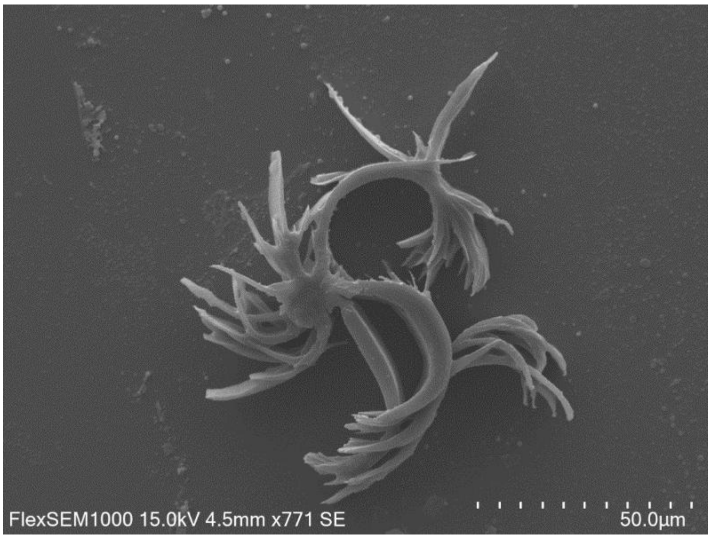

Figure S21. SEM images of PN-Tol mixed with MPBA interfacial assemblies on a silica wafer surface, formed in CH_3OH solution (10 mM) containing 10% DMSO.

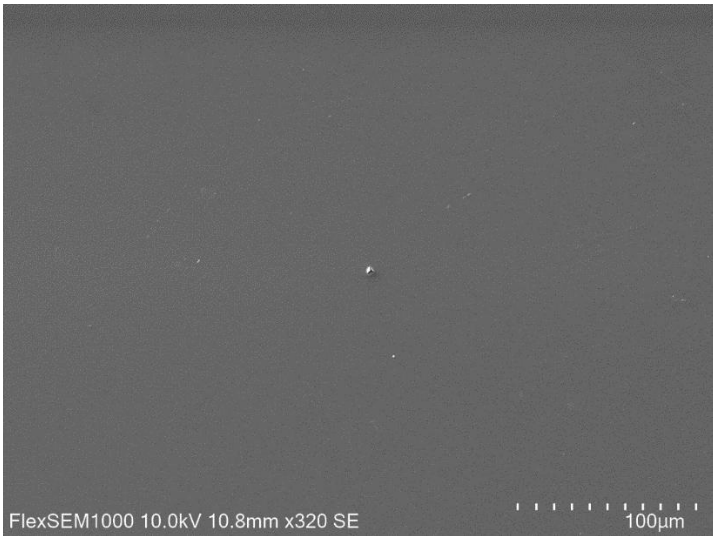

Figure S22. SEM images of MPBA interfacial assemblies on a silica wafer surface, formed in CH_3OH solution (2.5 mM).

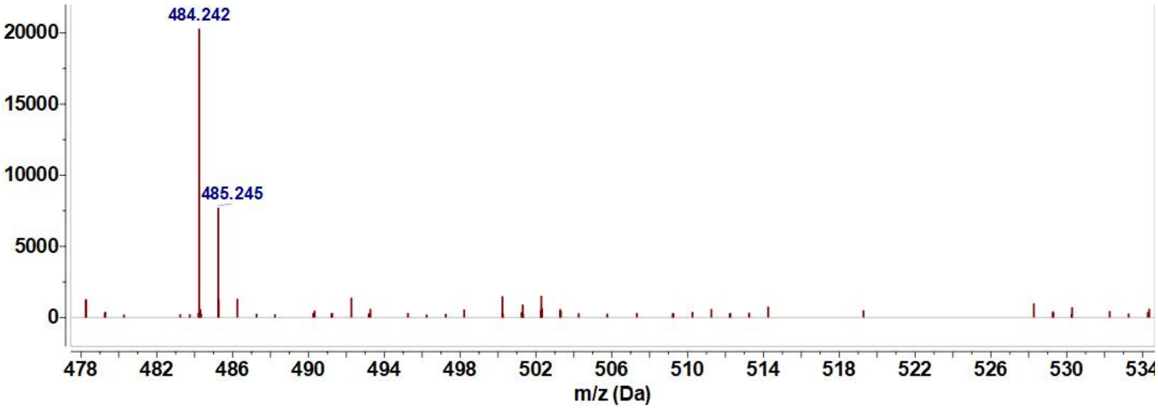

Figure S23. Mass spectrometry of complexes of Neu5Ac and MPBA, Calcd [MPBA+Neu5Ac+Na]$^{+}$: 484.250, found: 484.242.

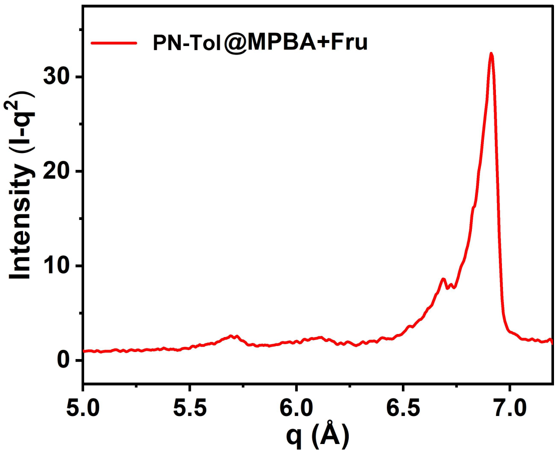

Figure S24. Structure factors of PN-Tol@MPBA binding Fru (red) calculated from SAXS data.

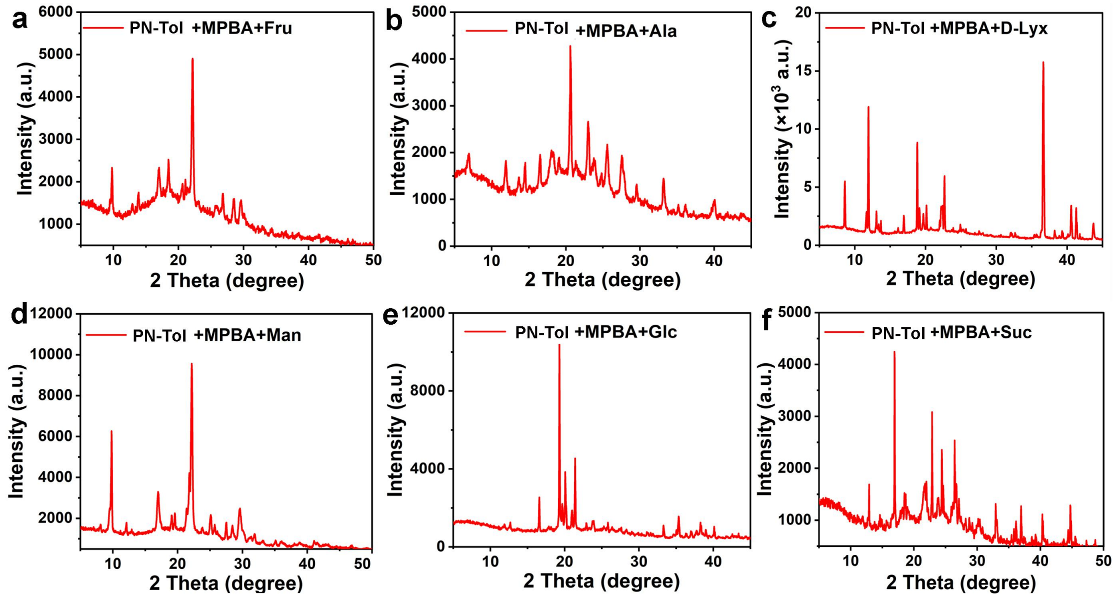

Figure S25. a-f) XRD patterns of the PN-Tol@MPBA composite Fru (a), Ala (b), Lyx (c), Man (d), Glc (e), and Suc (f) samples.

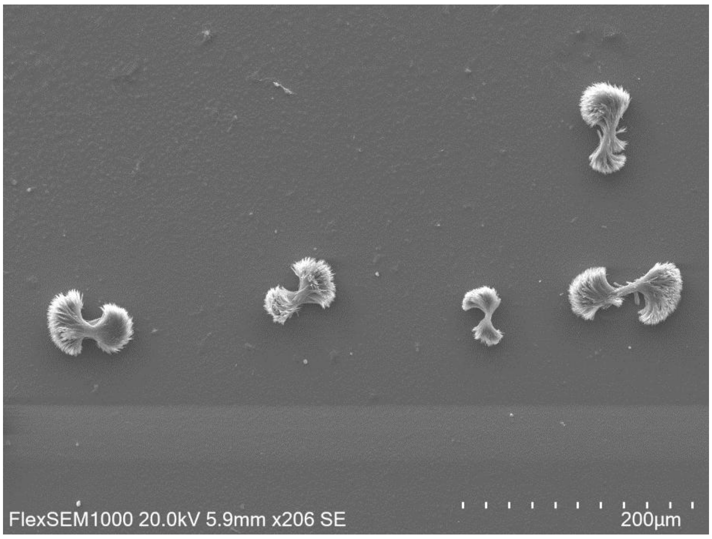

Figure S26. SEM images of triple assembly of PN-Tol@MPBA binding D-Fru on a silica wafer surface, self-assembled in CH_3OH containing 10% DMSO at 2.5 mM.

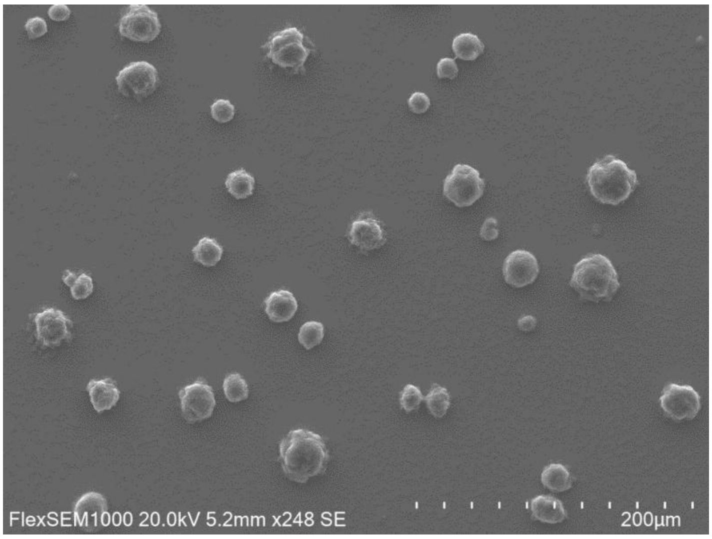

Figure S27. SEM images of triple assembly of PN-Tol@MPBA binding Ara on a silica wafer surface, self-assembled in CH_3OH contain 10% DMSO at 2.5 mM.

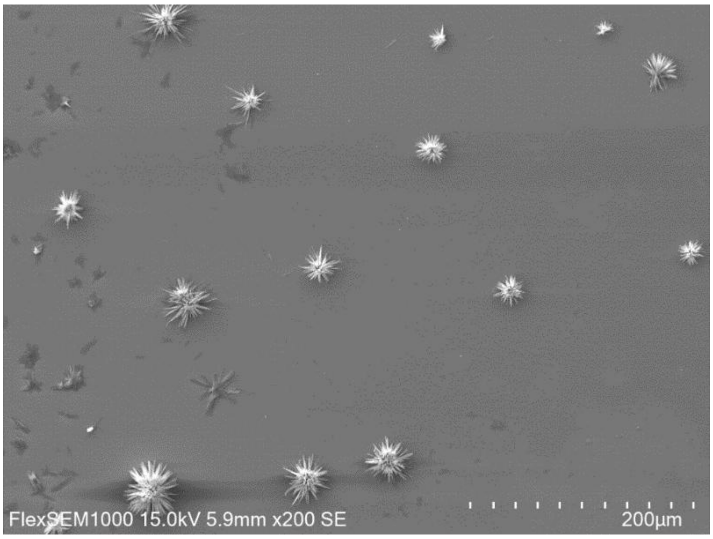

Figure S28. SEM images of triple assembly of PN-Tol@MPBA binding Lyx on a silica wafer surface, self-assembled in CH_3OH contain 10% DMSO at 2.5 mM.

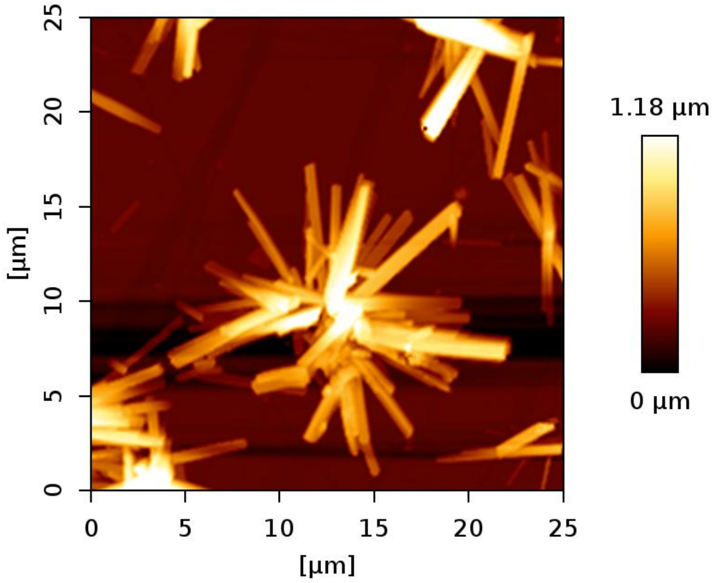

Figure S29. AFM images of triple assembly of PN-Tol@MPBA binding Lyx on a mica sheet surface, self-assembled in CH_3OH contain 10% DMSO at 2.5 mM.

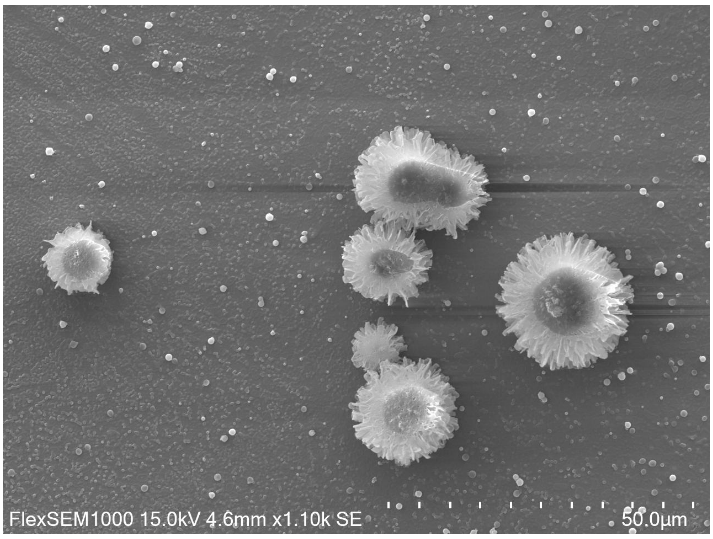

Figure S30. Triple assembly morphology of PN-Tol@MPBA binding Man, the assembly formed in CH_3OH solution contains 10% DMSO, and the concentration is 2.5 mM.

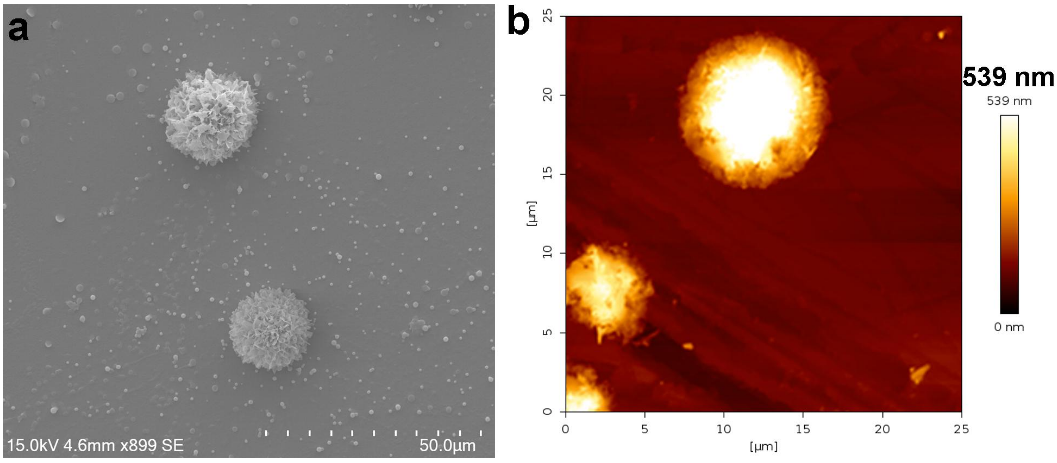

Figure S31. a, b) SEM image and AFM image of triple assembly of PN-Tol@MPBA binding Glc, the assembly formed in CH_3OH solution contains 10% DMSO and the concentration is 2.5 mM.

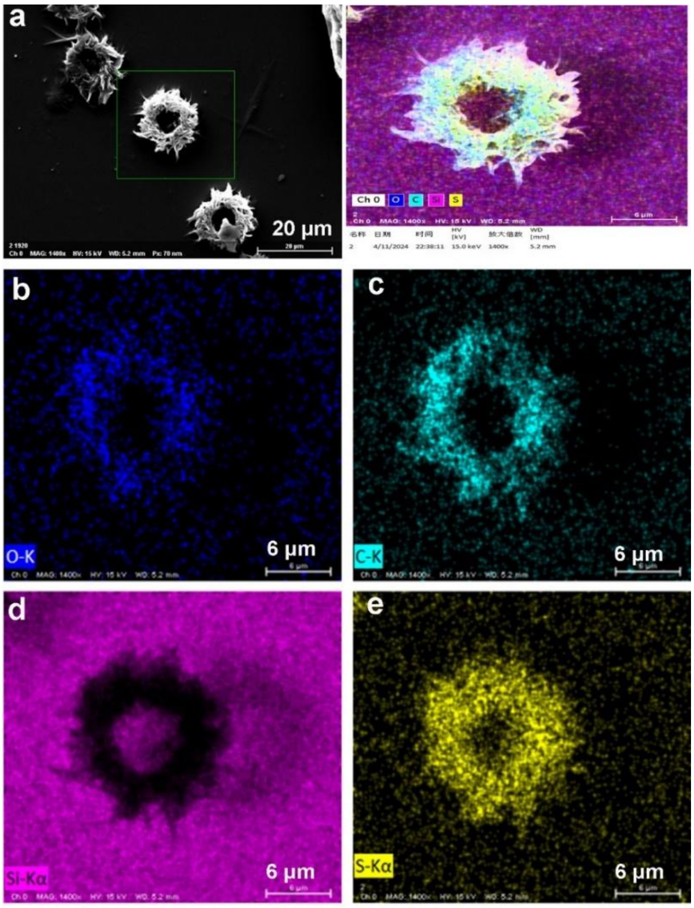

Figure S32. SEM image of PN-Tol@MPBA binding Gal (a) and corresponding EDS elemental mappings images of (b) oxygen, (c) carbon, (d) silicon, and (e) sulfur. the assembly is formed in CH_3OH solution and the concentration is 2.5 mM.

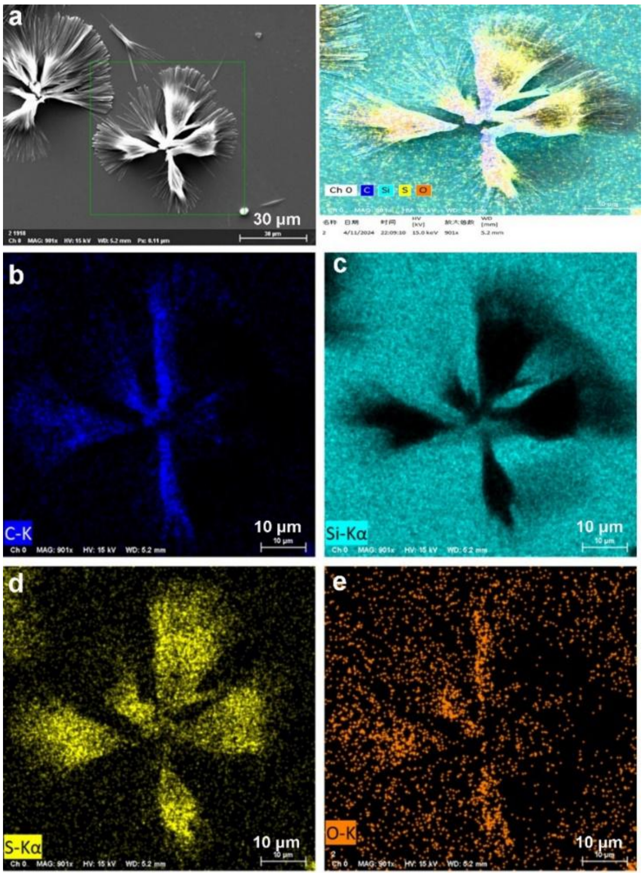

Figure S33. SEM image of PN-Tol@MPBA binding Suc (a) and corresponding EDS elemental mappings images of (b) carbon, (c) silicon, (d) sulfur and (e) oxygen. the assembly formed in CH_3OH solution and the concentration is 2.5 mM.

Figures S32 and S33 show that the ternary co-assembly contains elements such as oxygen, sulfur, and carbon. The oxygen originates from the corresponding saccharides, sulfur from MPBA, and carbon from both PN-Tol and MPBA and saccharides. This indicates that the assembly is synergistically constituted by all three components, further confirming the formation of the ternary co-assembly.

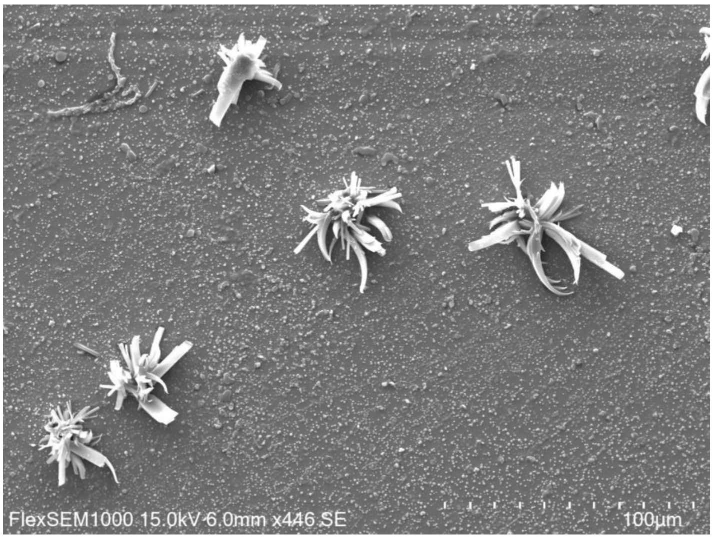

Figure S34. SEM images of triple assembly of PN-Tol@MPBA binding Tre on a silica wafer surface, self-assembled in CH_3OH contain 10% DMSO at 2.5 mM.

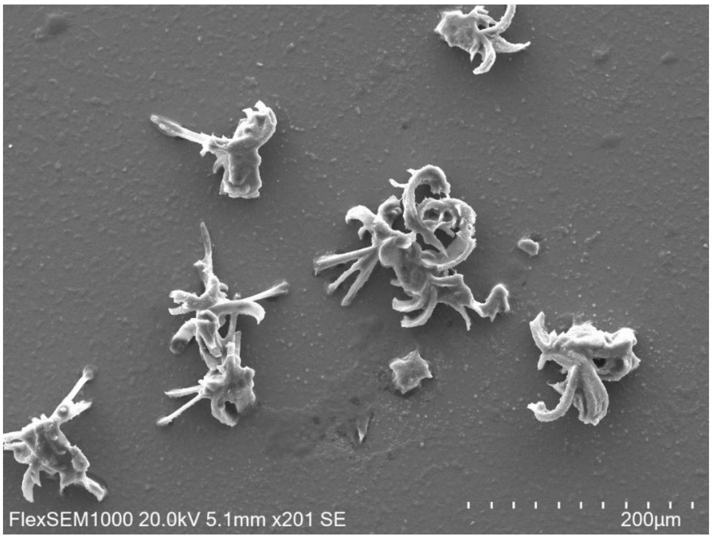

Figure S35. SEM images of triple assembly of PN-Tol@MPBA binding Tre on a silica wafer surface, self-assembled in CH_3OH contain 10% DMSO at 2.5 mM.

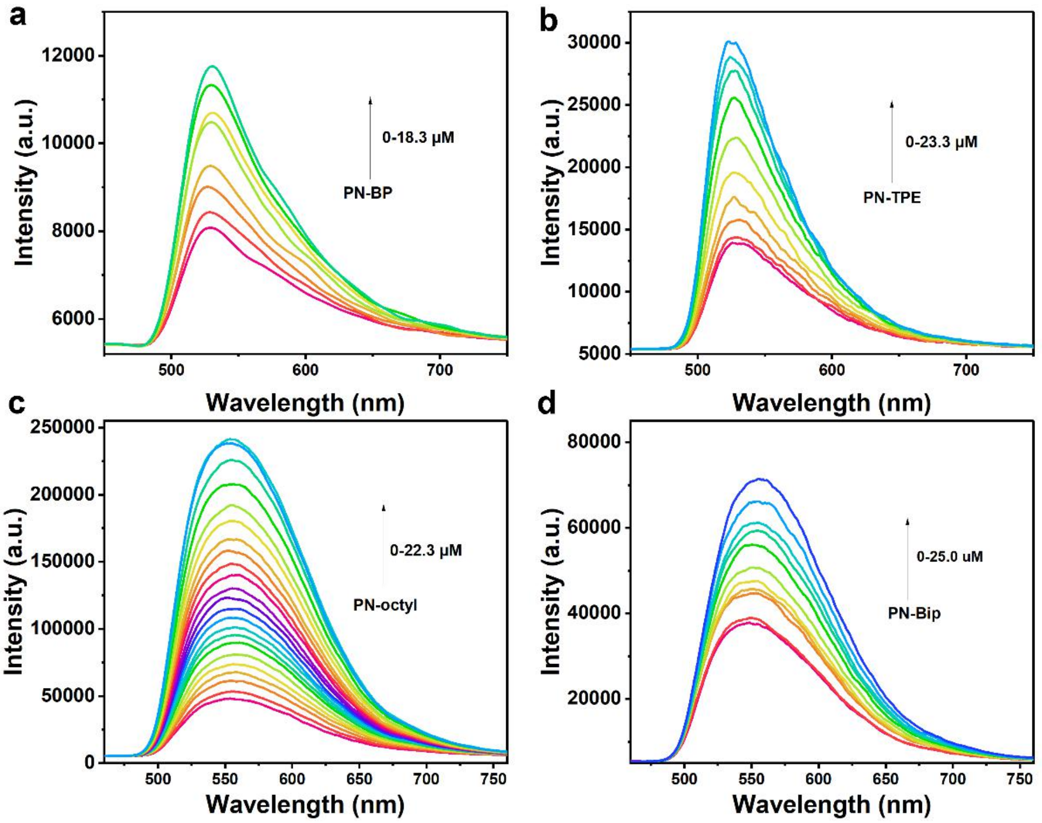

Figure R36. a-d) Fluorescence spectra of PN-BP (a), PN-TPE(b), PN-Boctyl (c) and PN-BIP (d) after the addition of different concentrations of MPBA in Tris-HCl buffers (10 mM, pH 6.8) are used as the solutions and the test temperature is 25 °C.

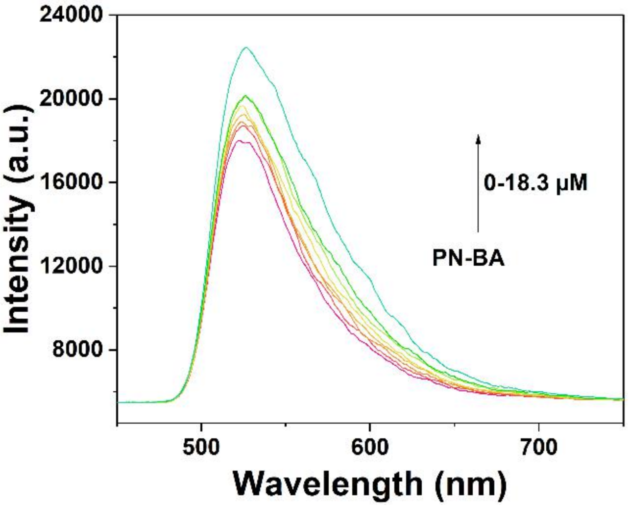

Figure R37. Fluorescence spectra of PN-BA after the addition of different concentrations of MPBA (0–18.3 μM) in Tris-HCl buffers (10 mM, pH 6.8).

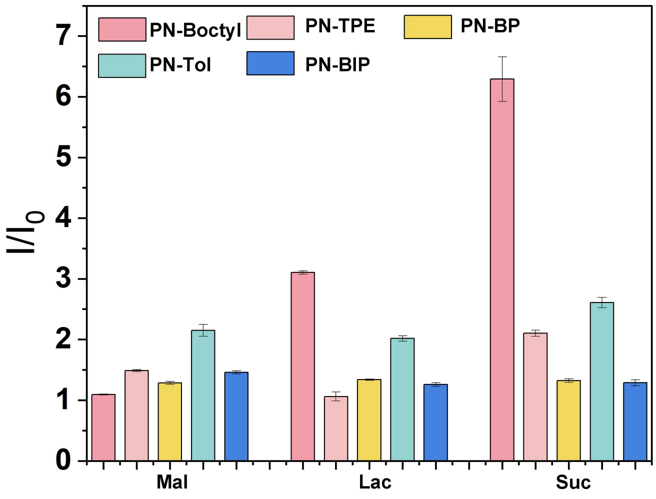

Figure S38. Fluorescence responses (I) upon addition of the Mal, Lac, and Suc to the array sensor in Tris-HCl buffer solution (10 mM), I_0= Initial fluorescence value. Error bars represent mean ± s.d. (n = 6 independent experiments).

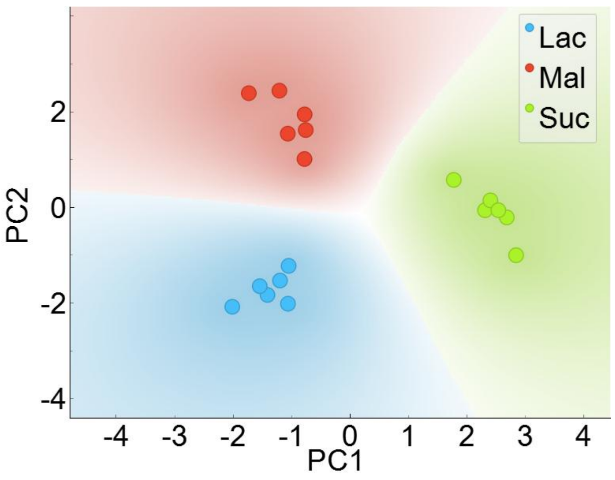

Figure S39. PCA scores plot from the fluorescence responses of Lac, Mal, and Suc in Tris-Hcl (10 mM, pH=6.8) buffer.

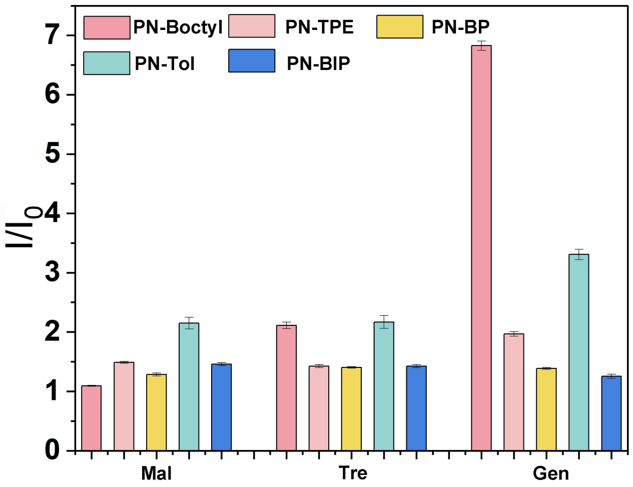

Figure S40. Fluorescence responses *(I)* upon addition of the Mal, Tre, and Gen to the array sensor in Tris-HCl buffer solution (10 mM), I_0= Initial fluorescence value. Error bars represent mean ± s.d. (n = 6 independent experiments).

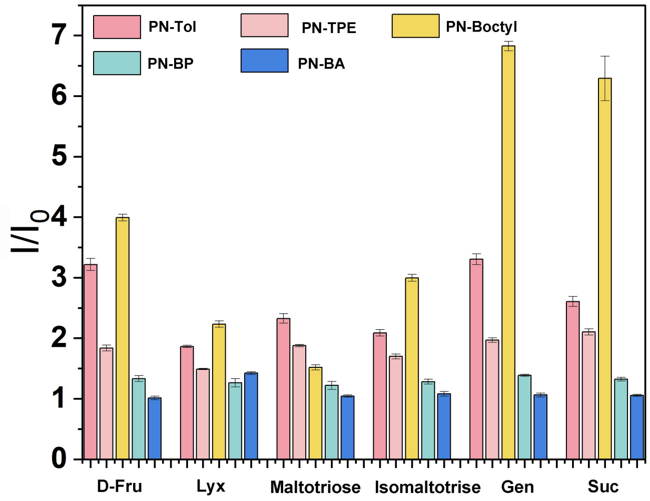

Figure S41. Fluorescence responses *(I)* upon addition of the D-Fru, Lyx, Maltotriose, Isomaltotrise, Gen, and Suc to the array sensor in Tris-HCl buffer solution (10 mM), I_0= Initial fluorescence value. Error bars represent mean ± s.d. (n = 6 independent experiments). Each array component shows a different fluorescence response and this array of responses can be subjected to multivariate analysis to differentiate saccharides.

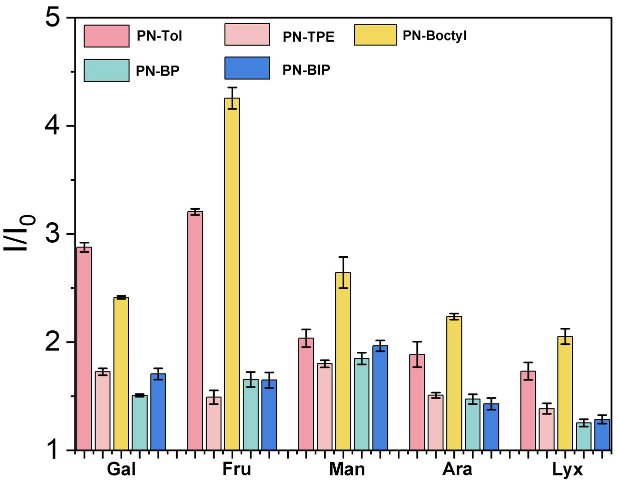

Figure S42. Fluorescence responses (*I*) upon addition of the Gal, Fru, Man, Ara and Lyx to the array sensor in Tris-HCl buffer solution (10 mM) in serum mixture, I_0= Initial fluorescence value. Error bars represent mean ± s.d. (n = 6 independent experiments).

Confusion matrix for SVM (showing number of instances)

		Predicted					
		**Ala**	**Fru**	**Gal**	**Lyx**	**Man**	**∑**
Actual	**Ala**	6	0	0	0	0	**6**
	**Fru**	0	6	0	0	0	**6**
	**Gal**	0	0	6	0	0	**6**
	**Lyx**	0	0	0	6	0	**6**
	**Man**	0	0	0	0	6	**6**
	**∑**	**6**	**6**	**6**	**6**	**6**	**30**

Figure S43. Confusion matrix of the PCA analysis of Gal, Fru, Man, Ara and Lyx in serum mixture. Correct predictions are represented by on-diagonal blue squares, while incorrect predictions are represented by off-diagonal white squares.

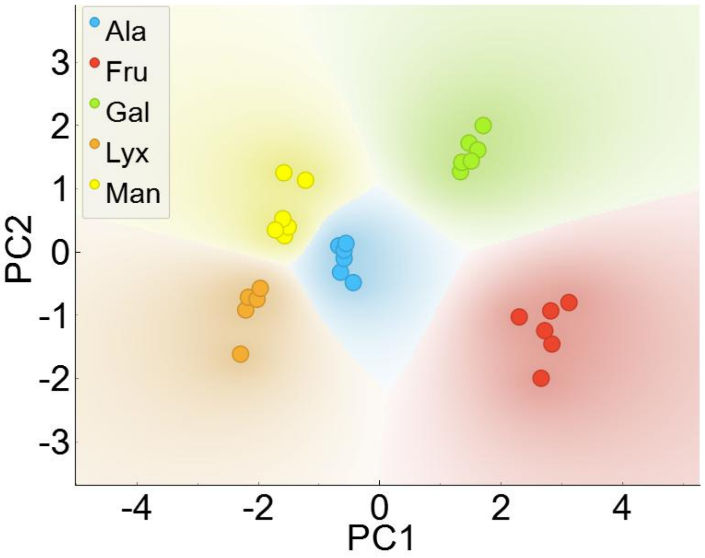

Figure S44. PCA scores plot of Gal, Fru, Man, Ara and Lyx (5μM) in serum mixture.

3. Supporting Tables

3.1 Table 1. Crystal data and structure refinements for PN-Tol and PN-Tol@thiophenol.

	**PN-Tol**	**PN-Tol@thiophenol**
**Space group**	$P2_1/n$	$P\bar{1}$
**CCDC**	2192975	2357101
***Cell Length a*(Å)**	6.646(2)	8.9031(17)
***Cell Length b* (Å)**	15.353(6)	7.3609(15)
***Cell Length c* (Å)**	18.683(8)	15.481(3)
***Cell angle* α (°)**	90	94.973(7)
***Cell angle* β (°)**	92.973(17)	92.605(7)
Cell angle* γ *(°)	90	91.805(7)
***Cell Volume* ($Å^3$)**	1903.8(12)	1009.1(3)
Z	4	2
F(000)	752.0	418
Density (calculated) (g/cm³)	1.22	1.613
**T_{min}, T_{max}**	0.973, 0.977	0.6648, 0.7454

More detailed crystallographic data for this paper is available: CCDC-2192975, CCDC-2357101 which contains the supplemental crystallographic data for this paper. These data can be obtained free of charge from the Cambridge Crystallographic Data Centre via http://www.ccdc.cam.ac.uk/data_request/cif.

3.2 Table 2. Spectral parameters of PN-Tol and PN-Tol@MPBA.

No.	Compounds	λ_{Ex} (nm)	λ_{Em} (nm)	Stokes Shifts (nm)	Fluorescence lifetime	Quantum yield
1	PN-Tol	293	565	269	τ= 1.43 ns	35.9%
2	PN-Tol@MPBA	291	563	272	τ_1 = 1.33<br>τ_2 = 3.21	42.9%

4. Synthetic procedures and characterization data

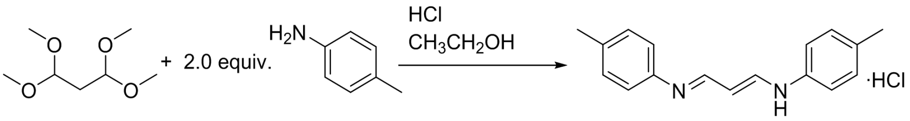

Scheme 2. Synthetic pathway for PN-Tol.

4.1 Synthesis of PN-Tol:

To a solution of *p*-Toluidine (0.64 g, 6.0 mmol) in ethanol (20 mL) at 20 °C was added tetramethoxypropane (0.49 g, 3.0 mmol). Then, conc. hydrochloric acid (3 mL) was added and the resultant mixture was stirred at room temperature for 5 hours. After that, the reaction solution was cooled to room temperature, and a large amount of yellow precipitate was obtained by recrystallization. The precipitate was filtered and washed repeatedly with 100 mL ethanol. Yellow powder (0.64 g) was obtained after drying in a vacuum with a yield of 84 %. ^{1}H NMR (400 MHz, d_6-DMSO) δ: 12.43 (d, J = 13.9 Hz, 2H), 8.70 (d, J = 11.5 Hz, 2H), 7.29 (s, 8H), 6.38 (t, J = 11.5 Hz, 1H), 2.31 (s, 6H); ^{13}C NMR (101 MHz, d_6-DMSO) δ: 158.64, 137.83, 133.64, 128.63, 116.20, 100.10, 18.76; MS (ESI) m/z: calcd. for $C_{17}H_{18}N_2{}^{+}$: 250.190; found: 251.173 $[M+H]^{+}$.

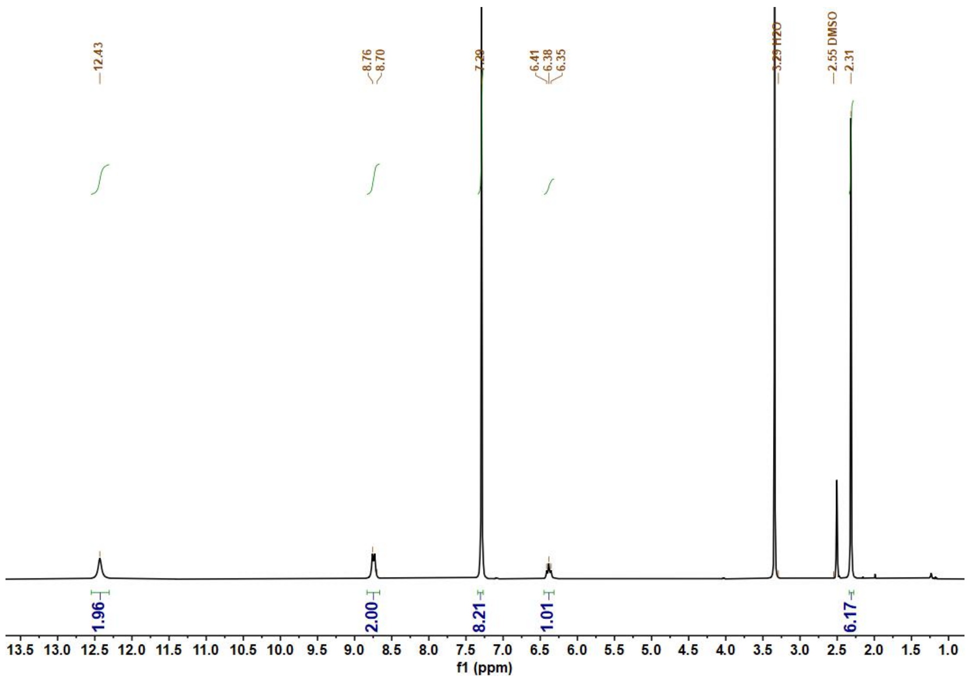

Figure S45. ^{1}H NMR (400 MHz) spectrum of **PN-Tol** in d_6-DMSO at 25 ℃.

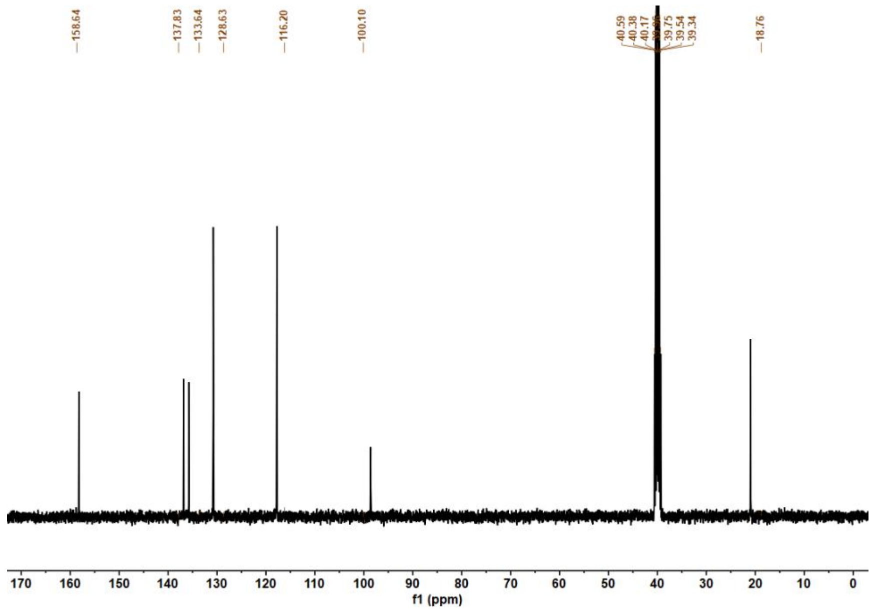

Figure S46. ^{13}C NMR (101 MHz) spectrum of **PN-Tol** in d_6-DMSO at 25 ℃.

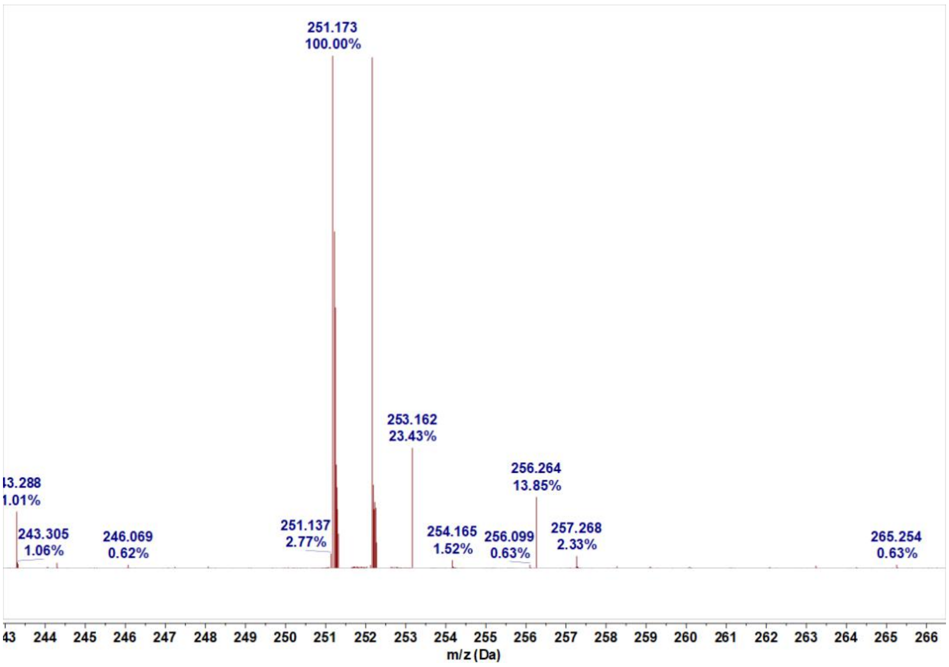

Figure S47. MS spectrum of **PN-Tol** Calcd. for $C_{17}H_{18}N_2$: 250.190, found: 251.173 $[M+H]^+$.

4.2 Synthesis of PN-BA:

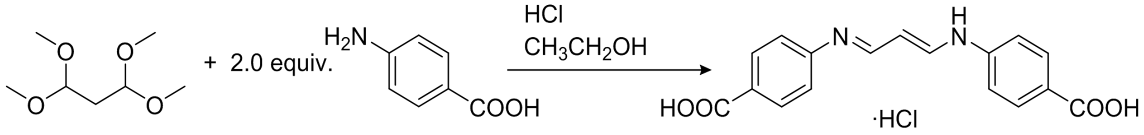

Scheme 3. Synthetic route of **PN-BA**.

PN-BA was synthesized according to similar methods as that described for 4.1 synthesis. Briefly, to a solution of 4-aminobenzoic acid (0.83 g, 6.0 mmol) in ethanol (20 mL) was added tetramethoxypropane (0.50 g, 3 mmol) and Conc. hydrochloric acid (3 mL), and the resultant mixture was stirred at room temperature for 2 hours. After that, a large amount of yellow precipitate was obtained, filtered, and washed with 300 mL of ethanol. The product was dried in a vacuum to obtain the PN-BA 0.86 g as a yellow powder with a yield of 91%. ^{1}H NMR (400 MHz, DMSO-d_6) δ 12.25 (s, 2H), 8.83 (d, J = 11.5 Hz, 2H), 7.50 (t, J = 7.7 Hz, 4H), 7.41 (d, J = 7.6 Hz, 4H), 7.26 (t, J = 7.3 Hz, 2H), 6.34 (d, J = 11.5 Hz, 1H). ^{13}C NMR (100 MHz, DMSO-d_6): 166.99, 160.50, 141.54, 130.37, 128.37, 177.83, 10.23, 55.44. MS (ESI) m/z: calcd. for $C_{17}H_{14}N_2O_4$: 310.095; found: 311.109 $[M+H]^+$.

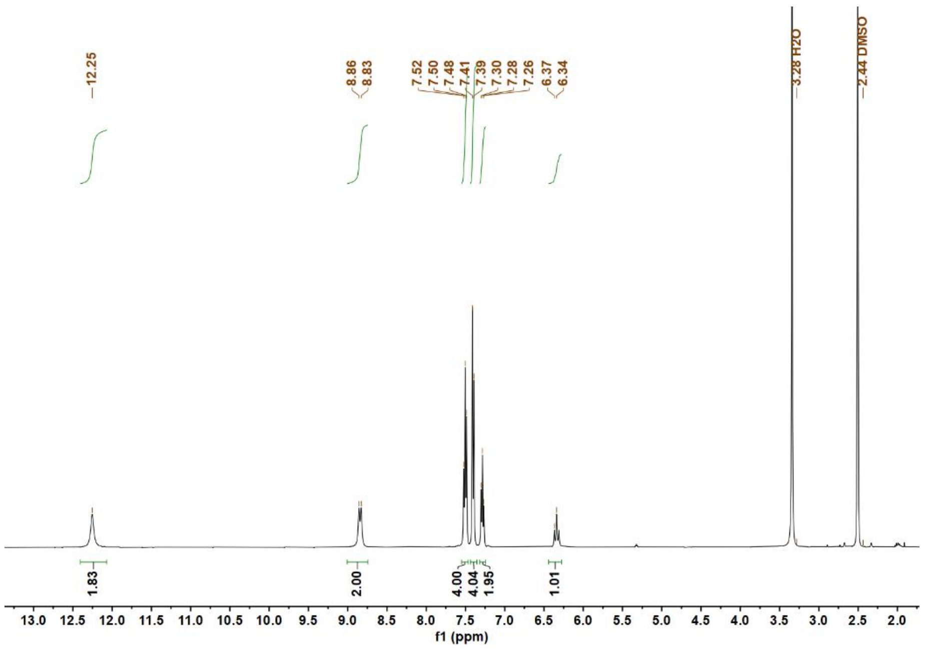

Figure S48. ^{1}H NMR (400 MHz) spectrum of **PN-BA** in d_6-DMSO at 25 ℃.

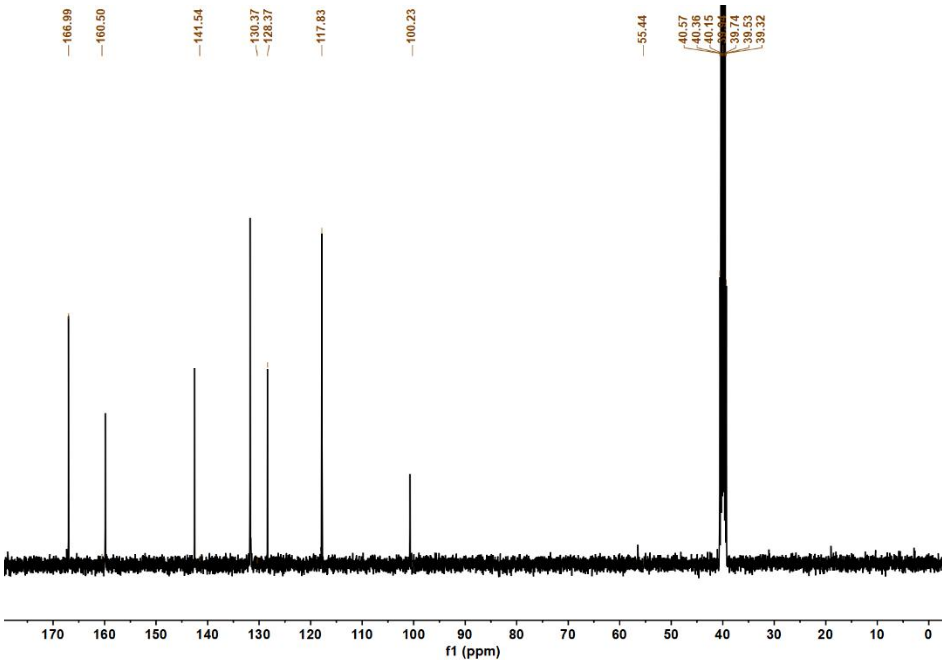

Figure S49. ${}^{13}C$ NMR (100 MHz) spectrum of **PN-BA** in d_6-DMSO at 25 ℃.

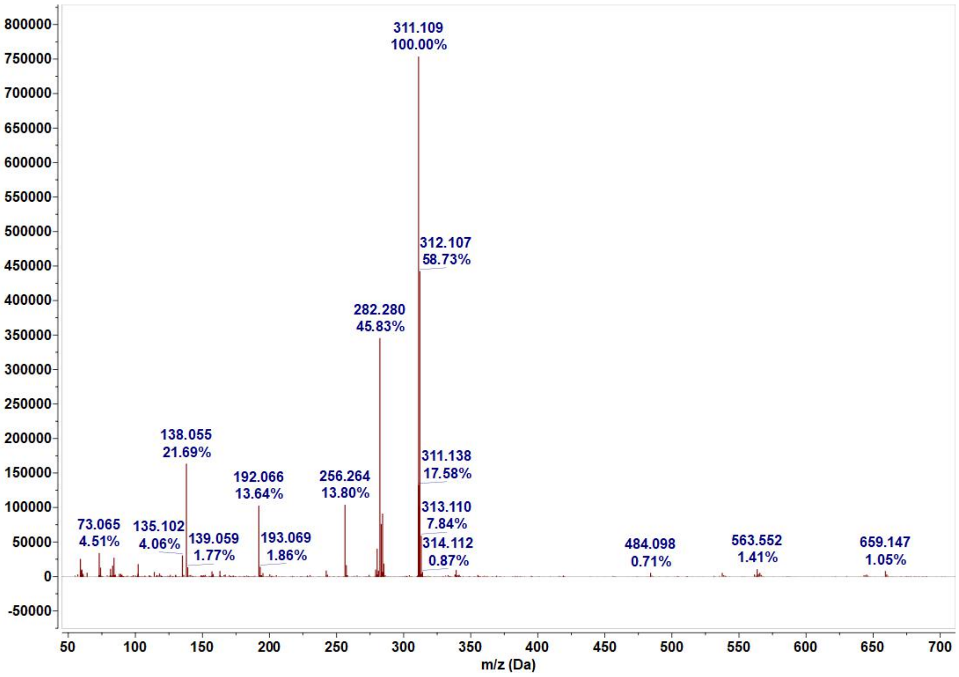

Figure S50. MS spectrum of **PN-BA** Calcd. for $C_{17}H_{14}N_2O_4$: 310.0953, found: 311.109 $[M+H]^+$.

4.3 Synthesis of PN-Boctyl:

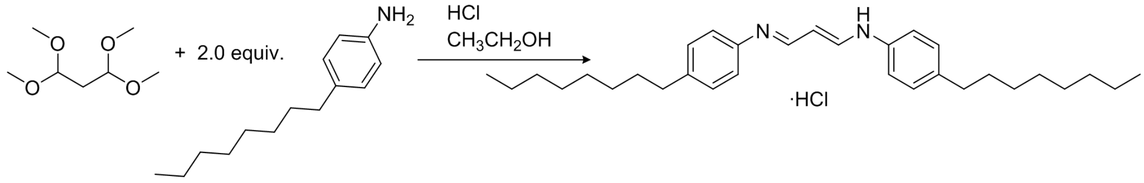

Scheme 4. Synthetic route of **PN-Boctyl**.

PN-Boctyl was synthesized according to similar methods as that described for 4.1 synthesis. Briefly, 4-n-Octylaniline (0.56 g, 3.0 mmol) and tetramethoxypropane (0.25 g, 1.5 mmol) stirred at room temperature for 8 hours were converted to PN-octyl (0.64 g) for the one-step as a yellow powder with a yield of 94.1%. ^{1}H NMR (400 MHz, d_6-DMSO) δ: 12.35 (s, 2H), 8.76 (d, J = 25.6 Hz, 2H), 7.30 (s, 8H), 6.37 (d, J = 23.1 Hz, 1H), 1.56 (s, 4H), 1.28 (s, 24H), 0.87 (s, 6H). ^{13}C NMR (176 MHz, d_6-DMSO) δ: 158.29, 140.83, 136.95, 130.17, 117.81, 98.63, 34.98, 31.74, 31.36, 29.28, 29.13, 29.06, 22.55, 14.43. MS (ESI) m/z: calcd for $C_{31}H_{46}N_2$: 446.369; found: 447.387 $[M+H]^+$.

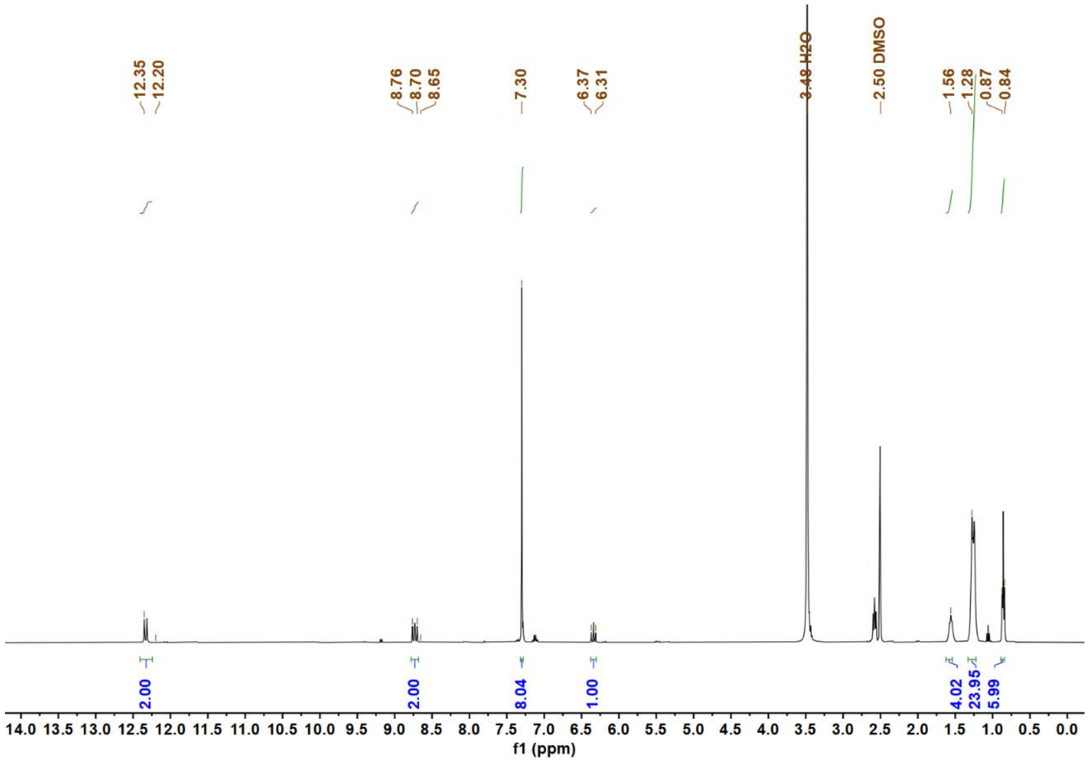

Figure S51. ^{1}H NMR (400 MHz) spectrum of **PN-Boctyl** in d_6-DMSO at 25 ℃.

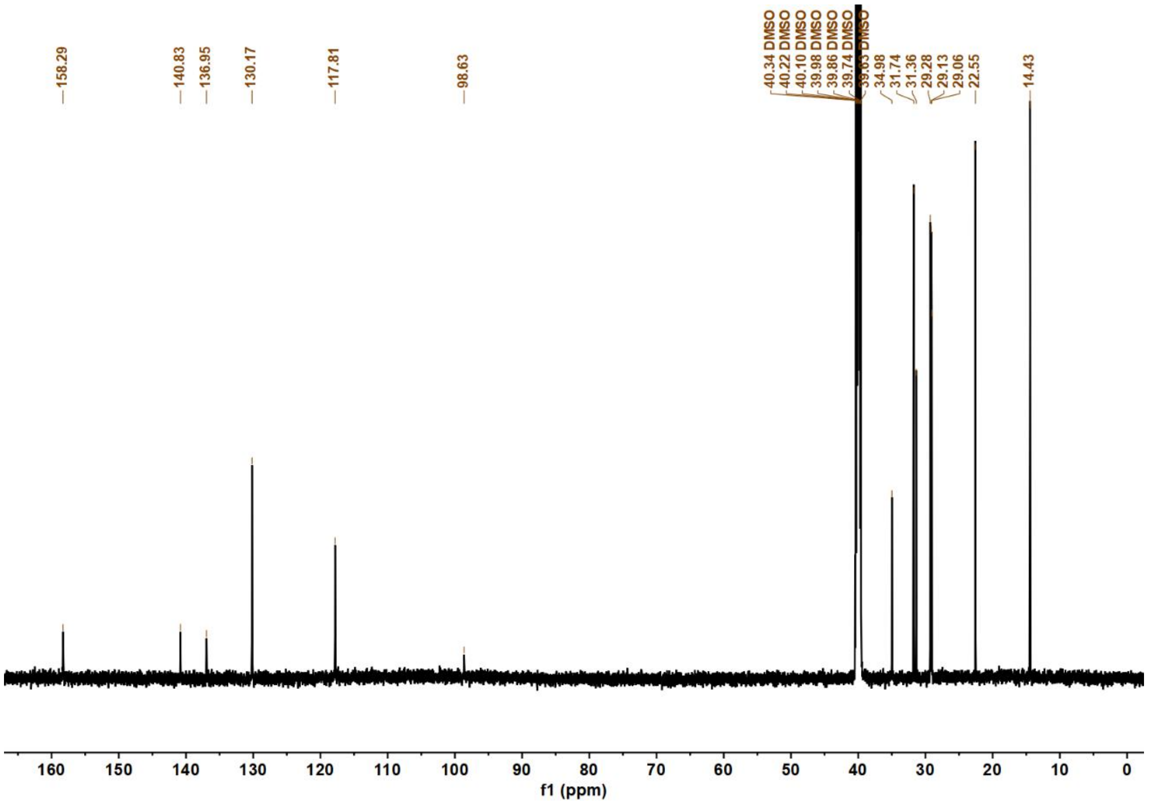

Figure S52. ^{13}C NMR (176 MHz) spectrum of **PN-Boctyl** in d_6-DMSO at 25 ℃.

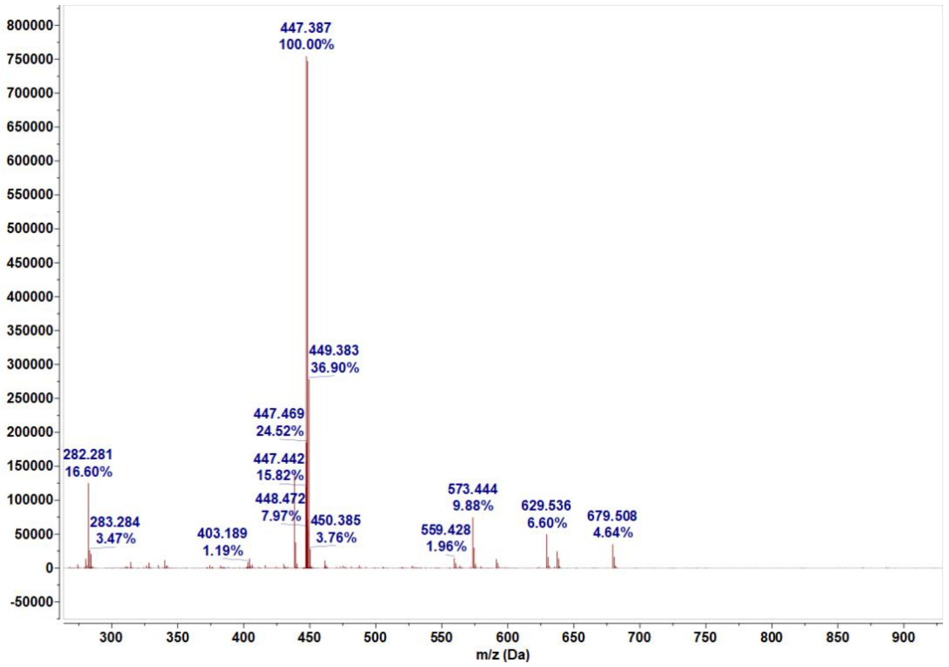

Figure S53. MS spectrum of **PN-Boctyl** Calcd. for $C_{31}H_{46}N_2$: 446.3694, found: 447.387 $[M+H]^+$.

4.4 Synthesis of PN-BIP:

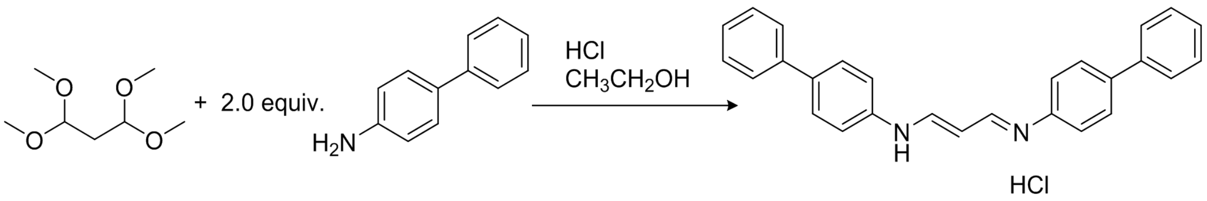

Scheme 5. Synthetic route of **PN-BIP**.

PN-BIP was synthesized according to similar methods as that described for 4.1 synthesis. Briefly, 4-Aminodiphenyl (0.50 g, 3.0 mmol) and tetramethoxypropane (0.25 g, 1.5 mmol) stirred at room temperature for 4 hours were converted to PN-BIP (0.520 g) as an orange powder with a yield of 91.3%. ^{1}H NMR (700 MHz, DMSO-d_6) δ 12.67 (s, 2H), 8.95 (t, J = 12.1 Hz, 2H), 7.81 (d, J = 8.6 Hz, 4H), 7.70 (d, J = 7.2 Hz, 4H), 7.56 – 7.45 (m, 8H), 7.38 (t, J = 7.4 Hz, 2H), 6.54 (t, J = 11.6 Hz, 1H). ^{13}C NMR (176 MHz, d_6-DMSO) δ 158.45, 139.44, 138.47, 138.03, 129.49, 128.54, 128.08, 126.92, 118.39, 99.54, 56.49, 19.03. MS (ESI) m/z: calcd for $C_{27}H_{22}N_2$ 374.181; found: 375.192 $[M+H]^+$.

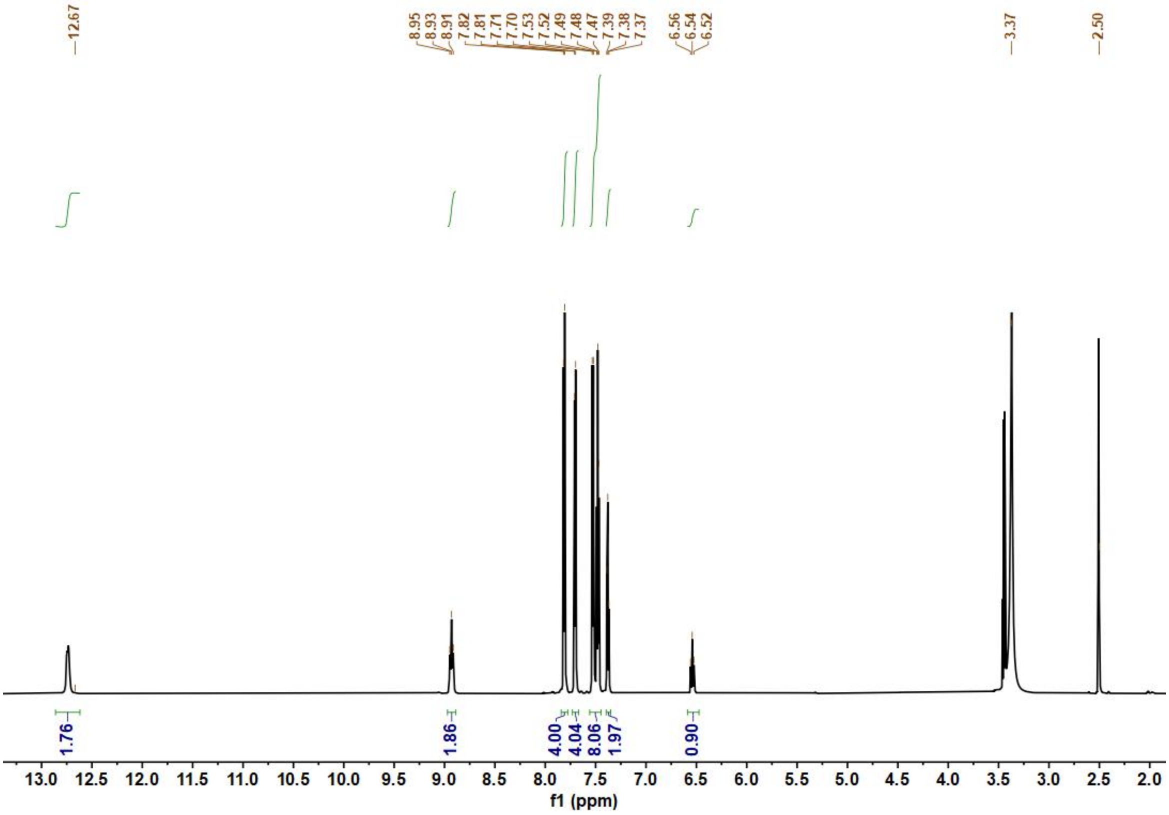

Figure S54. ^{1}H NMR (700 MHz) spectrum of **PN-BIP** in d_6-DMSO at 25 ℃.

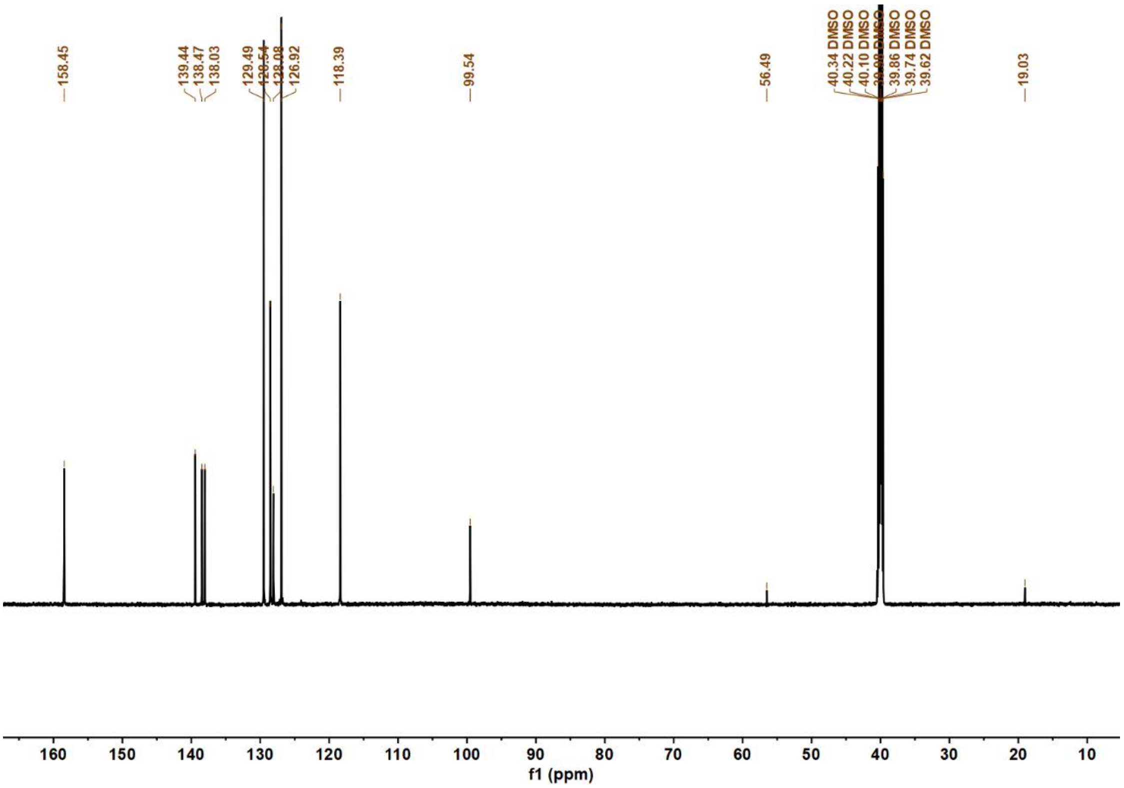

Figure S55. ^{13}C NMR (176 MHz) spectrum of **PN-BIP** in d_6-DMSO at 25 ℃.

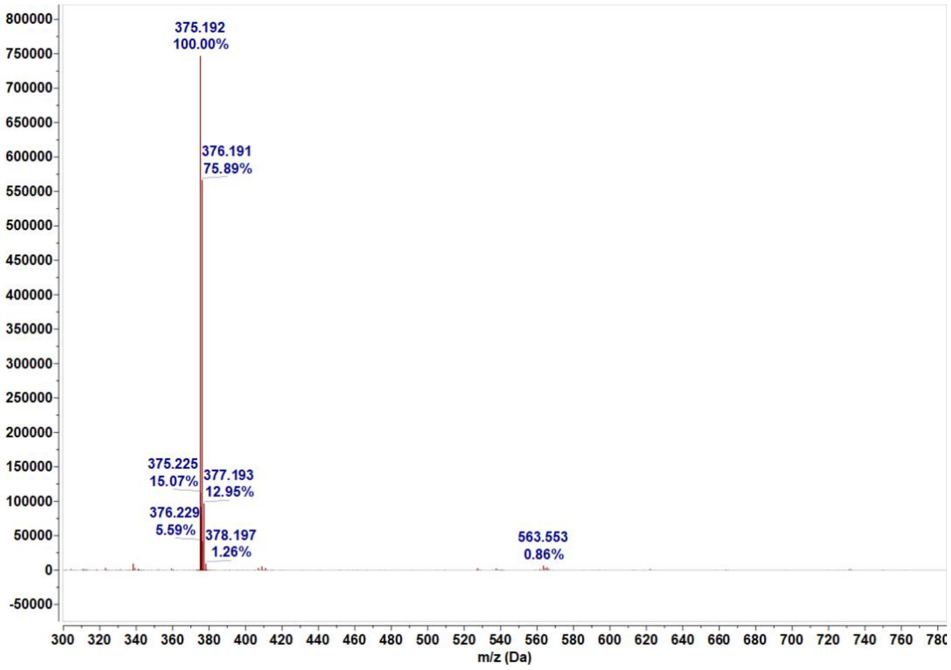

Figure S56. MS spectrum of **PN-BIP** Calcd $C_{27}H_{22}N_2$: 374.1816, found: 375.192 [PN-BIP+H] $^{+}$.

4.5 Synthesis of PN-BP:

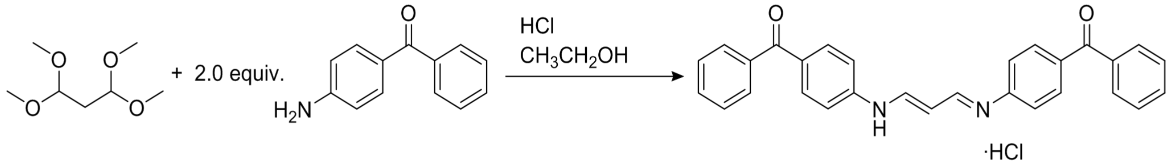

Scheme 6. Synthetic route of **PN-BP**.

PN-BP was synthesized according to similar methods as that described for 4.1 synthesis. Briefly, 4-Aminobenzophenone (0.6 g, 3.0 mmol) and tetramethoxypropane (0.25 g, 1.5 mmol) stirred at room temperature for 4 hours were converted to PN-BP (0.59 g) as a yellow powder with a yield of 90.7%. ^{1}H NMR (400 MHz, DMSO-d_6) δ 13.15 (d, J = 13.2 Hz, 2H), 9.05 (t, J = 12.3 Hz, 2H), 7.89 (d, J = 8.7 Hz, 4H), 7.74 (d, J = 6.9 Hz, 4H), 7.69 (d, J = 7.5 Hz, 2H), 7.63 – 7.56 (m, 8H), 6.68 (t, J = 11.5 Hz, 1H). ^{13}C NMR (100 MHz, d_6-DMSO) δ: 193.05, 155.60, 141.95, 137.57, 134.52, 133.11, 132.34, 130.97, 130.83, 130.55, 129.96, 129.09, 127.66, 117.89, 101.15. MS (ESI) m/z: calcd for $C_{29}H_{22}N_2O_2$ 430.168; found: 431.185 $[M+H]^+$.

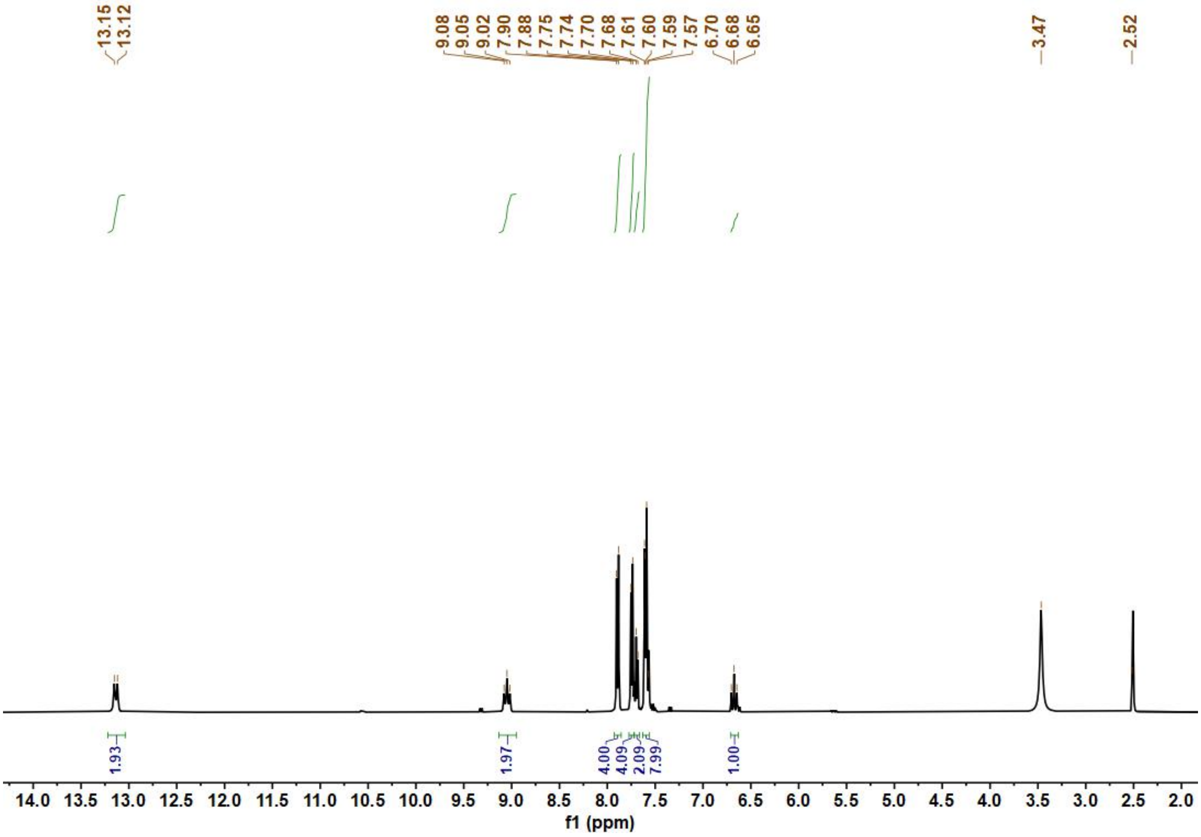

Figure S57. ^{1}H NMR (400 MHz) spectrum of **PN-BP** in d_6-DMSO at 25 ℃.

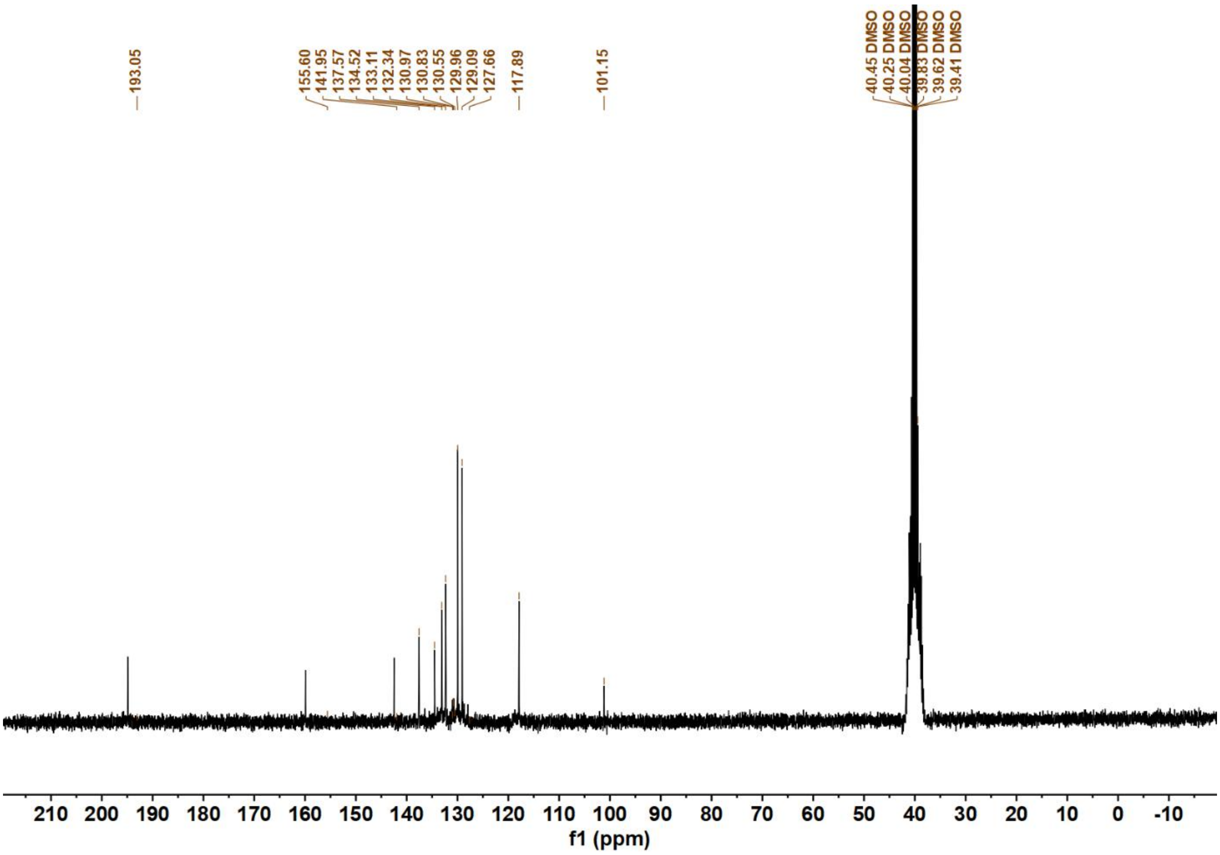

Figure S58. ^{13}C NMR (100 MHz) spectrum of **PN-BP** in d_6-DMSO at 25 ℃.

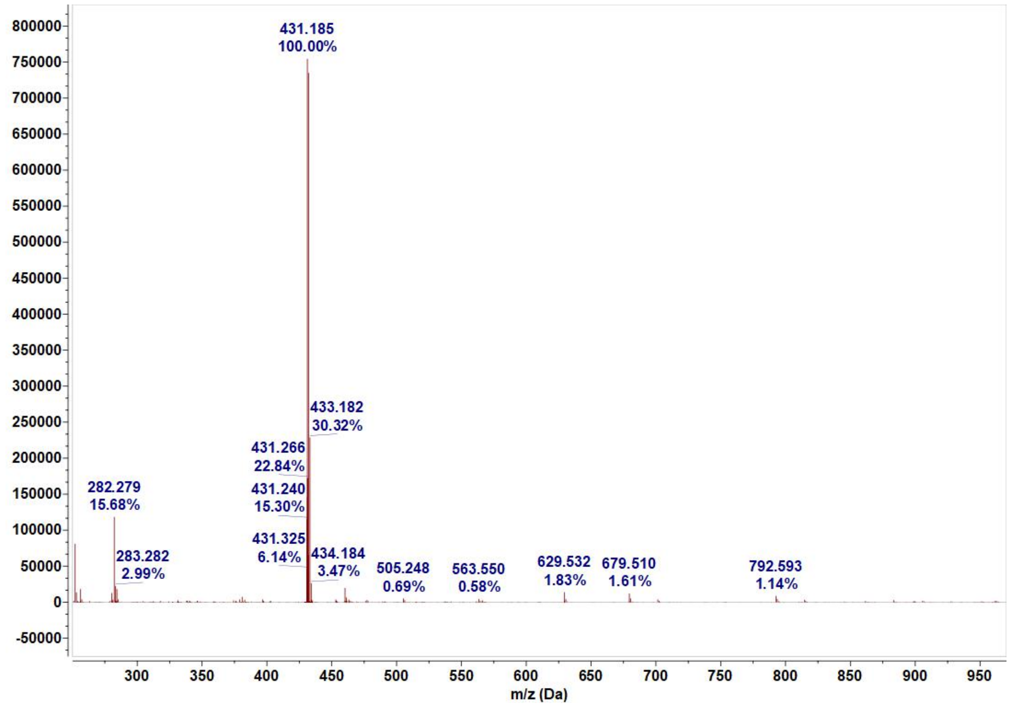

Figure S59. MS spectrum of **PN-BP** Calcd $C_{29}H_{22}N_2O_2$: 430.1681, found: 431.185 $[M+H]^+$.

4.6 Synthesis of PN-TPE:

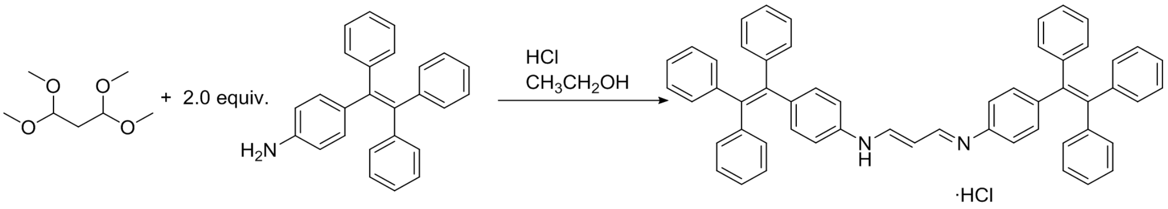

Scheme 7. Synthetic route of **PN-TPE**.

PN-TPE was synthesized according to similar methods as that described for 4.1 synthesis. Briefly, 1-(4-Aminophenyl)-1,2,2-triphenylethene (0.42 g, 1.2 mmol) and tetramethoxypropane (0.10 g, 0.6 mmol) stirred at room temperature for 4 hours were converted to PN-TPE (0.39 g) as a yellow powder with a yield of 88.6%. ^{1}H NMR (400 MHz, d_6-DMSO) δ: 12.17 (d, J = 13.9 Hz, 2H), 8.64–8.58 (m, 2H), 7.21–7.09 (m, 24H), 7.01 (ddd, J_1 = 17.2 Hz, J_2 = 15.2 Hz, J_3 = 8.2 Hz, 16H), 6.22 (t, J = 11.5 Hz, 1H). ^{13}C NMR (100 MHz, d_6-DMSO) δ: 158.76, 149.54, 143.54, 143.47, 143.31, 141.58, 141.43, 140.83, 140.04, 137.26, 132.69, 132.32, 131.17, 131.14, 131.06, 128.46, 128.40, 128.31, 127.22, 119.68, 117.32, 116.33, 111.92, 99.66, 55.42, 18.47. MS (ESI) m/z: calcd for $C_{55}H_{42}N_2$: 730.338; found: 731.355 $[M+H]^+$.

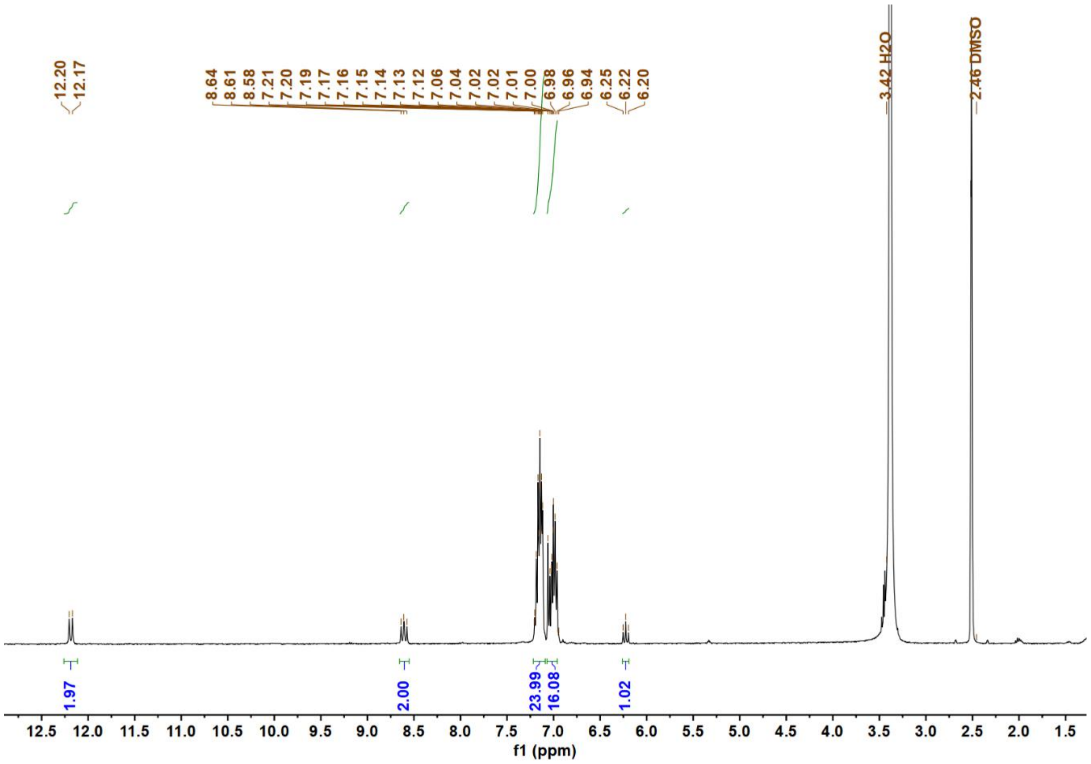

Figure S60. ^{1}H NMR (400 MHz) spectrum of **PN-TPE** in d_6-DMSO at 25 ℃.

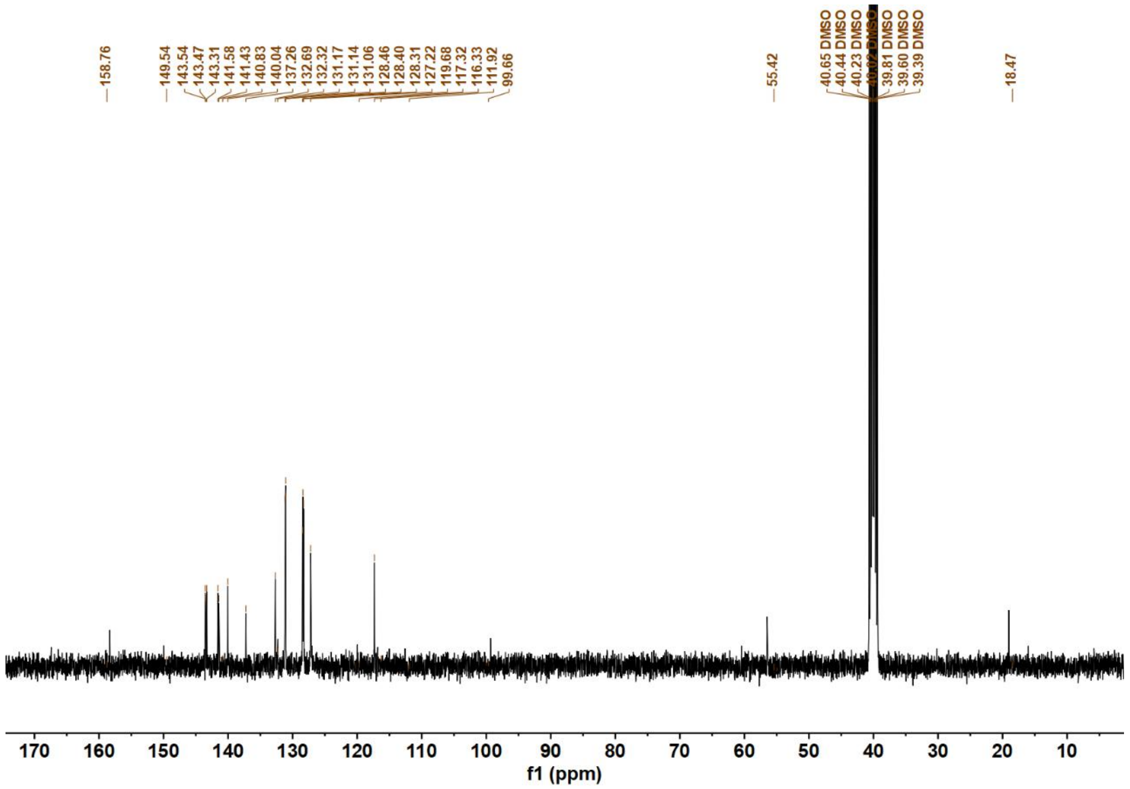

Figure S61. ^{13}C NMR (100 MHz) spectrum of **PN-TPE** in d_6-DMSO at 25 ℃.

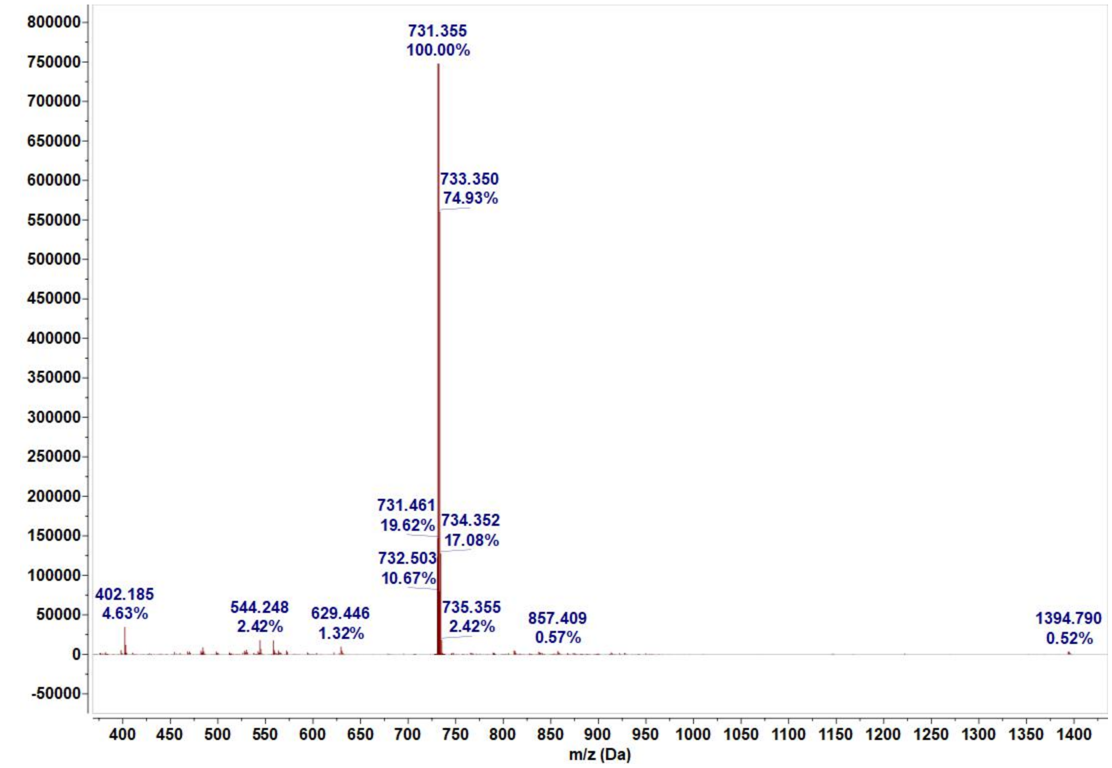

Figure S62. MS spectrum of **PN-TPE**, Calcd for $C_{55}H_{42}N_2$: 730.3381, found: 731.355 $[M+H]^+$.

.

5. References

[1] R. A. Dragovic, C. Gardiner, A. S. Brooks, D. S. Tannetta, D. J. P. Ferguson, P. Hole, B. Carr, C. W. G. Redman, A. L. Harris, P. J. Dobson, P. Harrison, I. L. Sargent, *Nanomed-Nanotechnol* **2011**, *7*, 780-788.

[2] X. Li, D. Wang, Y. Zhang, W. Lu, S. Yang, G. Hou, Z. Zhao, H. Qin, Y. Zhang, M. Li, G. Qing, *Chem. Sci.* **2021**, *12*, 12437-12444.

[3] H. Qiu, Y. Gao, C. E. Boott, O. E. C. Gould, R. L. Harniman, M. J. Miles, S. E. D. Webb, M. A. Winnik, I. Manners, *Science* **2016**, *352*, 697-701.
